# Organoid drug profiling identifies methotrexate as a therapy for SCCOHT, a rare pediatric cancer

**DOI:** 10.1126/sciadv.adq1724

**Published:** 2025-02-26

**Authors:** Seok-Young Kim, Tamar A. E. de Weert, Marijn Vermeulen, Femke Ringnalda, Lennart Kester, Jozsef Zsiros, Selma Eising, Jan J. Molenaar, Karin Sanders, Marc van de Wetering, Hans Clevers

**Affiliations:** ^1^ Princess Máxima Center for Pediatric Oncology, Utrecht, Netherlands.; ^2^ Oncode Institute, Utrecht, Netherlands.; ^3^ Department of Pharmaceutical Sciences, University Utrecht, Utrecht, Netherlands.

## Abstract

Small cell carcinoma of the ovary, hypercalcemic type (SCCOHT) is a rare and lethal tumor in adolescent and young adult patients. Now, there is no standard-of-care treatment for these patients. Reliable models that represent this disease and can be used for translational research are scarce. To model SCCOHTs, we have established eight patient-derived tumoroid lines from tumor lesions of three patients with SCCOHT. The tumoroids recapitulate genomic and transcriptomic characteristics of the corresponding patient tumors and capture intrapatient tumor heterogeneity. Organoid drug profiling using a library of 153 clinical compounds identified methotrexate as an effective and selective drug against SCCOHTs with a clinically relevant IC_50_ of 35 nanomolars. RNA sequencing demonstrated that methotrexate induced TP53 pathway activation and apoptosis. These data underscore that organoid technology can support the design of therapeutic strategies for rare cancers.

## Teaser

Organoid modeling of a rare and aggressive cancer to identify an effective anticancer therapy.

## INTRODUCTION

Small cell carcinoma of the ovary, hypercalcemic type (SCCOHT) is an extremely rare and aggressive tumor in adolescent and young adult (AYA) females. Around ~500 cases of SCCOHTs have been reported in the literature (*1*, *2*). Improving treatments of rare cancers is challenging in terms of conducting clinical trials, while studying the biology of the tumor is hampered by the scarcity of available models (*3*). Now, no standard-of-care exists for patients with SCCOHTs. Without effective treatment guidelines, the overall survival rate of patients with SCCOHTs is 10 to 20% and is particularly dismal in patients with International Federation of Gynecological Oncology (FIGO) stage IV SCCOHTs, who have a survival expectancy of less than a year (*1*, *2*). Surgery, chemotherapy, radiotherapy, and combinations of these treatment modalities have all yielded poor clinical outcomes in the largest retrospective cohort study of SCCOHTs to date (*n* = 293). Data show that surgery and high-dose chemotherapy and/or radiotherapy and autologous stem cell transplantation may benefit some patients with FIGO stage I to III SCCOHTs, while such data are largely lacking for stage IV SCCOHTs (*2*). Preclinical studies on the limited number of conventional cell lines, cell line–derived three-dimensional (3D) spheroids, xenograft models, a patient-derived xenograft model, and primary cultures have suggested that enhancer of zeste 2 polycomb repressive complex 2 subunit (EZH2), fibroblast growth factor receptor (FGFR), and SRC proto-oncogene, non-receptor tyrosine kinase (Src) signaling pathways and deficiency of cyclin dependent kinase 4 and cyclin dependent kinase 6 (CDK4/6) and lysine demethylase 6A (KDM6) are important for proliferation of SCCOHTs and may be exploited to treat this disease (*4*–*8*). However, this requires further validation in prospective clinical trials.

SCCOHTs are diagnosed by loss of SWI/SNF related BAF chromatin remodeling complex subunit ATPase 4 (SMARCA4) and/or SMARCA2 immunostaining. Previous studies have demonstrated that >95% of SCCOHTs harbor somatic and/or germline mutations in the *SMARCA4* gene, which encodes a component of the SWItch/sucrose nonfermentable (SWI/SNF) chromatin remodeling complex. *SMARCA4* alterations in SCCOHTs involve truncating mutations (frameshifts and nonsense mutations) (*9*). SCCOHTs have a very low genetic complexity, and no other recurrent mutations have been identified to date, suggesting that SCCOHT is a monogenic disease driven solely by *SMARCA4* biallelic loss-of-function mutations (*10*–*12*).

Organoid models are 3D stem cell–derived cultures which can grow over long time periods and capture key biological characteristics of the tissue from which they are derived (*13*). Key to this technology is the use of extracellular matrix–like hydrogels [i.e., basement membrane extract (BME)/Matrigel] in combination with a growth factor cocktail tailored to the specific tissue. With recent advances in organoid technology, tumoroids (organoids generated from tumor tissue) can be grown in vitro for a variety of carcinomas and sarcomas (*14*–*19*). Multiple studies have demonstrated that tumoroids can guide treatment strategies tailored to individual tumor types and patients, including (rare) pediatric cancers (*14*, *15*, *17*, *19*). In this study, we aim to develop a protocol to culture SCCOHT tumoroids and to use these tumoroids for compound screens to identify a therapeutic strategy for this essentially untreatable cancer type.

## RESULTS

### Generation of patient-derived tumoroids from three adolescent patients with SCCOHT

We included primary tumor tissues (ovary) and metastatic tumor tissues (sigmoid rectum, uterus, bladder, omentum, omentum deposition flexura lienalis, and pelvis) of three adolescent patients (age range, 16 to 18) with FIGO III (*n* = 2) or FIGO IIIC (*n* = 1) SCCOHTs (patient identifiers; PS-01, PS-02, and PS-03) (alphabet suffix to patient identifiers to indicate different locations) (Table 1). Using organoid medium containing advanced Dulbecco’s Modified Eagle Medium/Ham’s F-12 (DMEM/F12), B27 without vitamin A, N2, epidermal growth factor (EGF), FGF2, insulin-like growth factor 1 (IGF-1), and 0.5% BME, we generated eight tumoroid lines (“O” suffix to sample identifiers to indicate organoids; PS-01a-O, PS-01b-O, PS-01c-O, PS-01d-O, PS-02a-O, PS-02b-O, PS-02c-O, and PS-03b-O) of the nine tumor tissues that we placed in culture, achieving a success rate of 88.8% (8 of 9). Likely due to paucity of biopsy material which was used to initiate organoid culture, PS-03a-O did not propagate. Of note, tumoroid lines of patient PS-01 were generated from before and after cisplatin combination chemotherapy (Table 1). All tumoroid lines could be passaged at least 15 times in organoid medium. Once established, most tumoroids could be passaged without EGF, FGF2, or IGF-1, whereas tumor cell growth of PS-02b and PS-02c remained dependent on either EGF or FGF2 (fig. S1).

**Table 1.** Clinical information of SCCOHT patients in this study. Samples obtained from different locations have alphabet suffixes to patient identifiers. Resulting protein from the indicated SMARCA4 mutations are described in parentheses, where available.

Patient ID	Tumor stage	Sample ID	Location of sampling	Previous treatments (before sampling)	Germline *SMARCA4* mutation	Somatic *SMARCA4* mutation
PS-01	FIGO III C	PS-01a	Ovary	None	c.1189C > T (p.Arg397Ter)	c.2631C > A (p.Tyr877Ter)
		PS-01b	Sigmoid rectum	Cisplatin + etoposide + ifosfamide × 4 cycles		
		PS-01c	Uterus			
		PS-01d	Bladder			
PS-02	FIGO III C	PS-02a	Omentum	Carboplatin + doxorubicine × 1 cycle	c.1002dup (p.Gln335AlafsTer52)	c.2859 + 1_2859 + 2insA
		PS-02b	Omentum deposition flexura lienalis			
				Ifosfamide + etoposide × 1 cycle		
				Doxorubicine + etoposide × 2 cycles		
		PS-02c	Pelvis			
PS-03	FIGO III	PS-03a	Ovary	None	Promoter region deletion at chr19:10,956,000–10,962,000	c.1393G > T (p.Glu465Ter)
		PS-03b	Ovary	Vincristine + cyclophosphamide + doxorubicin × 1 cycle		
				Cisplatin + doxorubicine + ifosfamide × 4 cycles		

**Fig. 1.**
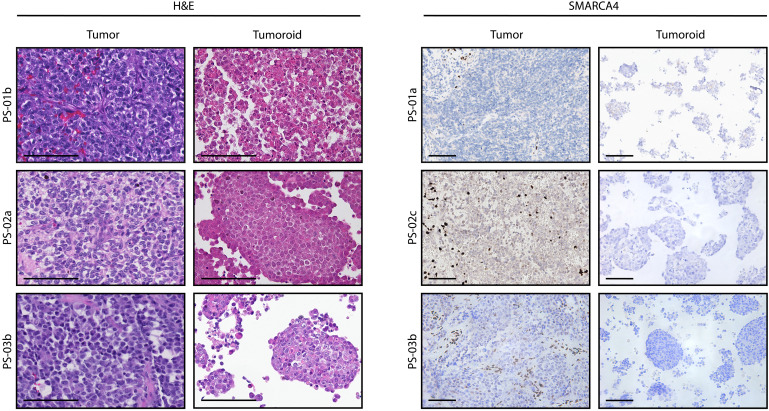
SCCOHT tumoroids recapitulate histopathological characteristics of matching patient tumors. H&E and SMARCA4 immunohistochemistry images on formalin-fixed paraffin embedded slides of SCCOHT patient tumors and matching tumoroids. Lymphocytes and endothelial cells are internal positive controls for SMARCA4 staining. Representative images are shown. Scale bars, 100 μm.

We investigated whether tumoroids recapitulated the histopathological characteristics of SCCOHTs (*12*, *20*). Hematoxylin and eosin (H&E) staining and SMARCA4 immunohistochemistry demonstrated that tumoroids retained the cell morphology of the corresponding patient tumors and confirmed the loss of SMARCA4 protein expression (Fig. 1). In the H&E staining of the PS-01a-T tumor specimen (“T” suffix to sample identifiers to indicate primary tumor tissue), yolk sac elements, mature teratoma elements, and small cell and large cell variants of SCCOHTs were detected, whereas the corresponding tumoroid line (PS-01a-O) retained the cell morphology of the corresponding patient tumor cell compartment (Fig. 1 and fig. S2).

### SCCOHT tumoroids recapitulate genetic characteristics of SCCOHT tumors

**Fig. 2.**
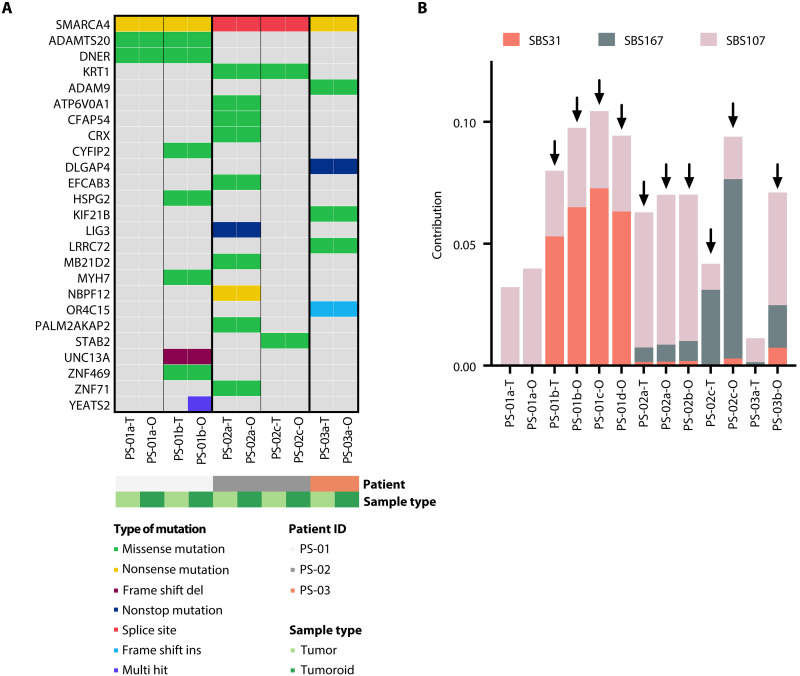
SCCOHT tumoroids recapitulate genetic characteristics of matching patient tumors. (**A**) Nonsynonymous somatic alterations of SCCOHT patient tumors and matching tumoroids detected using WGS. Mutation types, patients, and sample types are color-coded. (**B**) Mutational signature analysis was performed on WGS data of 13 SCCOHT tumors and tumoroids, which detected signatures SBS31, SBS107, and SBS167. SBS31 was detected only in samples which were collected after patients had been treated with platinum therapy (indicated in black arrows). The *y* axis indicates the absolute contribution of each mutational signature in each sample which is proportional to the number of somatic mutations.

To demonstrate that the patient-derived tumoroids preserved genetic characteristics of their matching tumors, we performed whole-genome sequencing (WGS). The tumors in all three patients carried a heterozygous *SMARCA4* germline mutation and a heterozygous *SMARCA4* somatic mutation (Fig. 2A and Table 1). No other recurrent somatic mutations were found in the three patients with SCCOHT. Notably, somatic alterations were shared between tumoroids and corresponding patient tumors, underscoring their genetic similarity (Fig. 2A).

Most previous studies have used whole-exome sequencing or targeted sequencing to investigate genetic characteristics of SCCOHTs with limited information on mutational patterns (*10*–*12*, *21*, *22*). To investigate mutational patterns in SCCOHTs, we extracted mutational signatures from WGS data of SCCOHT tumors and tumoroids in our cohort. Three mutational signatures (SBS31, SBS107, and SBS167) were thus identified (Fig. 2B) (*22*). SBS31, a signature correlated to platinum drug therapy (*22*), was detected in tumors and tumoroids which were derived from postplatinum treatment samples (PS-01b, PS-01c, PS-01d, PS-02a, PS-02b, PS-02c, and PS-03b). SBS107, a recently identified mutational signature which is characterized by C > A base substitutions and high similarity to tobacco signature (SBS4) (*22*), was enriched in all samples. SBS167, again a recently identified mutational signature which is specific for liver tumors and of unknown origin, was highly enriched in PS-02c-T and PS-02c-O derived from a pelvis lesion of PS-02 patient but not in PS-02a-T and PS-02a-O derived from an omentum lesion from the same patient (*22*). Together, these results demonstrated that our tumoroid lines share mutational signatures with their corresponding tumors and recapitulate heterogeneity between tumors.

### SCCOHT tumoroids recapitulate transcriptomic characteristics of SCCOHT tumors

**Fig. 3.**
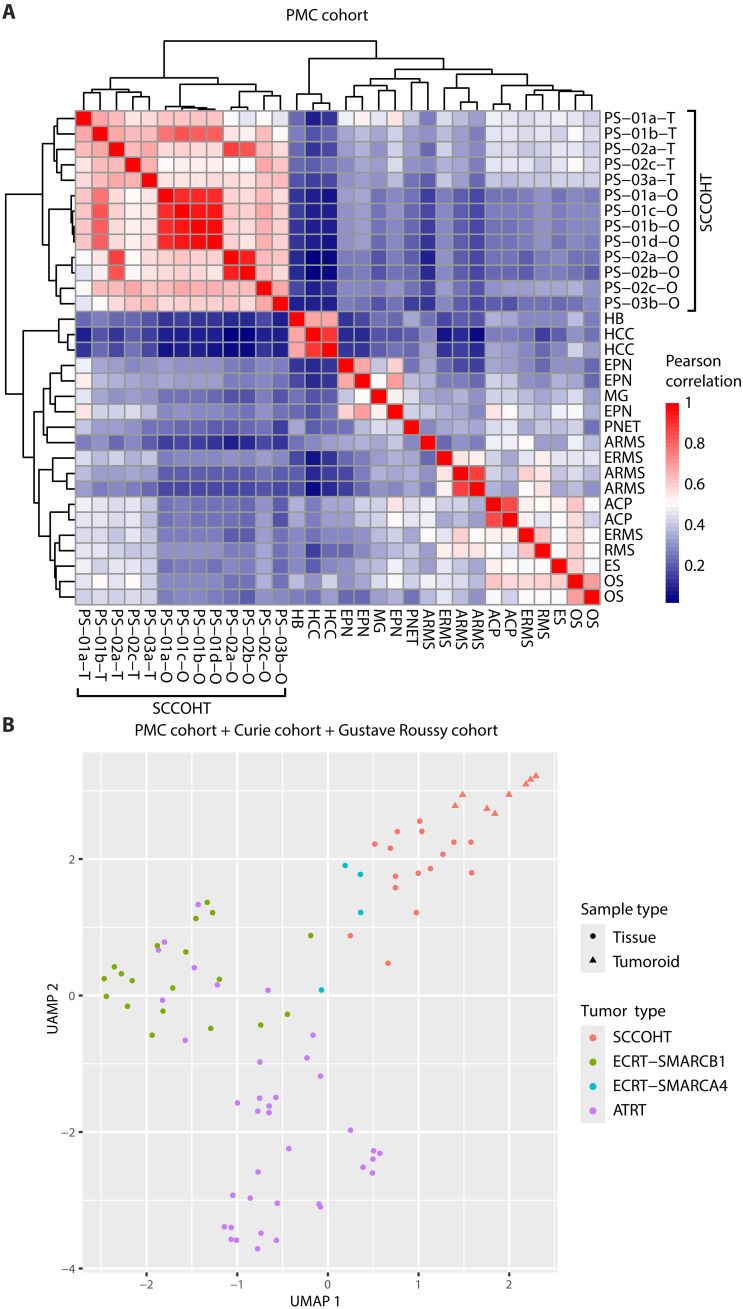
SCCOHT tumoroids recapitulate transcriptomic characteristics of matching patient tumors. (**A**) Correlation heatmap (Pearson correlation) of SCCOHT tumors (*n* = 5), tumoroids (*n* = 8), and various pediatric tumors (*n* = 19) in the PMC cohort based on RNA-seq data. (**B**) UMAP analysis of SCCOHT tumors (*n* = 16), tumoroids (*n* = 8), and SWI/SNF-deficient rhabdoid tumors (*n* = 61) in the PMC, Curie, and Gustave Roussy cohorts based on RNA-seq data. Each dot indicates an individual sample. Tumor type is color-coded. SCCOHT tumoroids (circle) are grouped with SCCOHT tumors (triangle). HB, hepatoblastoma; HCC, hepatocellular carcinoma; EPN, ependymoma; MG, malignant glioma; PNET, primitive neuroectodermal tumor; ARMS, alveolar rhabdomyosarcoma; ERMS, embryonal rhabdomyosarcoma; RMS, rhabdomyosarcoma; OS, osteosarcoma; ES, ewing sarcoma; ACP, adamantinomatous craniopharyngioma; ATRT, atypical teratoid/rhabdoid tumor; ECRT-SMARCB1, SMARCB1-deficient extracranial rhabdoid tumor; ECRT-SMARCA4, SMARCA4-deficient extracranial rhabdoid tumor.

Next, we performed RNA sequencing (RNA-seq) to examine whether SCCOHT tumoroids reflect transcriptomic characteristics of matching patient tumors. Transcriptomic analysis confirmed that, within our Princess Máxima Center (PMC) cohort, the tumoroids were more similar to SCCOHT tumors than to other pediatric tumor types including hepatoblastoma (*n* = 1), hepatocellular carcinoma (*n* = 2), ependymoma (*n* = 3), glioma (n = 1), craniopharyngioma (*n* = 2), primitive neuroectodermal tumor (*n* = 1), and various types of sarcomas (*n* = 9) (Fig. 3A) (*23*). *SMARCA4* and *SMARCA2* mRNA expression levels were significantly lower in SCCOHT tumor tissues and tumoroids compared to other pediatric tumor tissues (*P* values < 0.0001) based on RNA-seq data (fig. S3A), which was validated by reverse transcription quantitative polymerase chain reaction (RT-qPCR; fig. S3B). Accordingly, gene set enrichment analysis (GSEA) demonstrated that previously reported target genes of *SMARCA4* are expressed significantly lower in SCCOHT tumor tissues and tumoroids compared to other pediatric tumor tissues (fig. S3C).

To further validate our finding, we included RNA-seq datasets (Curie cohort and Gustave Roussy cohort) (*24*) of SCCOHTs (*n* = 11), SMARCB1-deficient atypical teratoid/rhabdoid tumors (ATRTs; *n* = 38), SMARCB1-deficient extracranial rhabdoid tumors (ECRT-SMARCB1; *n* = 19), and SMARCA4-deficient extracranial rhaboid tumors (ECRT-SMARCA4; *n* = 4) in our transcriptomic analysis. We used limma to remove batch effect (*25*) while keeping biological covariates [tumor type and sample type (tumors versus tumoroids)], before performing UMAP (Uniform Manifold Approximation and Projection) analysis. UMAP analysis demonstrated that SCCOHT tumors from different cohorts were grouped together, showing that the batch effect was successfully regressed out (Fig. S4A). Furthermore, we observed distinct groups of SCCOHTs, ECRT-SMARCB1, ECRT-SMARCA4, and ATRTs and proximity of the ECRT-SMARCA4 group to both ECRT-SMARCB1 and SCCOHT groups, as previously described (Fig. 3B and fig. S4B) (*24*). Notably, SCCOHT tumoroids were grouped with SCCOHT tumors and were separated from various other tumor types including SWI/SNF-deficient rhabdoid tumors (Fig. 3B and fig. S4B). Together, these results showed that the patient-derived tumoroids faithfully recapitulated transcriptomic characteristics of the SCCOHT tumors.

### Methotrexate is a potent and selective small-molecule growth inhibitor of SCCOHTs

**Fig. 4.**
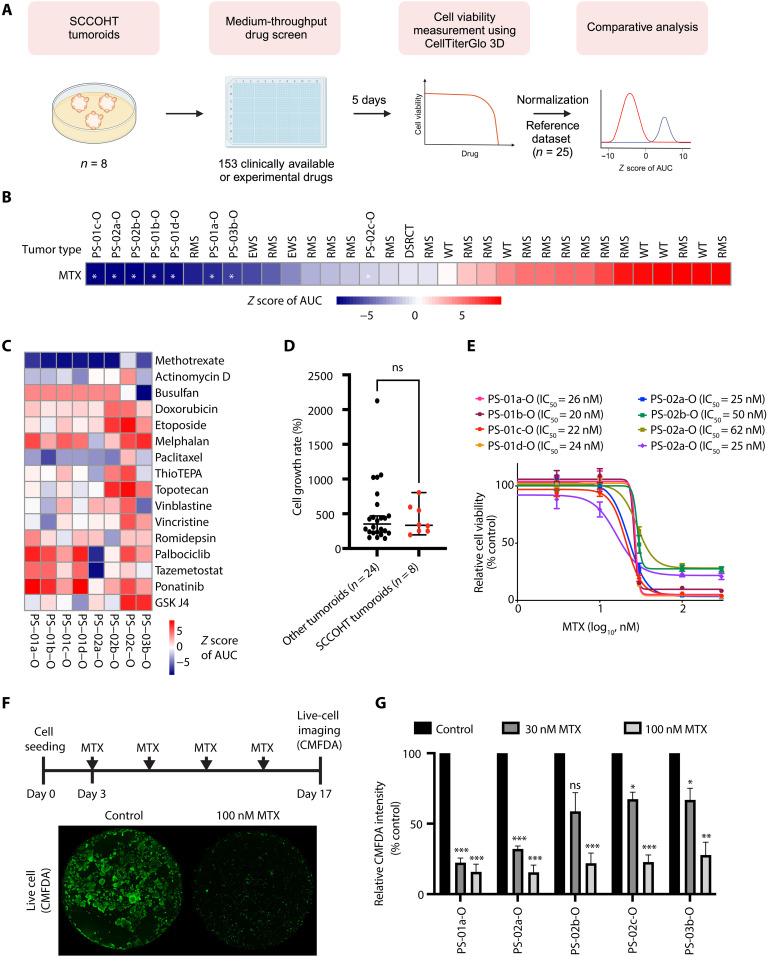
Medium-throughput drug screen identifies methotrexate as a selective small-molecule inhibitor against SCCOHT tumoroids. (**A**) A scheme to identify a selective and effective agent against SCCOHTs. Eight SCCOHT tumoroids were screened at medium-throughput using 153 clinically available drugs. Cell viability was measured at day 5. AUC values were obtained for each single drug. These values were normalized to AUC values of drugs at day 5 (*z* score) in 25 other tumoroids of Wilms tumor (WT), rhabdomyosarcoma (RMS), DSRCT, and Ewing sarcoma (EWS). Multiple *t* test was performed to compare the normalized AUC values between SCCOHT tumoroids and the rest. BioRender was used to create this image. (**B**) Heatmap illustrating the *z* score of methotrexate (MTX) across a panel of tumoroids (*n* = 33). Decrease (blue) in the values indicates drug sensitivity, and increase (red) indicates drug insensitivity. (**C**) Heatmap illustrating *z* score AUC values of MTX and selected drugs against SCCOHTs. (**D**) Growth rates of pediatric tumoroids (*n* = 24) from the reference dataset and SCCOHT tumoroids (*n* = 8). (Mann-Whitney *U* test: ns, nonsignificant). (**E**) Validation screen of MTX efficacy in eight SCCOHT tumoroids at day 5. The IC_50_ value of MTX for each line is indicated above. (**F**) A scheme for examining long-term efficacy of methotrexate (MTX) in SCCOHT tumoroids. Tumoroids were cultured in organoid medium containing DMSO and 30 or 100 nM MTX for 2 weeks. Live cells in a whole-well were imaged using CellTracker green CMFDA. Representative fluorescent images of PS-03b-O from (F) are shown below. (**G**) Quantification of live-cell imaging in five representative SCCOHT tumoroids following (F). Fluorescence intensity was normalized to DMSO control in each line [one-way analysis of variance (ANOVA) test with Dunnett’s posttest: *P < 0.05, **P < 0.01, and ***P < 0.005). Results from biological triplicates are shown in (E) to (G).

We used the SCCOHT tumoroids to identify an effective and selective small-molecule compound for SCCOHTs. We performed medium-throughput drug screening (5 days of drug exposure) of a compound library consisting of 153 clinically available cytotoxic and targeted agents on eight SCCOHT tumoroid lines. Subsequently, we compared the drug responses of SCCOHT tumoroids with those of 25 tumoroids of other pediatric or AYA cancers [Wilms tumors, rhabdomyosarcomas, Desmoplastic small round cell tumors (DSRCTs), and Ewing sarcomas] using *z* scores of AUC (area under the curve) (Fig. 4A) (*26*–*29*). The SCCOHT tumoroids showed a distinct drug response pattern compared with other tumoroids (fig. S5).

Multiple *t* testing analysis identified methotrexate, a cytotoxic agent that targets dihydrofolate reductase, methylenetetrahydrofolate reductase (MTHFR), 5-aminoimidazole-4-carboxamide ribonucleotide formyltransferase/IMP cyclohydrolase (ATIC), and thymidylate synthetase (TYMS), as the top hit for SCCOHT tumoroids (adjusted *P* value, 0.000296) (Fig. 4B and table S1) (*30*). Furthermore, methotrexate was more effective against the SCCOHT tumoroids than the drugs which are now in clinical trials (tazemetostat and palbociclib) (*1*), have been conventionally used to treat SCCOHTs (actinomycin D, busulfan, doxorubicin, etoposide, melphalan, paclitaxel, thioTEPA, topotecan, vinblastine, vincristine, and romidepsin) (*1*, *2*, *4*, *7*, *31*), or have recently demonstrated preclinical efficacy (ponatinib and GSK J4) (Fig. 4C) (*5*, *8*). We observed that PS-02a-O and PS-03b-O were relatively sensitive (*z*AUC < 0) to tazemetostat, an EZH2 inhibitor (*4*), although there was no correlation between the response to tazemetostat and *EZH2* expression in SCCOHT tumoroids (fig. S6A and table S2). PS-02a-O was also relatively sensitive to palbociclib, a selective CDK4/6 inhibitor (fig. S6B and table S2) (*7*). Low expression of *CCND1* mRNA has previously been described to be associated with good palbocliclib efficacy (*7*). *CCND1* expression was relatively low not only in PS-02a-O but also in two other lines (PS-02b-O and PS-03b-O) that responded poorly to palbocliclib (fig. S6B and table S2). In addition, PS-01a-O, PS-01d-O, and PS-02b-O were relatively sensitive to GSK J4, a selective KDM6 inhibitor (fig. S6, C and D) (*8*). However, median inhibitory concentration (IC_50_) values of GSK J4 were higher in these SCCOHT tumoroids than those previously reported in SCCOHT patient–derived xenograft-derived primary cultures (2.20 to 2.97 μM versus 0.07 to 0.26 μM) (*8*). The mRNA expression levels of *KDM6A* and *KDM6B* were comparable among all SCCOHT tumoroids and not correlated to GSK J4 efficacy (fig. S6, C and D).

It is generally believed that cytotoxic therapies are most effective against fast-dividing cells (*32*). To investigate a possible correlation between methotrexate sensitivity and cell growth rates, we examined cell growth rates of SCCOHT tumoroids (*n* = 8) and other tumoroids (*n* = 24) where data are available. There was no significant difference between the cell growth rates of these two groups (Fig. 4D). In addition, most cytotoxic therapies included in our screen were essentially ineffective against SCCOHT tumoroids (table S1), indicating a specific effect of methotrexate against SCCOHT tumoroids.

Our validation screen confirmed high efficacy of methotrexate on SCCOHT tumoroids, with IC_50_ values ranging between 20 and 62 nM (Fig. 4E). Notably, these values are 21- to 68-fold lower than the maximal plasma concentrations of methotrexate-treated patients (*33*). When tested over a period of 2 weeks (Fig. 4F), methotrexate potently inhibited tumor cell growth, demonstrating longer-term efficacy (Fig. 4, F and G). Drug responses can differ between tumor cells grown in 3D or 2D (*15*). Therefore, we tested whether growing the SCCOHT tumoroids in 3D or 2D affects methotrexate efficacy (fig. S7). Our results showed that both 3D and 2D tumoroids have comparable responses to methotrexate (Fig. 4E and fig. S7), although IC_50_ values of methotrexate were marginally decreased in 2D compared with 3D (fig. S7). Together, these results demonstrated that methotrexate is a selective and effective drug against SCCOHT tumoroids.

### Methotrexate activates the P53 pathway in SCCOHT tumoroids

**Fig. 5.**
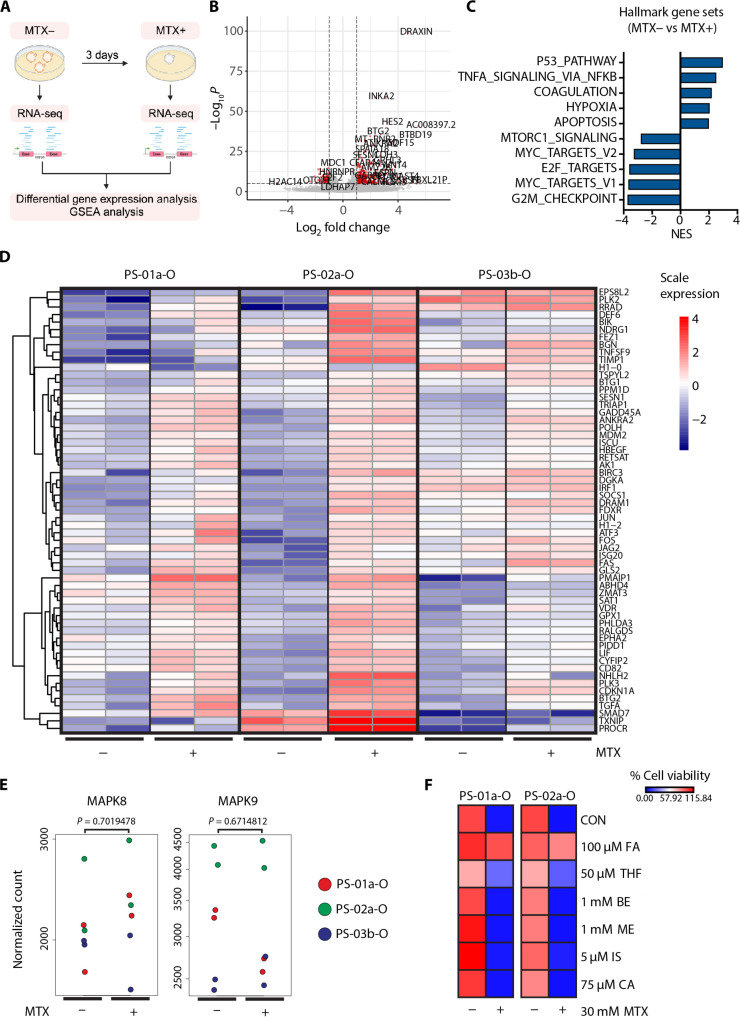
Methotrexate activates TP53 pathway and induces cell cycle arrest and apoptosis in SCCOHT tumoroids. (**A**) Experimental scheme to investigate MTX-induced transcriptomic changes in SCCOHT tumoroids. Three representative tumoroids (PS-01a-O, PS-02a-O, and PS03b-O) were treated with 100 nM MTX for 3 days. RNA-seq was performed on organoids before (MTX−) and after (MTX+) treatment. BioRender was used to create this image. (**B**) Volcano plot illustrating significantly differentially expressed genes (DEGs) (log_2_ fold change > |1|, adjusted *P* value < 0.05) in MTX− versus MTX+. (**C**) GSEA using hallmark gene sets. Top five and bottom five results based on normalized enrichment score (NES) are shown. (**D**) Heatmaps illustrating significant DEGs (MTX− versus MTX+) in hallmark P53 pathway and apoptosis in SCCOHT tumoroids. (**E**) *MAPK8* and *MAPK9* gene expressions in MTX− and MTX+ RNA-seq datasets. Each dot indicates a single RNA-seq run from an organoid line. Organoid lines are color labeled. Adjusted *P* values calculated from Walt test are shown. (**F**) Bar graph showing cell viability of SCCOHT tumoroids (PS-01a-O and PS-02a-O) treated with folate [folic acid (FA) and tetrahydrofolic acid (THF)], methyl donor [betaine (BE) and methionine (ME)], and adenosine receptor blocker [istradefylline (IS) and caffeine (CA)] in combination with DMSO control or 30 nM MTX. Results from biological duplicates are shown.

To investigate effects of methotrexate on SCCOHT tumoroids, we performed RNA-seq on representative SCCOHT tumoroids (PS-01a-O, PS-02a-O, and PS-03b-O) before and after (day 3) treatment with methotrexate (Fig. 5A). Differential gene expression analysis revealed that 1072 genes were up-regulated (log_2_ fold change > 1, adjusted *P* value < 0.05), and 205 genes were down-regulated (log_2_ fold change < −1, adjusted *P* value < 0.05) after methotrexate treatment (Fig. 5B and table S3). GSEA analysis using hallmark gene sets demonstrated that methotrexate treatment induced activation of the P53 pathway and apoptosis and down-regulation of cell cycle–related pathways (G_2_M checkpoint, MYC_targets_V1, E2F targets, and MYC_targets_V2) (Fig. 5C and table S4). Notably, methotrexate treatment significantly increased (log_2_ fold change > 2, adjusted *P* value < 1.0 × 10^−5^) transcriptional targets of P53 which inhibit cell cycle progression (*CDKN1A* and *BTG2*) or which are proapoptotic [phorbol-12-myristate-13-acetate-induced protein 1 (PMAIP1) and fatty acid synthase] (Fig. 5D and table S3) (*34*–*38*). These results suggested that a clinically relevant dose of methotrexate may elicit a cell cycle arrest and apoptosis in SCCOHT tumoroids by P53 pathway activation.

Methotrexate has been proposed to act by inhibiting folate-dependent purine and pyrimidine nucleotide production, by inducing adenosine release into the extracellular space, by decreasing formation of methyl donors, and/or by increasing apoptosis via mitogen-activated protein kinase 8 (MAPK8) and MAPK9 in rheumatoid arthritis and cancer cells (*30*, *39*–*45*). We observed that *MAPK8* and *MAPK9* expressions were not changed upon methotrexate treatment (*P* = 0.702 and 0.671, respectively) (Fig. 5E). To further understand through which of these pathways methotrexate inhibits SCCOHT tumoroid cell growth, we tested how folic acid, tetrahydrofolic acid (coenzyme in purine and pyrimidine production), betaine and methionine (methyl donors) (*41*, *42*), istradefylline, and caffeine (adenosine receptor antagonist) (*46*, *47*) affected SCCOHT tumoroid viablity with or without methotrexate treatment (Fig. 5F). Notably, addition of folic acid and tetrahydrofolic acid to SCCOHT tumoroids markedly increased IC_50_ values of methotrexate, whereas addition of methyl donors and adenosine receptor antagonists had no effects on methotrexate efficacy. In addition, hypoxanthine, a purine derivative, but not thymidine, a pyrimidine deoxynucleotide (*48*), reduced the effect of methotrexate (fig. S8), implying that methotrexate acts by inhibiting purine production via the folate pathway in SCCOHTs.

## DISCUSSION

In this study, we show that SCCOHT tumoroids can be generated from adolescent patient tumor tissues with a high success rate, can be expanded for a long time in defined medium, and faithfully reflect biological characteristics of the corresponding patient tumors. This approach generates translational research platforms for SCCOHTs, rare and aggressive tumors for which clinically relevant in vitro and in vivo models are scarce.

Our WGS analysis showed that the analyzed SCCOHT tumors and tumoroids harbor recurrent *SMARCA4* mutations and are of low genetic complexity, in agreement with previous studies (*10*–*12*). We detected treatment-related and patient-specific heterogeneity of mutational signatures in SCCOHTs. We observed SBS107, a signature of unknown origin but known to be prevalent in kidney, liver, and bladder cancers, in all SCCOHT samples (*22*). The (unknown) mechanism that causes this signature might also cause somatic *SMARCA4* nonsense mutations in SCCOHTs, as this concerns a C > A base substitution in two of our three patients (c.2631C > A in PS-01 and c.1393G > T in PS-03). Understanding the etiology and molecular mechanism of SBS107 may provide insight into tumor initiation of SCCOHTs. We observed mature teratoma and yolk sac tumor elements in PS-01a-T sample, in alignment with (*49*). The association of SCCOHTs with teratoma and yolk sac tumor elements and expression of germ cell markers in SCCOHTs (*49*) may suggest that these tumor types share the germ cell origin (*49*, *50*).

Using our tumoroids with their diverse genetic and transcriptomic backgrounds, we demonstrate that methotrexate may present a clinically available, effective, and potentially safe therapeutic strategy for SCCOHT. We demonstrated short-term and long-term in vitro efficacy of methotrexate against SCCOHT tumoroids at a clinically relevant dose. Of note, IC_50_ values of methotrexate in SCCOHT tumoroids were comparable to or lower than those of in vitro models of acute lymphoblastic leukemia, breast cancer, and head and neck cancer (IC_50_ values: 12.8 to 164 nM), all cancer types in which methotrexate has demonstrated clinical efficacy (*51*–*58*). Otte *et al.* (*59*) described that epothilone B, methotrexate, topotecan, and paclitaxel could be effective therapies against SCCOHTs based on a limited drug screening with chemotherapies in a SCCOHT-1 cell line and other two ovarian cancer cell lines (*59*). Among these four drugs, three (methotrexate, topotecan, and paclitaxel) were included in our medium-throughput drug screen. Mean normalized AUC values of both methotrexate and paclitaxel were < 0, although statistical significance was reached only in methotrexate (table S1). Furthermore, IC_50_ values of paclitaxel in some lines (PS-01a-O, PS-01d-O, and PS-02a-O, PS-02c-O) were higher than those of other tumor cell lines (2.5 to 7.5 nM) (table S2) (*60*), which may suggest limited efficacy of paclitaxel against SCCOHT tumor cells. Previous studies have demonstrated preclinical efficacy of tazemetostat, palbociclib, ponatinib, and GSK J4 (*4*, *5*, *7*, *8*). We observed that PS-02a-O expressed *EZH2* and low level of *CCND1* and was sensitive to both tazemetostat and palbociclib, as previously described for conventional SCCOHT cell lines (*4*, *7*). However, *CCND1* expression did not correlate with palbociclib efficacy in other SCCOHT tumoroid lines. In addition, *EZH2* expression did not correlate with tazemetostat efficacy in SCCOHT tumoroids (fig. S6, A and B), which was in alignment with the study of Wang *et al.* (*61*). These results and the early result from an ongoing phase 2 trial (NCT02601950) where a subset of patients with SCCOHT achieved a partial response or stable disease after tazemetostat treatment (*1*) may suggest that additional predictive biomarkers are needed for these drugs.

Furthermore, our data show that methotrexate acts via the folate pathway and suggests that genomic damage resulting from methotrexate treatment activates the P53 pathway and induces cell cycle arrest and apoptosis in SCCOHT tumoroids. In line with this, Huang *et al.* (*62*) demonstrated, in a non–small cell lung cancer cell line, that methotrexate can activate the P53 pathway in solid tumors. Previous studies have demonstrated that purine metabolism can be a vulnerability for cancer cells (*63*) and that SWI/SNF mutations may reprogram metabolic process including nucleotide synthesis in cancer cells (*64*–*66*). These studies may suggest that *SMARCA4* mutation in SCCOHT reprograms purine metabolism via folic acid pathway. However, this hypothesis can only be verified in a careful examination of metabolomic changes induced by SMARCA4 reexpression in SCCOHT tumoroids. In addition, why SCCOHT tumoroids are more sensitive to methotrexate than tumoroid models of other pediatric cancer types remains to be studied.

We are now planning clinical testing of a methotrexate-based therapeutic regimen for patients with this rare yet highly malignant cancer. We acknowledge that case reports have described methotrexate to have limited clinical efficacy in three case reports including patients with various ages, tumor stages, and treatment histories (*67*, *68*). Efficacy of single agent methotrexate treatment needs to be tested in a larger cohort of tumoroids that are generated from young adult or low-stage SCCOHT patients.

In conclusion, we have generated SCCOHT tumoroids that capture tumor heterogeneity and patient treatment history. Drug profiling in diverse SCCOHT tumoroids can help design treatment strategies for patients with this rare cancer type who are in dire need of a cure.

## MATERIALS AND METHODS

### Patient consent and organoid culture

Tumor tissues obtained via surgical resection or diagnostic biopsies were included from three patients [PS-01 (M523AAB), PS-02 (M051AAB), and PS-03 (M348AAD)] as part of the biobank initiative of the Princess Máxima Center (PMC) for Pediatric Oncology, Utrecht, the Netherlands (remaining tumor samples). The biobanking initiative was approved by Medical Ethics Committee NedMec, and use of the human biologiical material and data for this project was approved by the PMC Biobank and Data Access Committee. All patients and/or their legal representatives signed informed consent to have tumor samples taken for biobank usage. Tumor tissues were cut into small pieces using scalpels. The resulting small tissue pieces were cultured in suspension with 0.3% BME (#3533-005-2, Bio-Techne, Minneapolis, MN, USA) in six-well plates (Sarstedt, Nümbrecht, Germany). The SCCOHT tumoroid culture medium included advanced DMEM-F12 (Gibco, Waltham, MA, USA) containing 1× N2 (Gibco), 1× B27 without vitamin A (Gibco), 1× penicillin/streptomycin (Gibco), 10 mM Hepes (Gibco), 1.25 mM *N*-acetylcysteine (Sigma-Aldrich, Burlington, MA, USA), 1× GlutaMAX (Gibco), EGF (10 ng/ml; #AF-100-15, PeproTech, Rocky Hill, NJ, USA), FGF2 (10 ng/ml; #100-18B, PeproTech), and IGF-1 (100 ng/ml; #100-11, PeproTech). Organoids were routinely passaged at a 1:10 to 1:15 split ratio weekly or once every other week.

After tumoroids were established in the above-mentioned medium (>passage 4), effects of growth factors on tumor cell growth were examined by culturing tumoroids in the organoid medium removing EGF, FGF2, and IGF-1 from the tumoroid culture medium and subsequently adding them one by one or in combination for up to 60 days. Cells were passaged at the same split ratio for all conditions when reaching high confluency (>90%). Experiments were performed in biological duplicates.

### H&E staining and immunohistochemistry

Tumoroids were collected and centrifuged at 200*g* for 3 min before being washed with cold phosphate-buffered saline (PBS). Tumoroids were fixed in 10% neutral formaline overnight at 4°C. Then, tumoroids were washed with PBS (three times, 30 min each) and incubated in 70% ethanol overnight at 4°C. Next, tumoroids were incubated in 96% ethanol-eosin (30 min), 96% ethanol (briefly), 100% ethanol (three times, 30 min each), 100% butanol (three times, 30 min each), and 100% paraffin (three times, 30 min each). Tumoroids were embedded in paraffin using the Histo-Core Arcadia H (Leica Biosystems, Nussloch, Germany), and paraffin blocks were cut into 4-μm slides using the Rotary Microtome Microm HM355S (Thermo Fisher Scientific, Waltham, MA, USA).

H&E staining and immunohistochemstry were performed as previously described (*27*). SMARCA4 polyclonal antibody (21634-1-AP, Proteintech) and alpha fetoprotein (AFP) antibody (PA0963, Leica Biosystems) were used according the manufacturer’s recommendations.

### Whole-genome sequencing

Genomic DNA was extracted using a QIAGEN blood and tissue DNA kit (QIAGEN, Hilden, Germany). DNA library preparation was done using the KAPA HyperPlus kit (Roche, Basel, Switzerland) or TruSeq DNA PCR-Free (PS-03b; Illumina, San Diego, CA, USA) according to the manufacturer’s instructions. Tumors and whole blood samples were sequenced at 60× or 90× base coverage and organoids at 30× base coverage on an NovaSeq 6000 (Illumina). WGS data were processed as per the GATK 4.0 workflow based on a wdl and cromwell workflow. Sequence reads were mapped to the human reference gnome (GRCh38) using the Burrows-Wheeler Aligner (*69*). Variants were called using GATK HaplotypeCaller. Variants were filtered when the following criteria were met: Qual by Depth (QD) < 2.0, Fisher Strand bias (FS) > 60.0, Mapping Quality (MQ) < 40.0, MQRankSum < −12.5, ReadPosRankSum < −8.0, and HaplotypeScore >13.0, and indel variants were filtered when the following criteria were met: QD < 2.0, FS > 200.0, ReadPosRankSum < −20.0. Somatic single nucleotide variants (SNVs) and indels were called using Mutect2 and annotated using Ensembl Variant Effect Predictor (VEP) v106. We considered somatic mutations which (i) have allele frequency of ≤0.01 in 1000 Genomes phase 3 of global and European population, (ii) are novel or reported in COSMIC v92, and (iii) have variant allele frequency of ≥0.25.

### RNA sequencing

Total RNA was extracted from tumoroid lines using TRIzol (Thermo Fisher Scientific) following the manufacturer’s instructions. Total RNA was extracted from tumors using the AllPrep DNA/RNA/miRNA universal Kit (QIAGEN) following the manufacturer’s instructions. RNA-seq analysis of tumor tissues and tumoroids, unless specified, was performed as previously described (*23*). Briefly, cDNA libraries were generated using the KAPA RNA HyperPrep Kit with RiboErase (Roche) and sequenced on a NovaSeq 6000 (2 × 150 bp) (Illumina, San Diego, CA). RNA-seq analysis on tumoroids before and after methotrexate treatment (100 nM for 3 days) was performed by the Utrecht Sequencing Facility. cDNA libraries were generated using the TruSeq stranded polyA kit (Illumina) and sequenced on a NextSeq2000 (2 × 50 bp) (Illumina). Resulting raw sequencing reads from both datasets were mapped to the human reference gnome (GRCh38) and gencode version 29 using Star version 2.7.0f (*70*). Raw expression counts were determined at a gene level using Subread Counts (*71*). Gene level raw expression counts and fragments per kilobase of transcript per million mapped reads (FPKM) of 19 pediatric tumor tissues were obtained from the previous study (table S5) (*23*). *SMARCA4* and *SMARCA2* mRNA expressions in samples were shown in log-normalized FPKM counts in fig. S3A.

The transcriptomic profiles of SCCOHT tumoroids were compared with those of SCCOHT tumors and other pediatric tumors at our institute (termed PMC cohort). Briefly, we first defined genes that distinguish SCCOHT tumors from other pediatric tumors by obtaining variance stabilizing transformation (VST) values from raw count RNA-seq data of SCCOHT tumor tissues (*n* = 5) and other pediatric tumor tissues (*n* = 19) using R package DESeq2 version 1.40.2 and selecting top 2500 genes based on SD of the VST values (*25*). Then, VST values of these genes from RNA-seq data of the tumor tissues (*n* = 24) and SCCOHT tumoroids (*n* = 8) were used to measure the Pearson correlation between samples.

Next, the transcriptomic profiles of SCCOHT tumoroids were compared with those of tumors in the PMC, Curie, and Gustave Roussy cohorts (*24*). Briefly, RNA-seq raw counts of SCCOHT (*n* = 11), ATRT (*n* = 38), ECRT-SMARCA4 (*n* = 4), and ECRT-SMARCB1 (*n* = 19) tumors were obtained from Gene Expression Omnibus (GEO) database (GEO accession: GSE175891). Y chromosome genes were removed as previously described (*24*). Metadata including tumor type and data origin was retrieved from supplementary data table S1 of (*24*). Shared genes (*n* = 25,428) between multiple cohorts were selected for downstream analysis. To remove batch effects of RNA-seq data between different cohorts, all samples (*n* = 104) were first analyzed using DESeq2, and “tumor type” (15 tumor types), “data origin” (three cohorts), and “sample type” (“tumor” versus “tumoroid”) variables were modeled into a design. Then, limma:removeBatchEffect() (*25*) function was used to remove count variances associated with data origin, while count variances associated with biological covariates including tumor type and sample type variables were kept. Resulting VST values were used to perform UMAP analysis. The VST value of selected genes (*EZH2*, *CCND1*, *KDM6A*, and *KDM6B*) per sample was visualized using ggplot.

R package fgsea version 1.26.0 was used to perform GSEA using hallmark gene sets, chemical and genetic perturbations, and SMARCA4 target gene sets which were retrieved from Molecular Signatures Database (MsigDB) (v2023.1.Hs) (*72*–*75*). R package pheatmap version 1.0.12 or Morpheus (https://software.broadinstitute.org/morpheus) was used to generate heatmaps. R package EnhancedVolcano version 1.18.0 was used to generate a volcano plot.

### Polymerase chain reaction

Primers used in this study are listed in table S6. iQ SYBR Green Supermix (Bio-Rad, Hercules, CA, USA) was used for quantitative PCR experiments. RT-qPCR results were obtained from three biological replicates.

### Mutational signature analysis

R package MutationalPatterns version 3.10.0 (*76*) was used to extract de novo mutational signatures from 96 mutation profiles of each SCCOHT tumor and tumoroid. Five extracted signatures were merged into three signatures, based on their cosine similarities (≥0.85), which were then substituted by reference single-base substitution signatures in Signal based on their cosine similarities (≥0.85) (*22*).

### Compound library

The in-house pediatric cancer library versions L11 and L13 contain 198 and 224 drugs, respectively. Almost all drugs are dissolved in dimethyl sulfoxide (DMSO) and stored at room temperature under nitrogen atmosphere. Five drugs (metformin, perifosine, carboplatin, oxaliplatin, and copanlisib) are dissolved in MQ. and one (cisplatin) is dissolved in a saline solution, which are stored at −20°C. Before medium-throughput screening, the 384-well working plates (384LDV-Plates, Labcyte, San Jose, CA, USA) containing the dissolved drugs are shaken (30 min at room temperature) and centrifuged (2:30 min at 1500 rpm). Subsequently, the working plates are surveyed with the Echo550 dispenser to determine whether the amount of solution in the wells is sufficient to start the screen (minimal 2.5 μl), and the DMSO percentage is >80%.

### Medium-throughput drug screening

Medium-throughput drug screening was performed at the High-throughput screening (HTS) facility of the PMC for Pediatric Oncology, the Netherlands. Tumoroids were dissociated into single cells using TrypLE. Forty microliters of cells at a cell density of 750 to 2000 cells per well was plated in a flat-bottom 384-well tissue culture–treated microplate (#3764, Corning, NY, USA) using a multidrop combi reagent dispenser (Thermo Fisher Scientific). Cells were cultured for 3 days under standard culturing conditions (5% CO_2_, 37°C). Next, the Cell Titer Glo 3D (CTG3D, G9683, Promega, Madison, WI, USA) cell viability assay was performed for the *t* = 0 microplate using the supplier’s protocol by measuring luminescence. Subsequently, the drugs were added to tissue culture microplates and the library using the high-throughput screening facility. Using the Echo550 dispenser, 100 nl of the drugs (in DMSO or MQ, at different concentrations) was added to the wells containing the cells, to yield final concentrations of 0.1, 1 10, and 100 nM and 1 and 10 μM (0.25% DMSO or MQ). Cells treated with only DMSO were used as positive controls, whereas cells treated with staurosporine (final concentration of 10 μM) were used as negative controls. The cells were incubated with the compounds for 5 days at standard culturing conditions. Next, the CTG3D assay was performed for the microplates. These medium-throughput screens were performed in technical duplicates.

The data were normalized to the DMSO-treated cells (defined as 100% viability) and the empty controls (0% viability). The corresponding values were used to plot the dose-response curves, and several values were determined from the dose-response curves using the extension package drc in the statistic environment of R Studio (version 4.0.2) (*77*): the definite integral of the curve (AUC) and the cell growth of the DMSO-treated cells during the screen by dividing the CTG3D signal of the DMSO vehicle controls at day 5 by the signal at *t*0 (cell growth). Cell grow rate data were available in 32 tumoroids. Quality of the screens was approved after assessment of the cell growth, the negative, positive, and empty controls, and the amount of variability between the duplicates.

To identify small-molecule inhibitors that are selective against SCCOHTs, the drug responses at day 5 in 8 SCCOHT tumoroids were compared with drug responses at day 5 in 25 tumoroids of different cancer types which were screened at our institute (*26*–*29*). Normalized AUC values were calculated as previously described (*78*). R package pheatmap version 1.0.12 was used to generate heatmaps. Built-in functions prcomp and R package ggplot2 version 3.4.4 were used to generate a principal components analysis plot. The VST value of selected genes (*EZH2*, *CCND1*, *KDM6A*, and *KDM6B*) and the *z*AUC value of selected drugs (tazemetostat, palbociclib, and GSK J4) in SCCOHT tumoroids were visualized using ggplot.

### Validation drug screen

Validation screens using increasing concentrations of methotrexate (Selleckchem, TX, USA) were done in 96-well plates (cell density of 2000 to 5000 cells per well) following the above-mentioned workflow. Folic acid (100 μM; Sigma-Aldrich), 50 μM tetrahydrofolic acid (Santa Cruz, TX, USA), 1 mM betaine (Sigma-Aldrich), 1 mM l-methionine (Sigma-Aldrich), 5 μM istradefylline (Selleckchem), 75 μM caffeine (Sigma-Aldrich), 50 μM thymidine (Sigma-Aldrich) and 250 μM hypoxanthine (Sigma-Aldrich) were used in combination with DMSO control or 30 nM methotrexate (Selleckchem). These screens were performed in technical triplicates and biological duplicates or triplicates as indicated.

### Methotrexate efficacy in SCCOHT tumoroids in 3D or 2D

3D tumoroids were collected from two representative lines (PS-01a-O and PS-02a-O). An equal volume of cell suspension was used to embed 3D tumoroids in pure (>80%) Matrigel domes (10 μl per well) or to digest into single cells and seed in 2D (~10,000 cells per well) on 96-well plates. The tumoroids were incubated overnight before being treated with increasing concentrations of methotrexate. After 5 days, the tumoroids were imaged using a bright-field microscope, and cell viability was measured using CTG3D according to the manufacturer’s instructions.

### Live-cell imaging

Tumoroids were seeded in black optically clear-bottom 96-well plates (PerkinElmer, Waltham, MA, USA) at a cell density of approximately 200 cells per well. After 3 days, cells were treated with the indicated concentrations of methotrexate. Medium containing methotrexate was replenished twice a week for 2 weeks. Then, live cells were stained with CellTracker green-fluorescent chloromethyl derivatives of fluorescein diacetate (Green CMFDA) Dye (Invitrogen) following the manufacturer’s instructions, and the whole plate was imaged using a Leica DMi8 Live cell microscope (×5 magnification). This experiment was performed in technical singlicate and biological triplicates. Fiji (*79*) was used to measure the integrated density value in each well. These values were normalized to the DMSO vehicle control.

### Statistical analysis

The Student *t* test was used to test statistical difference between log normalized FPKM values of *SMARCA4* and *SMARCA2* in SCCOHT tumors and tumoroids versus other tumor tissues and between IC_50_ values of methotrexate in SCCOHT tumoroids treated with or without folic acid. The multiple *t* test was used to test statistical difference between normalized AUC values of each drug in SCCOHT tumoroids and other tumoroids. The Mann-Whitney test was used to test statistical difference between cell growth rates of SCCOHT tumoroids and other tumoroids. The One-way analysis of variance (ANOVA) was used to test statistical significance of quantified CMFDA signal intensity changes in SCCOHT tumoroids with or without methotrexate treatment. Bar graph data are presented as the mean ± SEM unless indicated otherwise.
