# Supplementary material for: Organoid drug profiling identifies methotrexate as a therapy for SCCOHT, a rare pediatric cancer
Source: Sci Adv. 2025 Feb 26;11(9):eadq1724. doi: 10.1126/sciadv.adq1724 (PMC11864178; doi:10.1126/sciadv.adq1724)
Supplement: Supplementary file 1 — Figs. S1 to S8 Tables S1 to S6 [file sciadv.adq1724_sm.pdf]

Science Advances
AAAS

Supplementary Materials for

Organoid drug profiling identifies methotrexate as a therapy for SCCOHT, a rare pediatric cancer

Seok-Young Kim *et al.*

Corresponding author: Hans Clevers, h.clevers@hubrecht.eu

Sci. Adv. **11**, eadq1724 (2025)
DOI: 10.1126/sciadv.adq1724

This PDF file includes:

Figs. S1 to S8
Tables S1 to S6

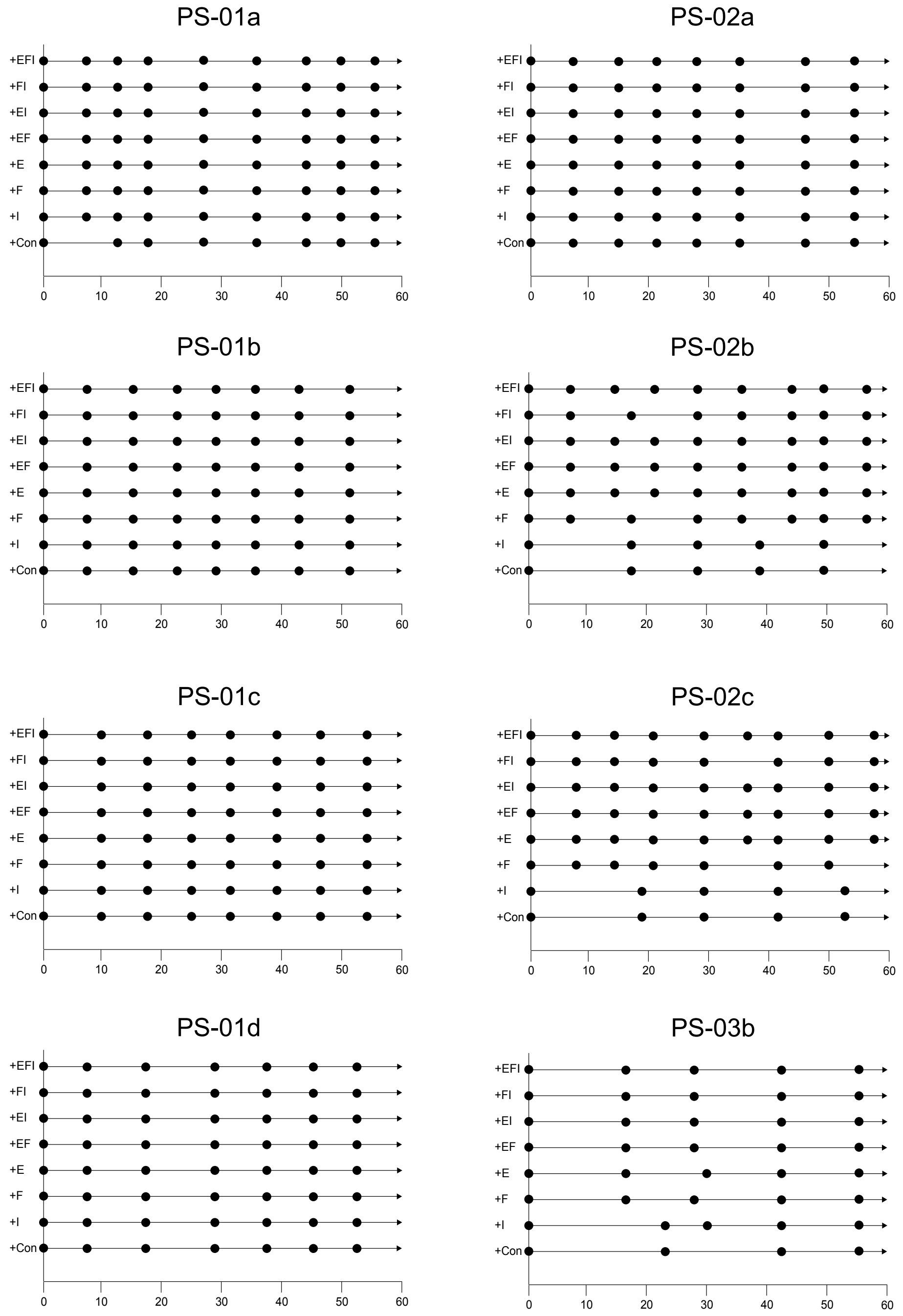

Fig. S1. Growth factor requirements of SCCOHT tumoroids. Tumoroids were cultured in the indicated combination of growth factors upto 60 days. Each dot indicates 1 passaging. Arrow indicates tumor cells were viable at the end of the experiments. Representative results from 2 biological replciates are shown. E, EGF; F, FGF2; I, IGF1.

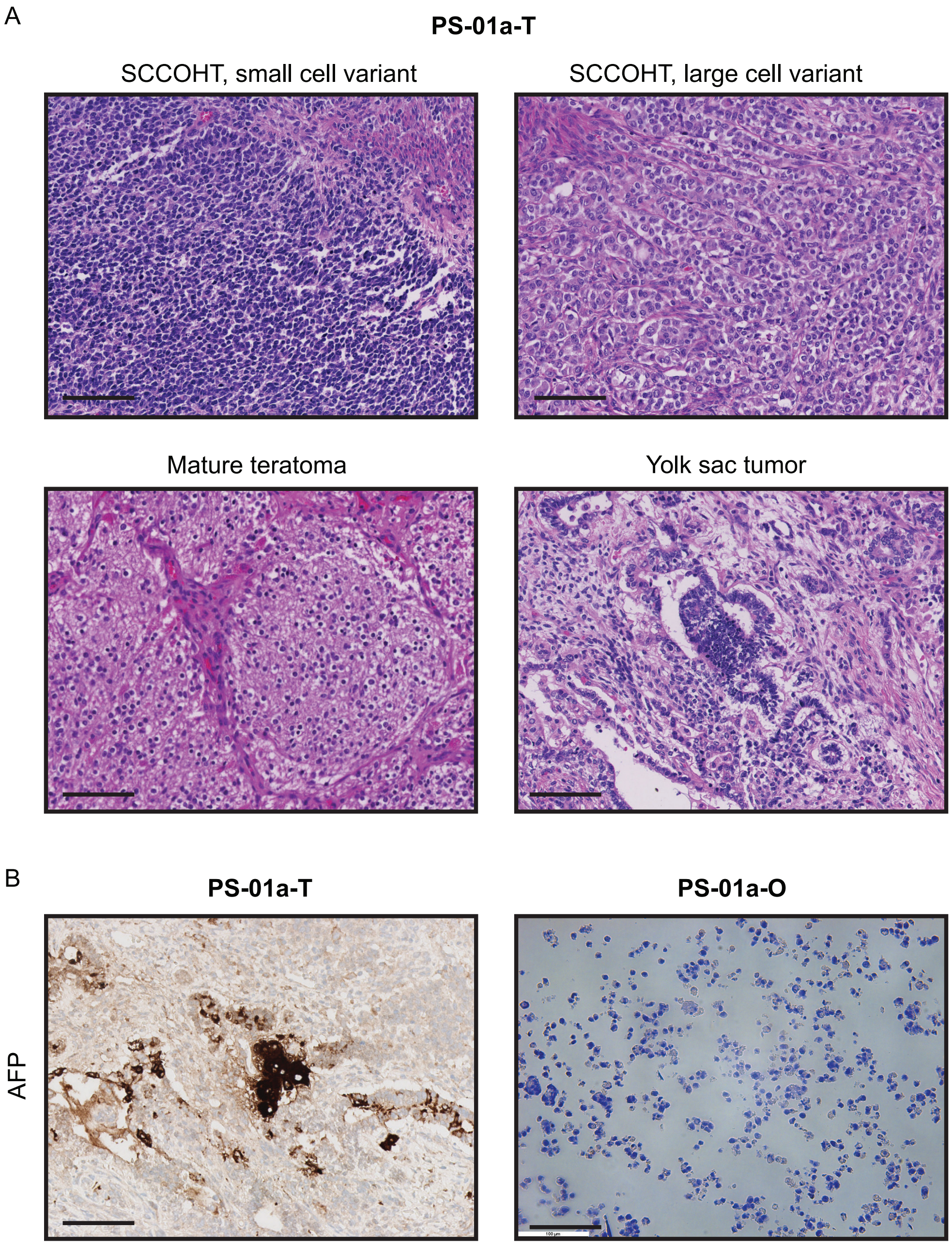

Fig. S2. Small cell and large cell variant of SCCOHT, mature teratoma (glial tissue), and yolk sac tumor in PS-01a-T specimen. (A) H&E images of PS-01a-T illustrating presence of different components in PS-01a-T specimen. (B) Immunohistochemistry images of PS-01a-T and PS-01a-O stained with AFP, a marker for yolk sac tumor.

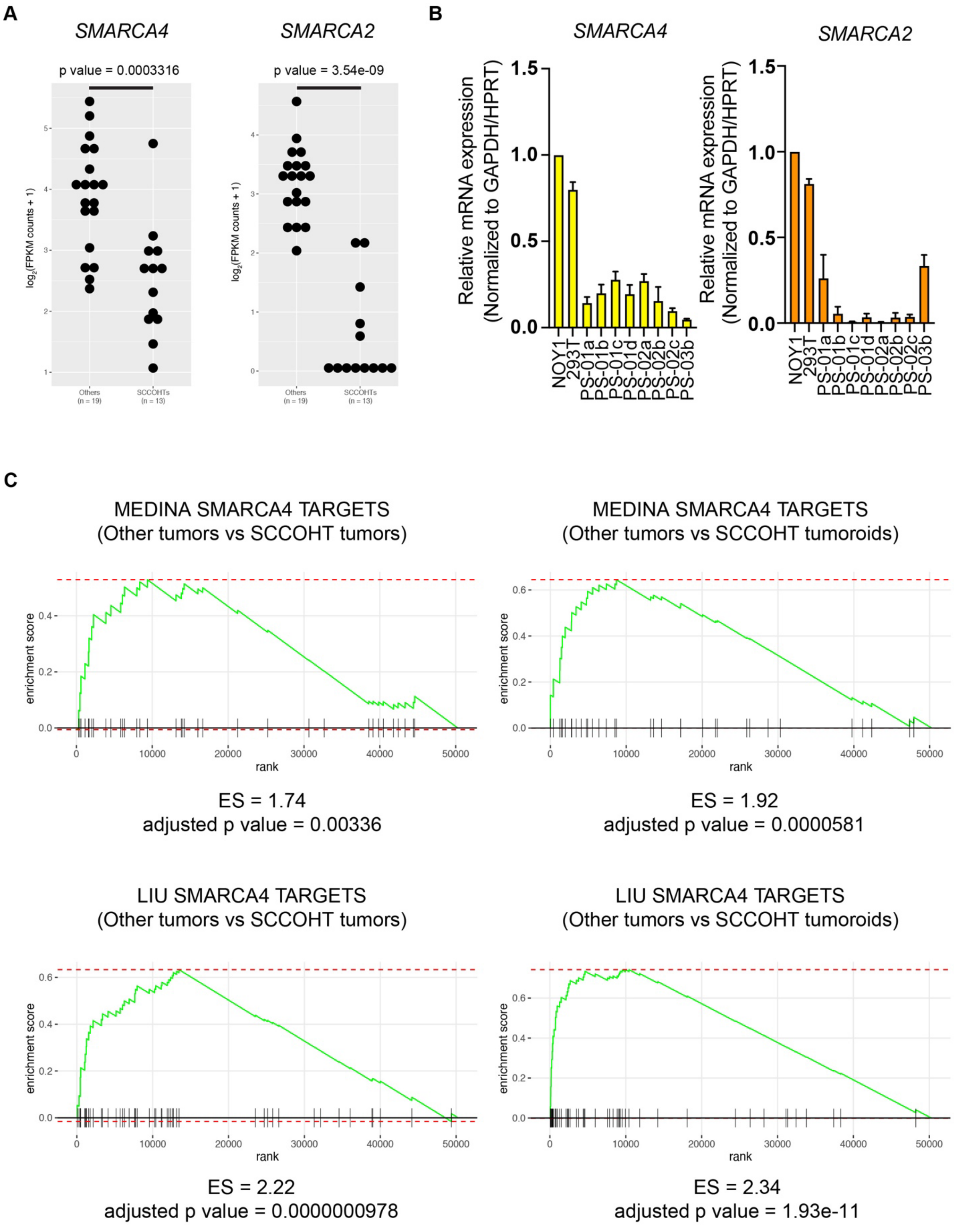

Fig. S3. *SMARCA4* and *SMARCA2* mRNA expression and gene-set enrichment analysis using SMARCA4 target genes. (A) A dotplot showing log-normalized FPKM counts of

SMARCA4 (left) and *SMARCA2* (right) in other pediatric tumors (n = 19), SCCOHT tumors and tumoroids (n = 13). Each dot indicates an individual sample (two-tailed Student t-test). (B) Quantitative PCR results showing *SMARCA4* and *SMARCA2* mRNA expression in SMARCA4 wild-type cell lines (NOY1 and 293T) and SCCOHT tumoroids. (C) Gene-set enrichment analyses using MEDINA SMARCA4 TARGETS and LIU SMARCA4 TARGETS gene sets in other tumors (n = 19) vs SCCOHT tumors (n = 5) and other tumors (n = 19) vs SCCOHT tumoroids (n = 8). Enrichment score (ES) and adjusted p value are shown below each analysis.

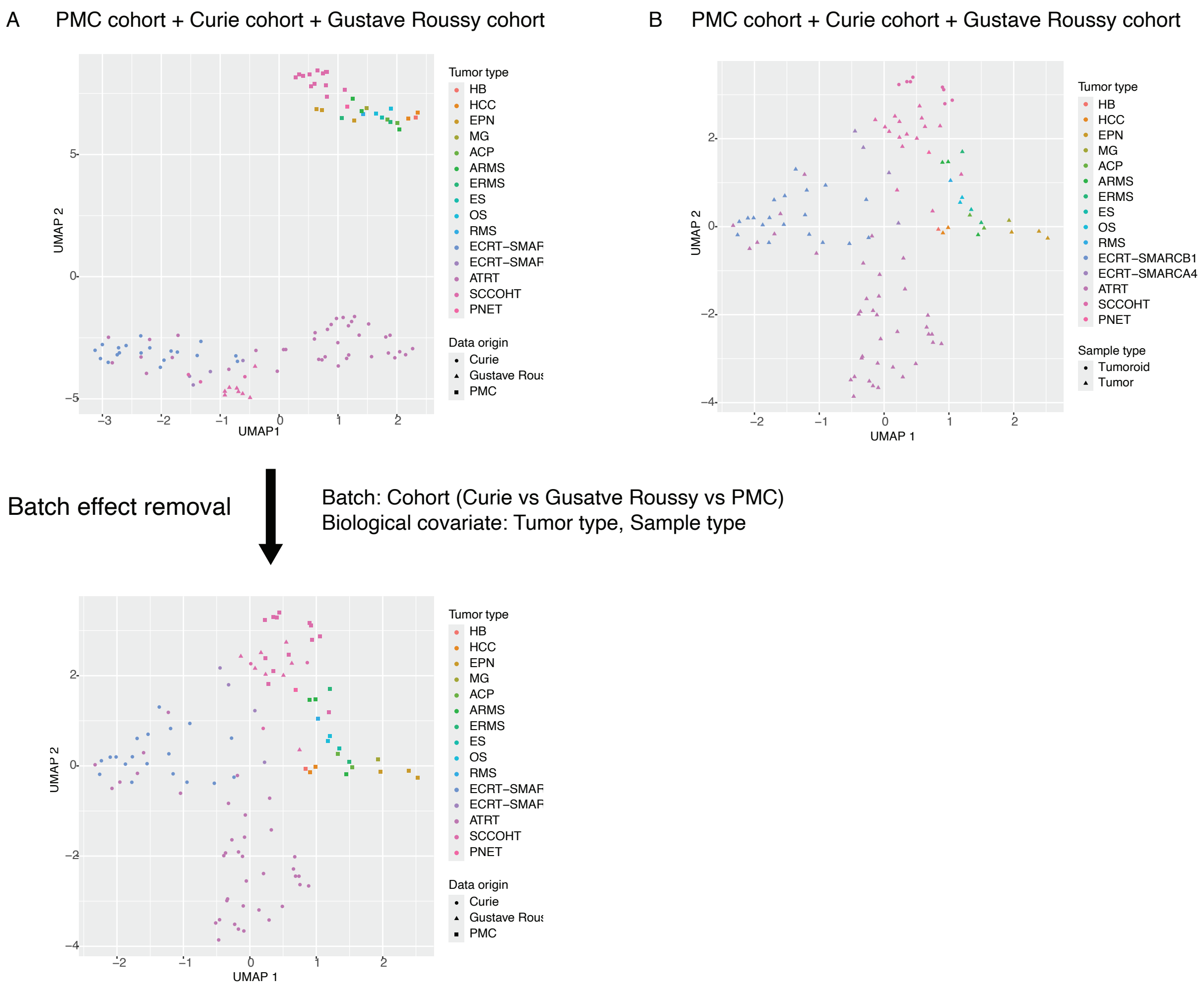

Fig. S4. UMAP analyses of SCCOHT tumors (n = 16), tumoroids (n = 8), SWI/SNF-deficient rhabdoid tumors (n = 61), and other various pediatric tumors (n = 19) in Princess Maxima Center (PMC), Curie, and Gustave Roussy cohorts based on RNA-sequencing data before and after batch effect removal. (A) Each dot indicates an individual sample. Tumor type is color-coded. Count variances associated with cohort, or data origin, was regressed out while count variances associated with tumor type and sample type were not regressed out. SCCOHT tumors from Curie (circle), Gusatve Roussy (triangle), and PMC (square) are grouped after batch effect removal. HB, hepatoblastoma; HCC, hepatocellular carcinoma; EPN, ependymoma; MG, malignant glioma; PNET, primitive neuroectodermal tumor; ARMS, alveolar rhabdomyosarcoma; ERMS, embryonal rhabdomyosarcoma; RMS, rhabdomyosarcoma; OS, osteosarcoma; ES, ewing sarcoma; ACP, adamantinomatous craniopharyngioma; ATRT, atypical teratoid/rhabdoid tumor; ECRT-SMARCB1, SMARCB1-deficient extracranial rhabdoid tumor; ECRT-SMARCA4, SMARCA4-deficient extracranial rhabdoid tumor. (B) UMAP analysis after batch effect removal. Sample type is annotated by shapes. SCCOHT tumoroid (circle) are grouped together with SCCOHT tumors (triangle).

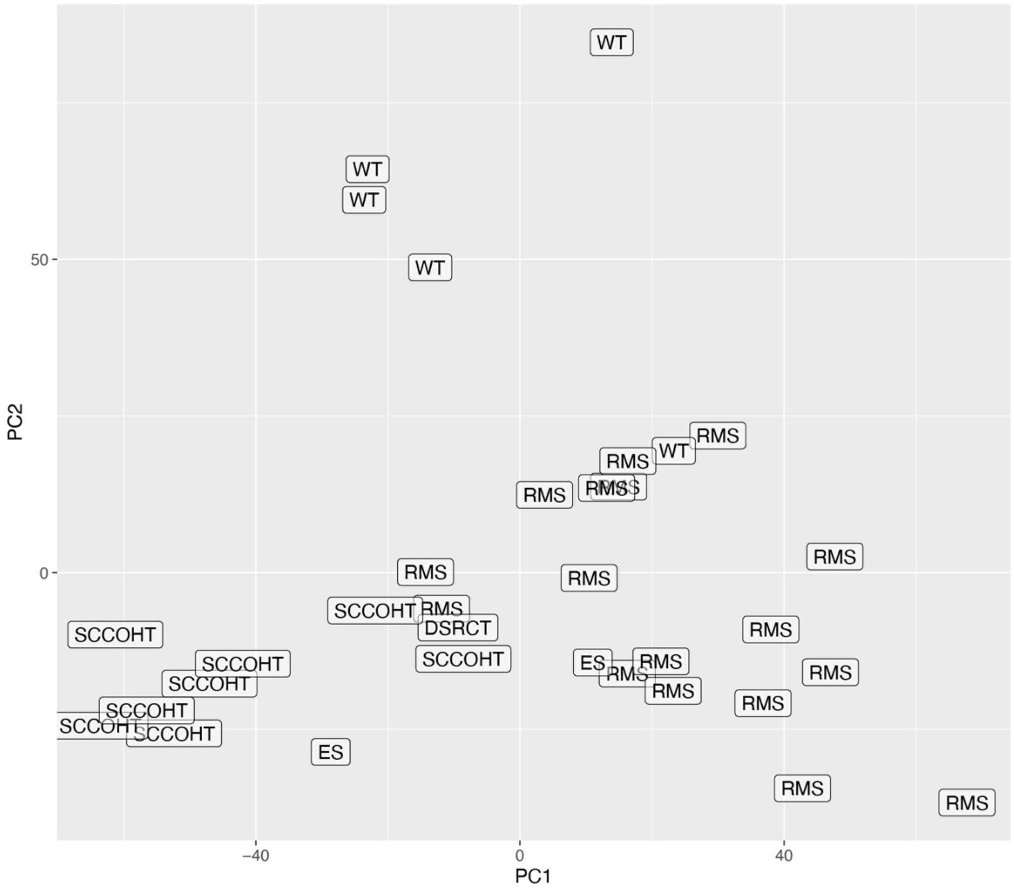

Fig. S5. Principal component analysis (PCA) of normalized AUC values of 153 drugs in 33 tumoroids. Distinct groups of SCCOHT, rhabdomyosarcoma (RMS), and wilms tumor (WT) were identified.

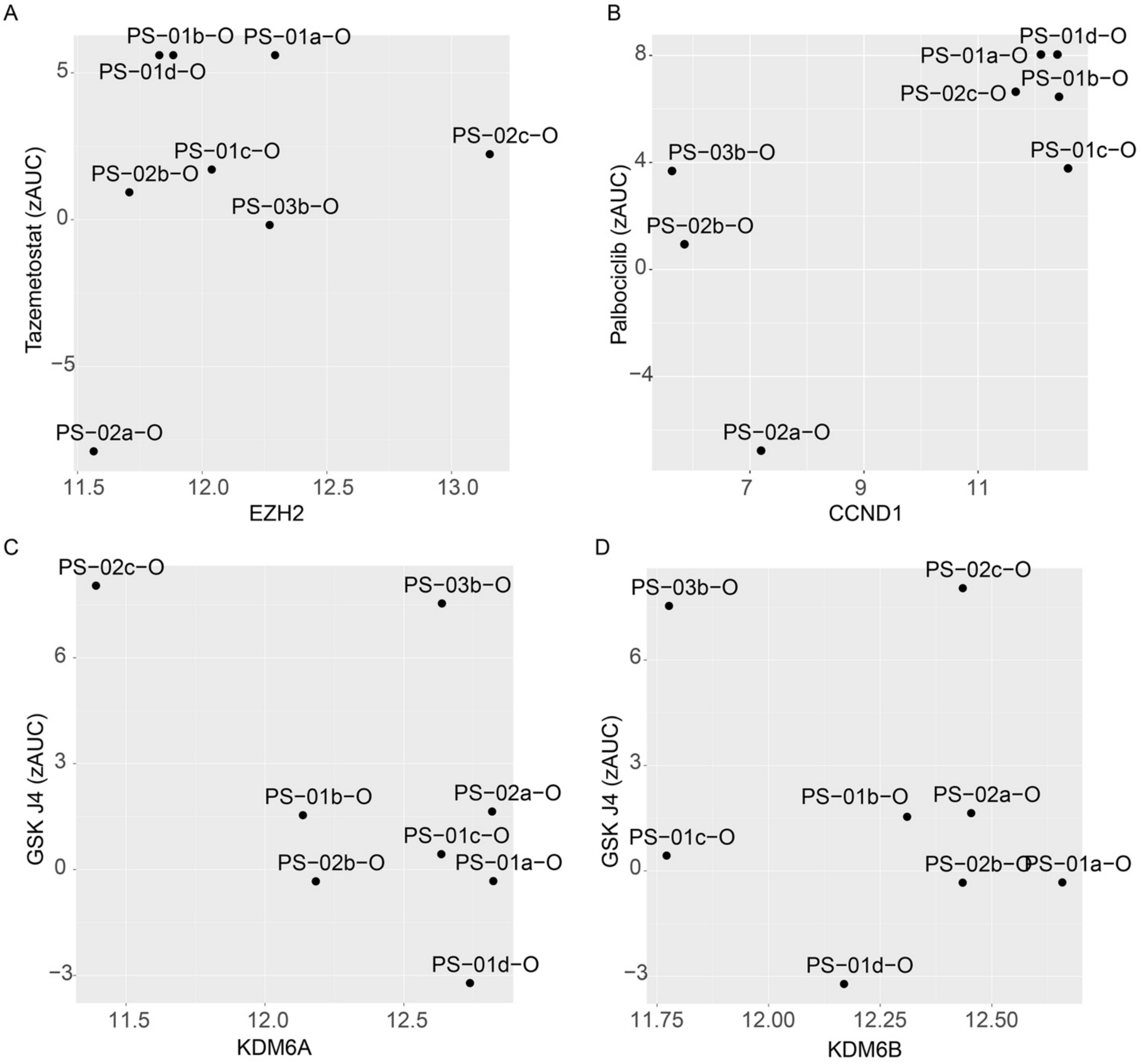

Fig. S6. Scatter plots showing zAUC values of selected drugs in Fig. 4C and their proposed biomarkers. (A) A plot showing *EZH2* expression and tazemotostat response in each SCCOHT tumoroid. (B) A plot showing *CCND1* expression and palbociclib response in each SCCOHT tumoroid. (C and D) Plots showing *KDM6A* (C) or *KDM6B* (D) expression and GSK J4 response in each SCCOHT tumoroid.

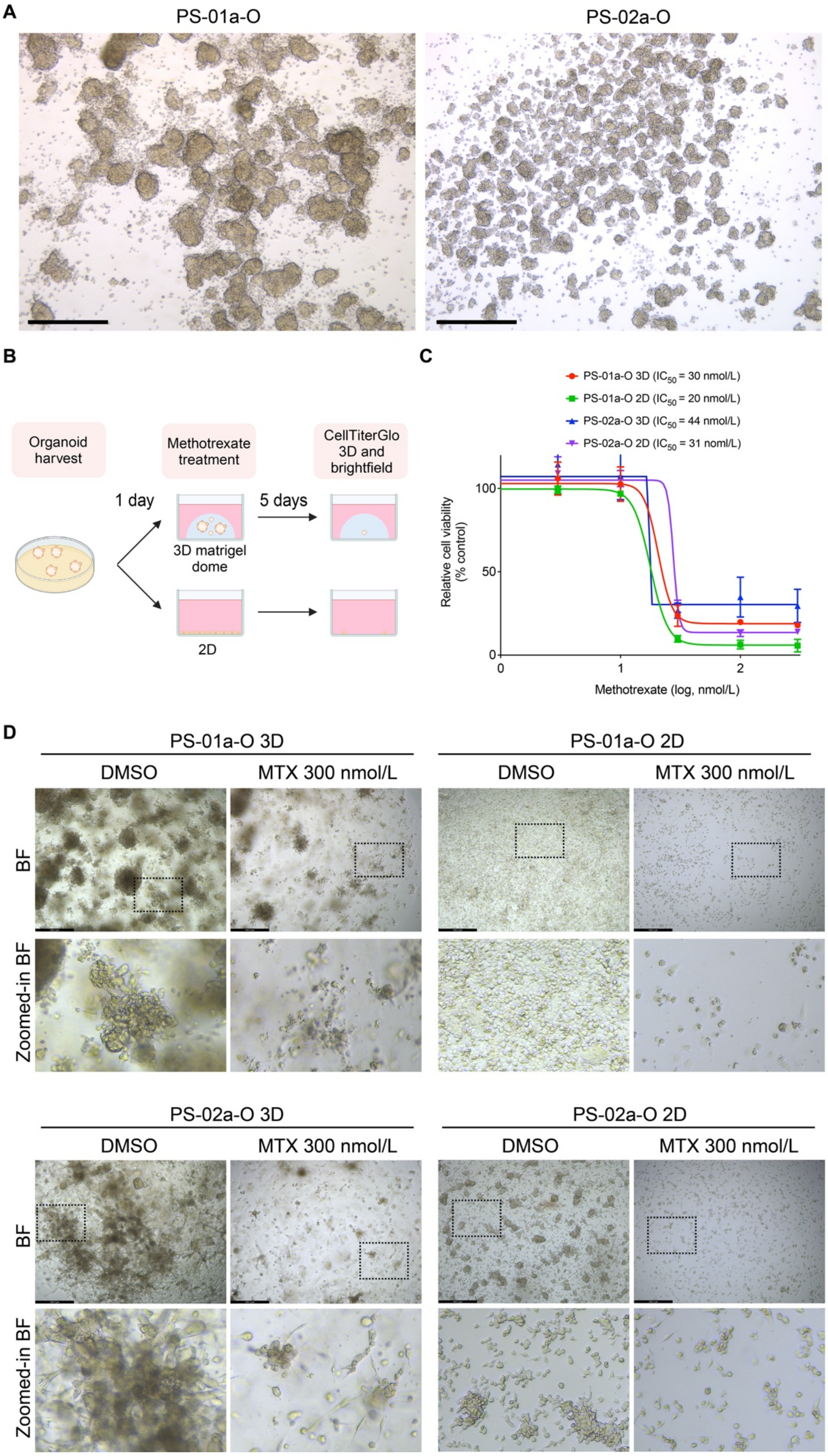

A
PS-01a-O
PS-02a-O
B
Organoid harvest
Methotrexate treatment
CellTiterGlo 3D and brightfield
1 day
5 days
3D matrigel dome
2D
C
PS-01a-O 3D (IC50 = 30 nmol/L)
PS-01a-O 2D (IC50 = 20 nmol/L)
PS-02a-O 3D (IC50 = 44 nmol/L)
PS-02a-O 2D (IC50 = 31 noml/L)
Relative cell viability (% control)
Methotrexate (log, nmol/L)
D
PS-01a-O 3D
PS-01a-O 2D
DMSO
MTX 300 nmol/L
BF
Zoomed-in BF
PS-02a-O 3D
PS-02a-O 2D

Fig.S7. Methotrexate efficacy in 3D or 2D grown SCCOHT tumoroids. A) Bright field images of representative SCCOHT tumoroids (PS-01a-O, PS-02a-O). SCCOHT tumoroids are grown as a mixture of 3D and 2D tumoroids. Scale bar, 243.5 μm. B) An experimental scheme to test the effect of dimensionality (3D vs 2D) on methotrexate efficacy in representative SCCOHT tumoroids (PS-01a-O, PS-02a-O). An equal volume of cell suspension was seeded in 3D matrigel domes or in 2D. After 1 day, cells were treated with increasing concentrations of methotrexate. After 5 days, cell viability was measured using CellTiterGlo 3D and cell morphologies were imaged using a bright field microscope. C) Cell viability curves of PS-01a-O in 3D (red) or 2D (green) and PS-02a-O in 3D (blue) or 2D (purple) treated with increasing concentrations of methotrexate. The IC50 value of each condition per line is indicated above. Results from biological duplicates are shown. D) Representative bright field images (BF) and zoomed-in images (Zoomed-in BF) of indicated regions (dashed line) of PS-01a-O 3D, 2D and PS-02a-O 3D, 2D with or without methotrexate (MTX) treatment. Scale bar, 243.5 μm.

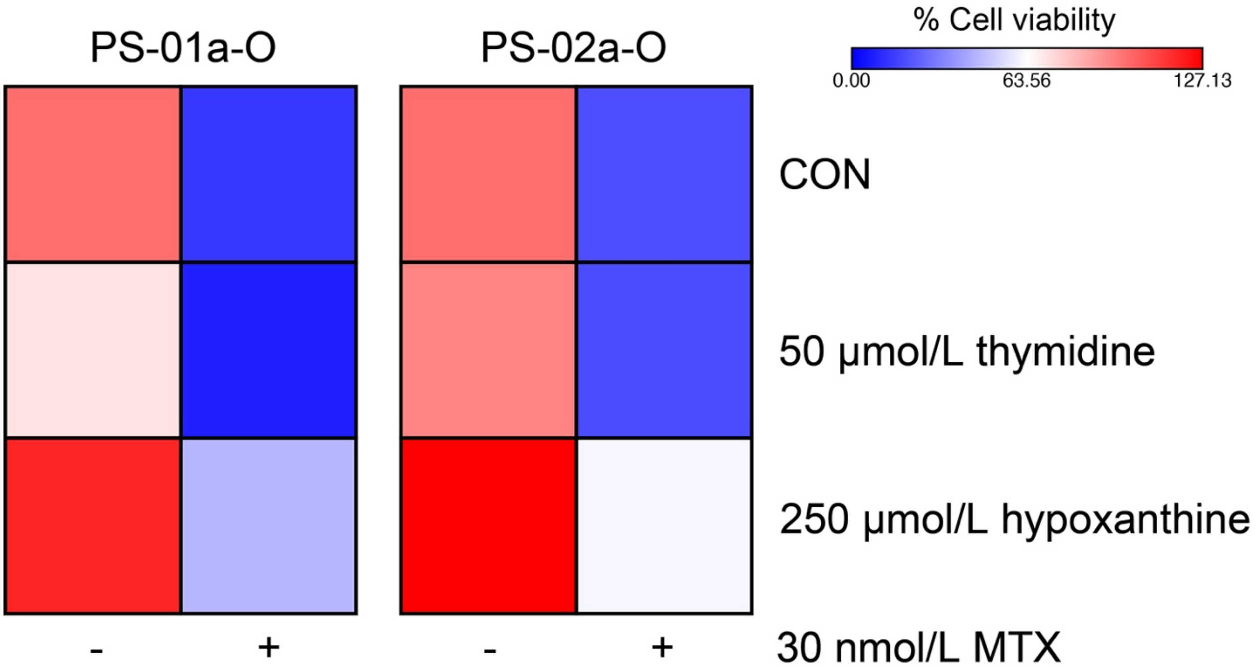

Fig.S8. Bar graph showing cell viability of SCCOHT tumoroids (PS-01a-O, PS-02a-O) treated with thymidine and hypoxanthine in combination with DMSO control or 30 nmol/L MTX. Results from biological duplicates are shown.

Table S1. Multiple t-test results comparing drug responses in 8 SCCOHT tumoroids between drug responses in 25 tumoroids of other pediatric or AYA cancers.

Drug name	Mean Z score of 8 SCCOHT tumoroids	Mean Z score of 25 other tumoroids	Difference in mean values	SE of difference	Adjusted P Value
6-Mercaptopurine	-3.604	1.547	-5.15	1.55	0.25002
6-Thioguanine	-2.633	1.849	-4.482	1.718	0.732446
Abemaciclib	2.623	0.9662	1.657	2.245	0.999994
Actinomycin D	-0.5934	1.573	-2.166	1.402	0.999284
Adavosertib	5.832	-0.4427	6.275	1.758	0.184163
Afatinib	-0.04629	2.331	-2.377	2.259	0.999972
Alectinib	2.303	0.3911	1.912	1.88	0.999972
Alisertib	4.323	0.1323	4.191	1.93	0.950535
AMG 337	3.137	0.2464	2.89	1.172	0.759043
Apatinib	4.253	0.9348	3.318	0.9577	0.146709
AT7519	6.972	-0.8003	7.772	1.383	0.002639
Axitinib	8.16	-0.8047	8.964	1.29	0.000018
AZD4547	6.161	-2.279	8.439	1.122	0.000002
AZD5582	3.933	0.006573	3.927	1.14	0.150921
AZD8055	8.026	-1.895	9.921	2.059	0.087815
Berzosertib	6.378	-1.061	7.439	1.828	0.146709
Binimetinib	4.329	0.41	3.919	1.121	0.141205
Birabresib	5.827	-1.11	6.937	1.498	0.014117
Birinapant	4.615	0.06028	4.555	1.319	0.16602
Bortezomib	5.198	0.5135	4.685	1.188	0.04364
Buparlisib	7.195	-0.2365	7.431	1.219	0.000097
Busulfan	2.272	0.0927	2.179	1.916	0.99996
Cabozantinib	1.98	0.2677	1.713	1.239	0.99974
Camptothecin	-1.588	1.364	-2.952	1.793	0.998926
Capmatinib	4.12	1.158	2.962	0.9948	0.382505
Cediranib	2.868	-1.223	4.091	1.31	0.288351
Ceralasertib	8.513	-0.7836	9.297	1.18	0.000007
Ceritinib	5.439	-2.113	7.552	1.47	0.002317
Cobimetinib	5.464	-0.3758	5.84	1.381	0.027542
CPI-203	8.164	-1.279	9.443	1.365	0.000205
CPI-455	3.311	-0.6141	3.925	1.22	0.267758
Crenigacestat	2.406	-0.09297	2.499	1.343	0.988064
Crenolanib	2.097	0.6132	1.484	1.796	0.999994

Crizotinib	3.676	-0.253	3.929	1.671	0.877337
Cytarabine	3.967	0.223	3.744	1.761	0.944364
Dabrafenib	3.242	0.4322	2.809	1.225	0.862498
Dasatinib	3.736	0.2151	3.521	2.015	0.998513
Daunorubicin	1.898	1.658	0.2399	1.263	>0.999999
Decitabine (Guadecitabine)	7.328	-0.7392	8.067	1.141	0.000005
Defactinib	4.57	-0.1713	4.741	1.231	0.055522
Dexamethasone	2.62	0.08448	2.536	1.368	0.988064
Dovitinib	2.837	-1.017	3.854	1.52	0.723217
Doxorubicin	2.661	1.3	1.361	1.154	0.999956
Eganelisib	7.525	-1.467	8.992	1.387	0.000196
EHT 1864	3.685	-0.3494	4.034	1.366	0.3926
Ensartinib	4.853	-1.559	6.412	2.751	0.94777
Entinostat	6.458	-0.8611	7.319	1.622	0.034063
Entospletinib	1.629	-0.5315	2.161	2.159	0.999972
Entrectinib	5.521	-1.38	6.901	1.73	0.105649
Epidaza	5.668	-0.4756	6.143	1.36	0.018338
Erlotinib	1.231	1.184	0.04697	1.585	>0.999999
Etoposide	3.359	0.9088	2.45	1.692	0.999722
Everolimus	-1.904	1.524	-3.428	1.351	0.718818
Fenretinide	4.721	0.3633	4.358	1.337	0.245248
Fimepinostat	2.933	0.3369	2.596	1.737	0.999722
Galunisertib	3.663	-0.05869	3.722	1.202	0.314961
Ganetespib	3.519	0.8254	2.694	1.941	0.999842
Gemcitabine	1.041	-0.1582	1.199	2.24	>0.999999
Glasdegib	4.474	-0.9511	5.425	1.118	0.005139
GSK J4	1.916	-0.6282	2.544	1.865	0.999804
GSK1070916	4.913	-0.26	5.172	1.595	0.371111
GSK2636771	2.583	-0.1356	2.719	1.89	0.99974
GSK269962A	3.534	0.06219	3.471	1.595	0.92683
I-BRD9	4.327	-0.3037	4.631	1.661	0.696173
Ibrutinib	1.237	2.114	-0.8769	2.384	>0.999999
Icotinib	3.735	0.8839	2.851	1.186	0.794727
Imatinib	2.801	0.8644	1.936	1.377	0.99974
Ipatasertib	7.982	-2.216	10.2	1.084	<0.000001
Isotretinoin	3.645	-0.7436	4.388	1.291	0.16602
KU-55933	1.683	-0.5474	2.23	1.573	0.99974
KU-60019	0.6864	1.186	-0.4999	2.042	>0.999999
Lapatinib	0.008916	0.9168	-0.9078	2.641	>0.999999

Larotrectinib	3.176	-0.4852	3.661	1.394	0.65016
Lenvatinib	5.546	-1.423	6.968	1.479	0.011193
LGK974	4.335	-0.3266	4.662	1.2	0.050145
Linsitinib	6.714	-2.775	9.489	0.9882	<0.000001
LMK-235	6.071	-0.6865	6.757	1.828	0.202072
Lorlatinib	4.924	-0.4085	5.333	1.322	0.03553
LTURM34	3.837	-0.4987	4.336	2.015	0.958652
Masitinib	5.098	-0.5455	5.644	1.353	0.034063
Melphalan	4.458	-0.7972	5.255	1.573	0.24521
Merestinib	4.304	-0.5285	4.832	1.646	0.51207
Methotrexate	-6.318	2.578	-8.895	1.274	0.000032
Miransertib	7.522	-2.482	10	1.324	0.000007
Mirdametinib	4.444	0.2182	4.225	1.206	0.134079
Mitoxantrone	-0.6146	1.628	-2.243	1.393	0.998926
MK-2206	8.328	-1.906	10.23	1.119	<0.000001
MM102	3.527	-0.6367	4.163	1.252	0.210669
Molibresib	6.787	-0.52	7.307	1.215	0.000135
Momelotinib	4.953	-0.672	5.625	1.782	0.417263
Mubritinib	-4.417	-0.335	-4.082	2.386	0.998926
MX69	3.942	-1.137	5.08	1.177	0.021617
Navitoclax	6.646	-3.023	9.67	1.498	0.001207
Neratinib	0.5149	0.2019	0.313	2.549	>0.999999
Nilotinib	0.9412	0.404	0.5372	1.775	>0.999999
Niraparib	6.344	0.2285	6.116	1.6	0.133614
Olaparib	5.897	-0.3627	6.259	1.263	0.003308
Paclitaxel	-2.455	1.002	-3.457	1.43	0.794917
Palbociclib	3.851	0.8241	3.026	1.9	0.999484
Panobinostat	3.99	-0.3645	4.355	1.609	0.696057
Pazopanib	3.515	-1.99	5.506	1.495	0.087815
PCI-34051	3.787	-1.035	4.823	1.264	0.061874
Perifosine	3.074	-0.1759	3.25	1.33	0.772931
Pevonedistat	3.486	-0.0008777	3.487	2.067	0.998926
Pictilisib	5.68	-1.209	6.889	2.214	0.579722
Pinometostat	1.22	-0.3457	1.566	1.591	0.999972
Ponatinib	5.568	-1.841	7.409	1.534	0.008785
Prednisolone	1.908	0.5386	1.369	1.746	0.999994
Prexasertib	5.329	-0.2998	5.629	1.855	0.511353
Quizartinib	2.741	0.6739	2.068	1.675	0.99995
Ravoxertinib	4.416	-0.01753	4.433	1.353	0.223293

Regorafenib	0.8608	0.227	0.6338	1.674	>0.999999
Ribociclib	3.249	1.64	1.609	1.917	0.999994
Ritlectinib	2.201	-0.4842	2.685	1.699	0.999265
Romidepsin	1.591	0.02817	1.562	1.419	0.99996
Ruxolitinib	3.44	-0.4413	3.881	1.222	0.273977
S63845	0.008965	1.014	-1.005	2.536	>0.999999
Samotolisib	6.008	-2.189	8.197	2.31	0.382505
Sapitinib	0.7933	2.051	-1.258	1.329	0.999972
SAR405	3.134	0.8577	2.276	1.807	0.99995
Saracatinib	-3.514	1.115	-4.629	3.947	0.99996
Savolitinib	4.837	-0.5633	5.4	1.221	0.015844
Selinexor	3.929	-1.117	5.046	1.932	0.772931
Selumetinib	4.504	0.05583	4.448	1.097	0.035997
Sepantronium bromide	-1.08	1.907	-2.987	2.538	0.99996
Sirolimus	6.526	-1.342	7.868	2.256	0.417263
Sonidegib	2.271	0.479	1.792	1.503	0.999956
Sorafenib	-1.615	-0.2452	-1.37	1.788	0.999994
Sunitinib	5.437	-1.065	6.501	1.567	0.063749
Talazoparib	3.063	0.377	2.686	2.101	0.999941
Tanespimycin	-1.369	2.024	-3.392	1.647	0.978808
Taselisib	9.12	-2.07	11.19	1.227	0.000002
Tazemetostat	1.7	0.94	0.7603	1.899	>0.999999
Temozolomide	2.806	1.721	1.085	0.9531	0.99996
Temsirolimus	4.382	-1.165	5.547	2.764	0.988947
ThioTEPA	1.627	1.259	0.3679	1.305	>0.999999
Tivantinib	-2.008	1.021	-3.029	1.922	0.999284
Topotecan	2.868	0.8652	2.003	1.652	0.999956
Trametinib	0.4893	0.4156	0.07373	1.746	>0.999999
Vactosertib	3.736	0.1122	3.623	1.39	0.704474
Vandetanib	4.609	-0.336	4.945	2.1	0.920584
Varlitinib	0.6714	0.2562	0.4152	2.299	>0.999999
Vemurafenib	1.427	0.0328	1.395	1.348	0.999967
Venetoclax	2.873	-1.606	4.478	1.421	0.274711
Vinblastine	1.036	0.5916	0.4443	1.579	>0.999999
Vincristine	1.201	0.5412	0.6597	1.54	>0.999999
Vismodegib	2.817	1.054	1.763	1.239	0.999726
Vistusertib	7.806	-1.311	9.116	2.094	0.155334
Volasertib	1.47	0.2079	1.262	1.726	0.999994
Vorinostat	5.278	-1.142	6.421	2.551	0.883246

XAV-939	3.718	0.454	3.264	1.2	0.579722
Xevinapant	3.106	0.5205	2.585	1.093	0.83233
YO-01027	3.269	-0.8184	4.088	1.364	0.371111

Table S2. IC_{50} values (µmol/L) of drugs described in Fig. 4C in each tumoroid.

Drug name	PS-01a-O	PS-01b-O	PS-01c-O	PS-01d-O	PS-02a-O	PS-02b-O	PS-02c-O	PS-03b-O
Methotrexate	0.05644255	0.04262664	0.04115435	0.04481805	0.04762075	0.05350599	0.27018744	0.04393836
Actinomycin D	0.00050472	0.00050987	0.00067148	0.00034454	0.00070155	0.00076635	0.00204965	0.00040463
Busulfan	>10	>10	>10	>10	>10	>10	>10	>10
Doxorubicin	0.03448275	0.02839851	0.02926176	0.03200953	0.02655044	0.08045654	0.07068022	0.03145543
Etoposide	0.23992596	0.18994327	0.31116183	0.24431902	0.29222202	1.01879628	1.49546661	0.53149732
Melphalan	6.04935726	3.15054713	5.32946555	5.10745167	1.25485832	1.87183252	7.43594119	8.64160972
Paclitaxel	0.00755952	0.0038949	0.00509731	0.00789294	0.00944368	0.00553515	0.01410512	0.00517097
ThioTEPA	3.26200281	2.06898646	2.80552532	2.61194274	1.01132824	4.53023784	8.92661905	1.93625032
Topotecan	0.0473998	0.02887903	0.04869843	0.03491941	0.0392305	0.19691374	0.23915349	0.11721619
Vinblastine	0.03928441	0.02172129	0.05499869	0.02757024	0.12148536	0.09510855	0.40348383	0.00294766
Vincristine	0.00479192	0.00213871	0.00397938	0.00457641	0.00831196	0.00789993	0.00899849	0.00291558
Romidepsin	0.00653415	0.00432389	0.00325128	0.00445056	0.00466722	0.00232855	0.00467671	0.00482986
Palbociclib	>10	>10	>10	>10	0.15232759	2.91269778	>10	>10
Tazemetostat	>10	>10	>10	>10	4.88319087	>10	>10	>10
Ponatinib	2.81356652	2.0203162	0.92243724	2.84780338	0.49025917	0.95667784	1.59234381	0.89886604
GSK J4	2.97368976	3.51193576	3.19075107	2.20318272	3.45939297	2.90078037	7.02370006	5.95105123

Table S3. Significatly differentially expressed genes in MTX- and MTX+ SCCOHT tumoroids. Differentially expressed genes were filtered based on cutoff values of log2FoldChange > |1| and padj < 0.05.

Gene name	baseMean	log2FoldChange	lfcSE	stat	pvalue	padj
AL645608.1	18.3693067	1.47091193	0.48832722	3.01214407	0.00259409	0.0194519
ANKRD65	48.8644322	1.28510307	0.42264115	3.04064825	0.00236069	0.01811831
MIB2	1289.6079	1.0646857	0.35257261	3.01976291	0.00252973	0.01907347
CALML6	178.575749	1.92263419	0.42345217	4.54038101	5.62E-06	0.00015484
CFAP74	46.3733836	1.32610793	0.40612752	3.26525015	0.00109367	0.01019501
GABRD	146.976187	1.41405919	0.22606019	6.25523321	3.97E-10	4.44E-08
HES5	33.2484171	1.38482139	0.42917012	3.22674236	0.00125208	0.01128294
TNFRSF14	204.204541	2.33810685	0.37496954	6.23545812	4.50E-10	4.89E-08
PRDM16-DT	25.8797636	2.03874041	0.66655063	3.05864299	0.00222342	0.01735158
MEGF6	891.787896	1.59780416	0.28181113	5.66976945	1.43E-08	1.03E-06
HES2	201.80573	3.2295277	0.23329099	13.8433454	1.40E-43	7.61E-40
ESPN	201.532007	2.7051778	0.39538431	6.84189468	7.82E-12	1.47E-09
MIR34AHG	2321.43173	1.04697416	0.19004937	5.50895884	3.61E-08	2.32E-06
LNCTAM34A	207.508429	1.48633418	0.20000697	7.43141202	1.07E-13	2.79E-11
AL031731.1	30.1900561	2.41797689	0.36329733	6.65564184	2.82E-11	4.66E-09
DRAXIN	703.194699	4.7459569	0.2239828	21.1889348	1.21E-99	2.64E-95
TNFRSF1B	44.5653945	1.38049955	0.40292265	3.42621478	0.00061206	0.00656914
DHRS3	61.5902461	2.20717186	0.47186429	4.6775565	2.90E-06	9.01E-05
PRAMEF1	64.1746005	3.25753935	1.11980818	2.9090155	0.00362569	0.02498027
PRAMEF4	10.6506797	2.74177336	0.86928657	3.15405006	0.00161021	0.01360115
PRAMEF7	28.8166141	2.51520958	0.83707311	3.00476692	0.00265784	0.01977111
PRAMEF8	29.9678596	3.09570925	0.65979642	4.69191577	2.71E-06	8.51E-05
PRAMEF14	31.5831713	1.91673195	0.65088678	2.94480088	0.00323163	0.02283487
EPHA2	3477.07556	1.09425561	0.26236694	4.17070694	3.04E-05	0.00061578
MST1L	31.914718	2.01940301	0.39427727	5.12178406	3.03E-07	1.44E-05
ECE1-AS1	35.2845145	2.26360392	0.50960718	4.44186035	8.92E-06	0.00023112
ALPL	278.946502	1.02707521	0.30418137	3.37652236	0.00073408	0.00755127
WNT4	105.266981	3.26836363	0.39829831	8.20581849	2.29E-16	9.61E-14
AL445253.1	9.62029041	1.91080364	0.68750047	2.77934886	0.0054468	0.03357882
HTR1D	80.8461025	1.0732247	0.36283477	2.95788819	0.00309754	0.02216741
TCEA3	199.083958	1.16696481	0.2309465	5.05296592	4.35E-07	1.91E-05
GRHL3	208.346464	2.75078407	0.30496093	9.02011975	1.88E-19	1.37E-16
RSRP1	3701.27584	1.22476005	0.18316213	6.68675361	2.28E-11	3.92E-09
RHD	10.440657	2.15572438	0.77236926	2.79105409	0.00525367	0.03271549

EXTL1	54.9198026	1.17179865	0.37480267	3.12644155	0.00176936	0.01458533
AL353622.1	164.007388	1.33987933	0.29299679	4.57301717	4.81E-06	0.00013712
SESN2	2263.83795	1.21906491	0.18679607	6.52618056	6.75E-11	9.95E-09
OPRD1	213.398289	1.09642037	0.19254431	5.69437957	1.24E-08	9.22E-07
PTPRU	319.681463	1.33335627	0.2574969	5.17814486	2.24E-07	1.11E-05
MATN1-AS1	64.2154839	1.18198642	0.42609107	2.77402297	0.00553678	0.03402716
FNDC5	129.423496	1.38024624	0.24410928	5.65421463	1.57E-08	1.11E-06
GJB5	10.0750014	2.30937893	0.72160238	3.20034829	0.00137262	0.01205736
AL590434.2	21.0374689	3.39242933	0.74707896	4.54092472	5.60E-06	0.0001547
POU3F1	31.2045851	3.58035483	0.46739419	7.6602467	1.86E-14	5.78E-12
MIR3659HG	13.0486267	3.03243225	0.57571134	5.267279	1.38E-07	7.48E-06
AL139260.1	50.7042015	1.16772216	0.28881927	4.0430895	5.28E-05	0.00094082
RHBDL2	62.0407473	1.4311235	0.22738203	6.2939165	3.10E-10	3.59E-08
FAM183A	26.0058752	1.47190701	0.39974603	3.68210543	0.00023132	0.00307014
ARMH1	148.902026	1.8809207	0.40704844	4.62087684	3.82E-06	0.00011329
PLK3	1431.02949	2.34290679	0.27248619	8.59825888	8.09E-18	4.35E-15
TCTEX1D4	8.6459807	4.80764697	1.25413752	3.83342885	0.00012637	0.0018938
BTBD19	289.415556	4.69733063	0.3779168	12.4295363	1.81E-35	5.63E-32
BEND5	15.4166201	1.51350256	0.53142096	2.84802948	0.00439908	0.02864459
TTC39A	249.32814	1.09602687	0.29507694	3.71437654	0.00020371	0.00278396
PODN	72.5091257	3.25369748	0.98455883	3.30472634	0.00095069	0.00920324
SLC1A7	6.80557187	3.06622419	0.83293859	3.68121278	0.00023213	0.0030753
JUN	561.494486	1.08540012	0.33669935	3.22364777	0.00126569	0.01135335
DNAJC6	178.250888	1.20362873	0.35158462	3.42343965	0.00061834	0.00661705
GADD45A	540.481824	1.71754508	0.25925552	6.62491222	3.47E-11	5.54E-09
MSH4	32.7146174	1.54617037	0.42787015	3.61364393	0.00030192	0.00379649
AC103591.4	23.8209458	1.20599387	0.44228181	2.72675439	0.00639606	0.03778208
WDR63	185.385502	3.12540839	0.40767183	7.66648105	1.77E-14	5.63E-12
COL24A1	13.0825051	1.97710502	0.73063873	2.70599538	0.00681	0.03950922
SLC44A3-AS1	48.7935538	1.19247329	0.34296571	3.47694606	0.00050716	0.00568665
PLPPR5	5.96793694	2.72799236	0.89953787	3.03265981	0.00242409	0.01849426
PALMD	36.285973	1.05128988	0.39467552	2.66368153	0.00772907	0.04313469
RTCA-AS1	376.632502	1.02570699	0.29751204	3.44761502	0.00056556	0.0062075
AMY2B	201.197029	1.84034548	0.23616729	7.79255019	6.57E-15	2.24E-12
KIAA1324	80.9181597	1.1951388	0.35904875	3.32862543	0.00087276	0.00859184
KCNC4	722.823047	1.28485272	0.26778599	4.79805797	1.60E-06	5.62E-05
KCNA2	172.872124	1.20924721	0.3504374	3.45067963	0.00055918	0.00615602
AL360270.1	39.4013822	1.20144254	0.447368	2.68557997	0.0072404	0.04124012
DENND2D	24.0990978	1.47568308	0.44742441	3.29817295	0.00097316	0.00936261

PIFO	137.808878	1.07377524	0.27398419	3.91911393	8.89E-05	0.00144075
INKA2	780.711677	2.32118795	0.14272765	16.2630568	1.81E-59	1.97E-55
INKA2-AS1	25.6962276	1.74277272	0.4721627	3.69104276	0.00022334	0.00299153
LINC01356	18.3338049	1.77734437	0.55179067	3.22104826	0.00127723	0.01142175
NHLH2	12.8523	3.28273408	1.01153467	3.24530062	0.00117327	0.01076012
TENT5C	79.4425202	2.73267137	0.42202188	6.47518887	9.47E-11	1.35E-08
AC245014.1	6.03418797	3.21133194	0.99396632	3.23082571	0.00123433	0.01117093
POLR3GL	560.36942	1.54710549	0.21188269	7.30170769	2.84E-13	7.13E-11
TXNIP	50.1896079	1.56279349	0.51723556	3.02143476	0.0025158	0.01901445
LINC01719	81.5455548	1.06446669	0.25119534	4.23760517	2.26E-05	0.00049174
AC245100.4	61.2907436	1.27173466	0.38459668	3.30667089	0.00094412	0.00914365
H2BC18	53.8285055	1.52330361	0.3771901	4.03855667	5.38E-05	0.00095175
H4C14	13.0989148	1.72034246	0.62872736	2.73622967	0.00621476	0.03703062
H2BC21	1162.29268	1.38592316	0.33953965	4.08177122	4.47E-05	0.0008321
BNIPL	57.7380842	1.45381589	0.31877022	4.5607018	5.10E-06	0.00014316
KRT8P45	31.2507978	2.87737516	0.54483163	5.28121901	1.28E-07	7.04E-06
NHLH1	17.8413801	1.75037051	0.58534039	2.99034637	0.00278661	0.02050721
F11R	1303.51389	1.18964728	0.28045123	4.24190431	2.22E-05	0.00048554
NECTIN4	34.8624533	2.04529978	0.50295147	4.0665947	4.77E-05	0.00086961
NR1I3	14.5475499	1.39992121	0.4832197	2.89706982	0.00376666	0.02567588
AL391825.1	11.0298181	2.05176726	0.75091	2.73237441	0.00628796	0.0373321
ILDR2	454.518252	2.21535516	0.37201236	5.95505802	2.60E-09	2.32E-07
XCL1	59.3003157	1.15562253	0.28956919	3.9908338	6.58E-05	0.00112064
AL031736.2	14.7254611	1.96334761	0.62345817	3.14912484	0.0016376	0.01376694
MR1	482.370789	2.02607793	0.38621783	5.24594615	1.55E-07	8.23E-06
NPL	153.05333	1.61569377	0.46010788	3.51155421	0.00044549	0.00512154
NMNAT2	144.746623	1.11546974	0.39068399	2.85517133	0.00430136	0.02826186
AL096803.1	5.82537044	3.18025783	1.07890063	2.94768373	0.00320164	0.02268466
AC096633.1	25.4473785	1.77961338	0.41260625	4.31310326	1.61E-05	0.00037727
DDX59-AS1	8.23413357	2.13880227	0.80387024	2.66063122	0.00779943	0.04338099
PKP1	108.220637	1.52459314	0.43822817	3.47899389	0.0005033	0.00564628
PHLDA3	1960.95496	1.34379561	0.22896016	5.86912422	4.38E-09	3.68E-07
AC096677.1	23.0069401	1.57261051	0.40879702	3.84692266	0.00011961	0.00181784
RPS10P7	33.1335001	1.02383173	0.33298637	3.07469564	0.00210717	0.0167194
ELF3	125.151454	1.37724111	0.44942755	3.06443408	0.00218082	0.01712846
HNRNPA1P59	44.3515384	1.04970319	0.36371372	2.88606984	0.00390086	0.02633038
PPFIA4	283.218179	1.20251652	0.19107526	6.29341815	3.11E-10	3.59E-08
BTG2	5432.12848	2.30081389	0.18563357	12.3943846	2.80E-35	7.64E-32
PLEKHA6	43.3130939	2.26844389	0.62690238	3.61849621	0.00029632	0.00374389

AL512306.2	33.9559439	1.18908112	0.36742174	3.23628406	0.00121097	0.01100277
CNTN2	43.6927327	1.61096041	0.57405279	2.80629315	0.00501151	0.03156787
MFSD4A	129.66481	1.17380414	0.26897213	4.36403635	1.28E-05	0.00031129
HHAT	697.578203	1.52993464	0.20528793	7.45262822	9.15E-14	2.41E-11
ATF3	205.585034	1.85273631	0.40771315	4.54421529	5.51E-06	0.00015307
C1orf115	127.131879	1.2019965	0.45229937	2.65752417	0.00787169	0.04369381
DUSP10	92.0964414	3.30978719	0.96524765	3.42895129	0.00060592	0.00652896
AL591895.1	79.5017635	1.33805057	0.36439091	3.67201959	0.00024064	0.00315742
ITPKB	104.329276	1.2170709	0.35859182	3.39402862	0.00068872	0.00720076
AL731702.1	50.398023	2.15993377	0.42259512	5.11111861	3.20E-07	1.50E-05
AL359510.2	41.8871756	1.34578896	0.39834679	3.37843557	0.000729	0.00751023
TRIM67	14.911442	2.02788448	0.72692062	2.7896918	0.00527582	0.03282794
SIPA1L2	87.6587495	1.66518388	0.34644747	4.80645422	1.54E-06	5.42E-05
PCNX2	274.548335	1.14726647	0.43516122	2.63641706	0.00837867	0.04579723
PLD5	154.232346	1.90048903	0.46609729	4.07745137	4.55E-05	0.00084339
LINC01341	93.0514307	1.02060272	0.27536072	3.70642091	0.00021021	0.00285957
AC007240.3	32.565959	1.29432422	0.43401738	2.98219443	0.0028619	0.02093419
C2orf50	32.1744207	1.25094229	0.403608	3.09939912	0.00193914	0.01571757
TRIB2	24.3606345	1.52738288	0.48769607	3.13183345	0.00173718	0.01436353
LRATD1	18.3321762	2.07494844	0.68990871	3.00756956	0.00263346	0.01964515
TP53I3	5773.0973	1.97968811	0.15997558	12.374939	3.57E-35	8.66E-32
UCN	26.1316841	1.41236606	0.41773334	3.38102308	0.00072216	0.00745042
FOSL2	2196.88806	1.08048217	0.22809588	4.7369648	2.17E-06	7.15E-05
LBH	219.13426	1.82452357	0.37417932	4.87606731	1.08E-06	4.06E-05
XDH	175.733192	1.24867924	0.3836947	3.2543562	0.0011365	0.0104957
CDC42EP3	416.541849	1.16940979	0.39841541	2.93515201	0.00333384	0.0233905
CYP1B1	31.3205339	1.20063147	0.41126157	2.91938649	0.00350721	0.02433302
EPAS1	22.6028341	2.01447847	0.60842789	3.3109568	0.00092978	0.00904893
C2orf73	18.8792995	1.77187085	0.59490587	2.97840538	0.00289752	0.02115222
EML6	162.270026	1.48461226	0.31363035	4.73363717	2.21E-06	7.26E-05
CCDC85A	5.3273927	2.83560144	1.02285585	2.77223954	0.00556721	0.03412259
AC012368.1	33.473125	2.31237819	0.45769602	5.05221392	4.37E-07	1.91E-05
AC007318.3	42.609257	1.09843167	0.29653245	3.7042545	0.00021201	0.00287695
ARHGAP25	21.2515844	1.67011984	0.58553729	2.85228604	0.0043406	0.0284036
ANXA4	3985.11685	1.25868631	0.17726546	7.100573	1.24E-12	2.63E-10
TGFA	45.8054603	2.32217951	0.4062207	5.71654649	1.09E-08	8.24E-07
AC007040.2	30.5510091	1.89868365	0.55997854	3.39063644	0.00069731	0.00725593
ANKRD53	156.730436	1.49457699	0.40767655	3.66608524	0.00024629	0.00321417
LBX2	23.2036873	1.22637098	0.37889793	3.23667897	0.00120929	0.01099908

AC005041.3	10.9975863	2.14239452	0.67665048	3.16617601	0.00154457	0.01320117
DQX1	165.028151	1.01661472	0.29354713	3.46320787	0.00053378	0.00592459
RETSAT	3086.02259	1.31457651	0.18302253	7.18259376	6.84E-13	1.54E-10
FOXI3	15.4588759	3.29062567	0.72904187	4.51363055	6.37E-06	0.00017151
AC233266.2	43.9687049	1.99176576	0.31024489	6.41997918	1.36E-10	1.82E-08
KIAA1211L	9.86105496	2.49680431	0.69962163	3.56879233	0.00035863	0.00431385
TMEM182	140.073503	1.21219116	0.26167333	4.63245981	3.61E-06	0.00010845
FOXD4L1	7.86525425	2.54593274	0.83980728	3.03156783	0.00243287	0.01854887
MAP3K2-DT	121.929733	1.22774594	0.25456348	4.82294602	1.41E-06	5.08E-05
RAB6D	20.6089712	1.41161512	0.40633851	3.47398803	0.00051278	0.00573454
CXCR4	58.826465	2.2275475	0.51474035	4.3275168	1.51E-05	0.00035727
ACVR1C	15.5694004	1.63439009	0.50771776	3.21909183	0.00128597	0.01148115
GAD1	12.1104427	2.01299726	0.75472714	2.66718547	0.00764894	0.04284232
GPR155	390.591443	1.16520885	0.24232006	4.80855292	1.52E-06	5.39E-05
SLC40A1	218.32166	1.64638954	0.31913961	5.15883798	2.48E-07	1.22E-05
AC013468.1	18.5365635	1.49769464	0.44920021	3.3341361	0.00085565	0.00845189
STAT4	26.702608	2.39247557	0.42212336	5.66771654	1.45E-08	1.04E-06
NABP1	176.734221	1.39663279	0.28318021	4.93195763	8.14E-07	3.22E-05
C2orf66	149.594154	1.69153645	0.41355637	4.09021978	4.31E-05	0.00080787
CASP10	22.2470028	1.71344177	0.51655919	3.3170289	0.0009098	0.0088783
UNC80	120.890199	1.22315895	0.44461806	2.75103298	0.00594077	0.03576918
CATIP	171.86119	1.40686945	0.2377542	5.91732752	3.27E-09	2.86E-07
PTPRN	78.7114597	1.60375405	0.54861936	2.92325458	0.00346393	0.02414792
ASIC4	9.12463924	2.13303308	0.6735166	3.1670089	0.00154016	0.0131841
SCG2	131.216003	1.71953462	0.52193381	3.29454538	0.00098581	0.00944266
NPPC	35.3405698	2.12215438	0.52707697	4.02627034	5.67E-05	0.00099238
SNORC	107.669162	1.54770459	0.44783607	3.45596234	0.00054833	0.00605803
NGEF	42.575226	1.11874036	0.36950086	3.02770704	0.00246417	0.01876725
AGAP1-IT1	6.0213796	2.80645475	0.866218	3.23989427	0.00119574	0.01092133
GBX2	549.37942	2.23496327	0.73640522	3.03496391	0.00240564	0.0183857
KLHL30	6.51627203	2.6457299	0.99481121	2.65952963	0.00782498	0.04348985
ERFE	246.492624	1.59337113	0.26754675	5.95548668	2.59E-09	2.32E-07
AC133528.1	14.9663541	1.37125691	0.48189391	2.84555768	0.00443337	0.02879053
SRGAP3	361.803677	1.56396745	0.32341343	4.83581474	1.33E-06	4.79E-05
SLC6A11	43.7633738	1.36078734	0.48550411	2.80283384	0.00506558	0.03186207
HRH1	13.2624507	1.60775186	0.61748969	2.60369022	0.00922261	0.04931078
FBLN2	289.248444	1.07266701	0.36159306	2.96650329	0.00301207	0.02174112
AC093495.1	186.593667	1.10375876	0.26736399	4.12829998	3.65E-05	0.00071105
GRIP2	984.419026	3.09483604	0.19115182	16.1904604	5.89E-59	4.28E-55

RHBDF1P1	21.2169659	3.82420179	0.61536753	6.21450045	5.15E-10	5.51E-08
PLCL2	232.136373	3.03193656	0.39627335	7.65112405	1.99E-14	6.12E-12
EOMES	392.693581	1.65026707	0.61813515	2.66975123	0.00759075	0.04263171
TGFBR2	354.177929	1.9209207	0.71926466	2.67067298	0.00756994	0.04256016
PLCD1	595.800443	1.30052188	0.27671599	4.69984364	2.60E-06	8.23E-05
SLC22A14	8.19864422	3.90634901	0.90265785	4.32760763	1.51E-05	0.00035727
AC092053.4	33.1518397	1.05254045	0.37796037	2.78479054	0.00535623	0.03317427
AC092053.5	18.200469	1.13395003	0.39371691	2.88011511	0.0039753	0.02668955
UCN2	21.2492683	2.22399918	0.64712588	3.43673349	0.00058877	0.00638512
AC135506.1	25.1904917	1.1416299	0.40435529	2.82333364	0.00475271	0.03036723
GPX1	11736.0127	1.12073871	0.20208863	5.5457783	2.93E-08	1.94E-06
AMT	18.9767472	1.05192842	0.40475956	2.59889705	0.00935238	0.04973047
INKA1	57.7340108	2.07728257	0.34836665	5.96292024	2.48E-09	2.27E-07
SLC38A3	55.0229876	1.63903296	0.26843986	6.10577335	1.02E-09	1.03E-07
SEMA3B	199.09834	1.05715215	0.35200454	3.00323446	0.00267127	0.01983901
GRM2	58.1930623	1.27311541	0.394541	3.22682666	0.00125171	0.01128294
AC115284.2	44.2293746	2.5407782	0.49721583	5.11001064	3.22E-07	1.50E-05
SEMA3G	230.90033	2.0797916	0.32147839	6.46946003	9.84E-11	1.38E-08
DNAH12	22.0803073	1.35243536	0.47534233	2.84518185	0.00443861	0.02880738
AC012557.2	10.1150412	2.16101178	0.64682042	3.34097646	0.00083484	0.00828766
PRICKLE2	8.19587212	2.07826201	0.74254229	2.79884665	0.00512855	0.03212889
FRG2C	186.67253	3.20893672	0.95232524	3.36958068	0.00075283	0.00768179
MTRNR2L12	35.7796882	1.2595583	0.37688905	3.34198703	0.00083181	0.0082618
FILIP1L	82.6428721	1.47978668	0.37807775	3.91397447	9.08E-05	0.00146634
IMPG2	69.2898508	1.3898047	0.30777894	4.51559391	6.31E-06	0.0001703
PDCL3P4	45.8861714	1.52155668	0.2931247	5.19081699	2.09E-07	1.06E-05
DUBR	265.176831	1.35652527	0.38496685	3.5237457	0.00042549	0.00493843
CD47	1144.50464	1.15637798	0.36495737	3.16852893	0.00153213	0.0131618
NECTIN3-AS1	19.9162487	2.47301295	0.66061956	3.74347524	0.00018149	0.00253046
PLCXD2	13.9942893	1.8093925	0.62614279	2.88974419	0.00385555	0.02612651
C3orf52	21.2681142	1.79634636	0.65748222	2.73215962	0.00629206	0.0373321
CFAP44	457.933613	1.0437105	0.12051521	8.66040473	4.70E-18	2.70E-15
HCLS1	12.2886801	1.92762134	0.73885585	2.60892749	0.00908265	0.04863396
SLC15A2	53.5201829	1.54816135	0.28879485	5.36076503	8.29E-08	4.79E-06
LINC02614	245.225094	1.64502154	0.33416561	4.92277336	8.53E-07	3.35E-05
GATA2	522.841583	1.3706589	0.30348599	4.51638279	6.29E-06	0.00017008
TF	14.5237721	2.12604593	0.56036037	3.79406904	0.0001482	0.0021472
AC092969.1	6.86104683	3.17946696	0.85793437	3.70595591	0.00021059	0.00286126
SLC35G2	828.219534	1.27504278	0.2689622	4.74060207	2.13E-06	7.06E-05

FOXL2	147.673936	1.1310583	0.28846756	3.92092033	8.82E-05	0.00143319
ANKUB1	14.7870553	3.53997246	0.84863816	4.17135668	3.03E-05	0.0006146
SLC66A1L	37.9763952	1.3296669	0.383454	3.46760474	0.00052512	0.00584895
RARRES1	27.7951589	1.97479283	0.41879144	4.71545654	2.41E-06	7.82E-05
AC080013.1	35.582491	1.40418627	0.33870956	4.14569426	3.39E-05	0.00066936
IL12A	129.121166	1.32036364	0.43945876	3.00452227	0.00265998	0.01977541
ZBBX	66.7332432	1.90264288	0.3316413	5.73705046	9.63E-09	7.38E-07
CLDN11	50.2514047	1.10785784	0.40098998	2.76280683	0.00573067	0.03484476
ZMAT3	3195.10112	1.06769662	0.16744226	6.37650635	1.81E-10	2.29E-08
LAMP3	562.618711	1.74927608	0.40674244	4.30069725	1.70E-05	0.00039355
EPHB3	149.873664	1.20902568	0.24865557	4.86225047	1.16E-06	4.28E-05
AC107294.3	9.8035906	2.15751379	0.72666699	2.96905434	0.00298718	0.02161148
BCL6	175.414359	1.61087084	0.41404088	3.89060815	1.00E-04	0.0015822
CLDN1	53.1596094	1.30710687	0.49262366	2.6533579	0.00796953	0.04409334
CLDN16	7.64635686	3.18743952	1.11808754	2.85079604	0.00436099	0.02846699
IL1RAP	101.237323	1.12763179	0.31766965	3.54969944	0.00038567	0.0045513
FAM43A	121.024677	1.59528911	0.32060691	4.97584134	6.50E-07	2.65E-05
AC069213.1	12.0125486	1.81800684	0.49588439	3.66619091	0.00024619	0.00321417
MUC20	64.1153942	1.21050914	0.44790737	2.70258811	0.00688019	0.03986348
IDUA	377.257554	1.55434701	0.58249419	2.66843349	0.00762059	0.04273482
RNF212	453.101267	1.71437022	0.23967125	7.15300742	8.49E-13	1.87E-10
MSANTD1	20.7519555	1.40933715	0.41466171	3.39876369	0.00067691	0.00710106
LINC02600	47.4798175	1.22617344	0.41962657	2.92205867	0.00347726	0.02419446
NSG1	515.024944	1.49968741	0.25996221	5.76886702	7.98E-09	6.25E-07
CYTL1	35.7667913	1.86542491	0.65870837	2.83194353	0.0046266	0.02973563
SORCS2	18.1403976	1.81089148	0.4941555	3.66461866	0.00024771	0.00322878
CPEB2	221.113569	1.07382576	0.33923556	3.16542807	0.00154855	0.01322479
SLC34A2	345.290963	1.30655421	0.32075397	4.07338442	4.63E-05	0.00085175
LIMCH1	68.2158059	1.31110708	0.50067704	2.61866825	0.00882737	0.04766477
NIPAL1	32.5807973	2.5305317	0.58422384	4.331442	1.48E-05	0.00035172
LRRC66	80.1420624	1.13830584	0.28942307	3.93301697	8.39E-05	0.00137417
SPATA18	1052.31029	1.5236182	0.14340841	10.6243298	2.30E-26	2.95E-23
IGFBP7	59.2051614	1.45562062	0.42788656	3.40188444	0.00066923	0.00704078
SLC4A4	31.9163733	1.46017988	0.4905508	2.97661296	0.00291452	0.02121947
AREG	16.9283051	4.02039303	0.84410079	4.76293004	1.91E-06	6.50E-05
PARM1	43.5549092	1.46134596	0.46427582	3.14758145	0.00164627	0.01381602
SCD5	921.793193	1.25955311	0.22654518	5.55983182	2.70E-08	1.81E-06
HPSE	56.4724303	1.7874701	0.40228905	4.44324815	8.86E-06	0.00022999
HERC5	1390.25602	1.01911386	0.33038615	3.08461435	0.00203816	0.01633225

NAP1L5	137.005021	1.11358617	0.35981062	3.09492304	0.00196864	0.0158985
AC093866.1	1806.37965	1.42725443	0.30667556	4.65395555	3.26E-06	9.93E-05
AC097478.2	56.7666257	1.69479197	0.58431192	2.90049183	0.00372578	0.02547678
TSPAN5	167.694666	1.34123763	0.19434478	6.90133089	5.15E-12	1.01E-09
NPNT	222.438561	1.72773744	0.39092737	4.4195868	9.89E-06	0.00025237
PITX2	9.39586286	2.63236305	0.79230176	3.32242483	0.00089239	0.00873571
FAM241A	40.0987834	1.5465287	0.54231367	2.85172361	0.00434829	0.02840772
AC026402.2	42.5124532	2.02946868	0.58800281	3.45146084	0.00055756	0.00614443
AC093591.2	18.4763812	1.62976001	0.43283822	3.76528672	0.00016636	0.0023571
LINC02615	117.939945	1.27315272	0.30610157	4.15924923	3.19E-05	0.00063948
GUSBP5	17.4463983	1.80918307	0.49926182	3.62371605	0.0002904	0.00368618
EDNRA	14.3847941	2.13956073	0.44475828	4.8106147	1.50E-06	5.35E-05
AC093908.1	8.58944769	2.29039141	0.62855959	3.64387317	0.00026857	0.00346106
FHDC1	91.4873399	1.088205	0.36848836	2.95315976	0.00314539	0.02240403
TLR2	21.434191	2.3399428	0.56618774	4.13280374	3.58E-05	0.00069942
SPOCK3	11.0815807	2.08132503	0.76741674	2.71211836	0.00668547	0.03902541
PALLD	613.747761	1.38239776	0.24363688	5.67400857	1.39E-08	1.01E-06
C4orf47	55.8841171	2.16055012	0.54852252	3.93885398	8.19E-05	0.00134724
AC021087.5	36.7841202	1.11696783	0.33654264	3.31894892	0.00090357	0.00882538
NKD2	96.6897057	1.37490108	0.35463115	3.87698903	0.00010576	0.00165539
SLC6A3	11.457201	3.80644828	0.99541966	3.82396335	0.00013132	0.00194174
AC026412.2	25.9808746	1.59696234	0.59712894	2.67440115	0.00748628	0.04224223
C5orf49	15.8068278	1.19365202	0.44277756	2.6958277	0.0070214	0.04046509
CMBL	6324.18671	1.39655182	0.17472367	7.99291704	1.32E-15	4.87E-13
OTULINL	532.940977	1.62217803	0.28726563	5.64696172	1.63E-08	1.15E-06
AC010491.1	43.3328991	1.20565923	0.36012986	3.34784575	0.00081442	0.00814423
H3Y2	28.3304301	2.06334698	0.72712725	2.83766973	0.00454442	0.02932836
H3P18	24.4174944	2.51001636	0.75885816	3.30762257	0.00094092	0.00912073
TAF11L11	301.420637	1.87269508	0.69591047	2.69100002	0.00712382	0.04085066
H3Y1	603.614843	2.25314728	0.7854437	2.86862991	0.00412254	0.0274333
PURPL	367.982392	1.0200862	0.20543154	4.96557728	6.85E-07	2.78E-05
FST	38.4091204	1.76448821	0.64664234	2.72869267	0.00635859	0.03762053
PLK2	2739.5237	1.91430388	0.60667125	3.15542214	0.00160266	0.01356029
MAST4	50.3285493	3.96215174	0.65779186	6.02341258	1.71E-09	1.64E-07
ANKRA2	1238.10324	2.11333221	0.18592237	11.3667454	6.12E-30	8.35E-27
POLD2P1	8.75980356	1.96984432	0.62872987	3.13305352	0.00172998	0.01433114
ELL2	378.414063	1.2573795	0.25611278	4.90947581	9.13E-07	3.53E-05
AC116347.1	35.0611143	1.07668709	0.39629181	2.71690474	0.00658956	0.03868285
GRAMD2B	398.85928	1.30704454	0.29391624	4.44699674	8.71E-06	0.0002268

ACSL6	42.6380121	1.39465785	0.38313624	3.64010946	0.00027252	0.00350202
P4HA2	2210.91489	1.04810575	0.21487713	4.87769801	1.07E-06	4.04E-05
AC063976.2	16.2253994	1.91891439	0.50873047	3.77196672	0.00016197	0.00230535
IRF1	504.978869	1.32019314	0.29475814	4.47890302	7.50E-06	0.00019844
FBXL21P	8.11640105	5.64049459	1.08837281	5.1825023	2.19E-07	1.09E-05
TGFBI	47.7796165	2.0318605	0.51938521	3.91204919	9.15E-05	0.00147357
HBEGF	792.930451	1.30326276	0.28732702	4.53581687	5.74E-06	0.00015749
SRA1	2475.69516	1.13282579	0.13031902	8.69271229	3.54E-18	2.14E-15
PCDHB3	33.4909682	1.27865722	0.45023411	2.83998299	0.00451159	0.02916829
PCDHB4	37.5714185	1.50392903	0.55317889	2.71870285	0.00655385	0.03853541
PCDHB7	9.70615725	2.09787779	0.69708166	3.0095151	0.00261665	0.01955989
PCDHB10	55.6532851	1.67514826	0.36133524	4.63599477	3.55E-06	0.00010691
PCDHB14	192.898475	1.13554209	0.32933005	3.4480367	0.00056468	0.00620094
AC005753.3	6.59590654	3.75147924	0.97340513	3.85397522	0.00011622	0.00177454
AC091825.3	49.829178	1.02417728	0.30560263	3.35133662	0.00080422	0.00807556
FAXDC2	663.862008	1.83677359	0.35325624	5.19955024	2.00E-07	1.02E-05
CYFIP2	6394.85488	1.43629829	0.19031484	7.54695907	4.46E-14	1.26E-11
SLIT3	14.1954395	2.50091718	0.53954136	4.63526502	3.56E-06	0.00010714
FGF18	45.9435932	1.62500725	0.4936956	3.29151656	0.00099649	0.00952403
FAM153CP	76.2787336	1.04999634	0.27500691	3.81807252	0.0001345	0.00198294
PXDC1	108.360588	1.09637404	0.31397638	3.49189977	0.0004796	0.00544193
AL391422.4	26.2628684	1.75694022	0.52207119	3.36532687	0.00076453	0.00776632
RNA5SP202	5.59716061	3.81147203	1.12440264	3.38977506	0.0006995	0.00727507
AL031123.2	34.7580368	1.23446879	0.41819895	2.95186964	0.00315856	0.02245677
AL358777.3	76.4383318	1.14221993	0.30245672	3.77647392	0.00015906	0.0022753
AL024498.1	62.5943848	1.09787076	0.30423907	3.60857916	0.00030788	0.00384981
CD83	333.062874	1.73380907	0.28847357	6.0102875	1.85E-09	1.75E-07
RBM24	211.583685	1.39897654	0.33196233	4.21426285	2.51E-05	0.00053191
H1-2	1627.76979	1.09258146	0.37916435	2.88155112	0.00395723	0.02661738
H2BC4	93.9578503	1.17226121	0.43461355	2.69724956	0.00699149	0.040349
H2BC5	609.775101	1.25541044	0.31703658	3.95982829	7.50E-05	0.00124863
H2BC6	41.648017	1.06641906	0.37495119	2.84415437	0.00445295	0.0288661
AL031777.2	30.2150996	1.47718505	0.52405621	2.81875309	0.00482106	0.03071401
H2BC8	456.024901	1.33117546	0.39856942	3.33988353	0.00083814	0.00831656
H4C8	539.162721	1.36557987	0.33382004	4.09076657	4.30E-05	0.00080666
BTN3A2	125.676931	1.13264709	0.4033585	2.80804067	0.00498439	0.0314692
BTN3A1	677.713432	1.64522095	0.50030425	3.28844091	0.00100744	0.00960382
BTN3A3	142.487678	1.12156795	0.19891885	5.6383191	1.72E-08	1.20E-06
LINC00240	47.4784517	1.06184847	0.30951616	3.43067212	0.00060209	0.0064941

AL021807.1	132.559842	1.4691381	0.35262674	4.16626965	3.10E-05	0.00062474
H2BC11	362.681285	1.0401459	0.2707804	3.84128953	0.00012239	0.00184941
H2AC11	857.370122	1.04300006	0.24409879	4.27286048	1.93E-05	0.00043456
H4C9	967.78202	1.10176354	0.23337465	4.72100785	2.35E-06	7.64E-05
H2AC13	77.7824056	1.19902051	0.32785487	3.65716855	0.00025502	0.00331415
H2BC15	154.701096	1.02066904	0.27311562	3.73713174	0.00018613	0.00259017
HLA-J	25.0980183	1.08190619	0.40331482	2.68253518	0.00730665	0.0415185
PAIP1P1	12.4517317	1.40553484	0.46675173	3.01131145	0.00260122	0.01949128
HCG27	78.7153623	1.14615089	0.28068087	4.08346631	4.44E-05	0.00082746
HLA-C	6014.96481	1.03697591	0.33846464	3.06376433	0.00218571	0.01714313
HLA-B	2645.69204	1.89206146	0.57520809	3.28935124	0.00100419	0.00958248
HCP5	54.449208	1.44294822	0.37006281	3.89919816	9.65E-05	0.00153378
C2	23.1750274	1.97728922	0.43647813	4.53009917	5.90E-06	0.000161
PSMB8-AS1	15.5709968	1.92522436	0.67840412	2.83787245	0.00454153	0.02931842
HLA-DMB	18.5033451	3.17350569	0.69440571	4.57010309	4.87E-06	0.00013868
HLA-DOA	62.1914008	3.57939718	0.46960609	7.62212687	2.50E-14	7.46E-12
COL11A2	181.951726	1.32384112	0.35578442	3.72090807	0.00019851	0.00272932
AL645940.1	26.4630998	1.4435205	0.40072776	3.60224729	0.00031548	0.00392012
IP6K3	6.90843471	2.74864737	1.02793966	2.67393844	0.00749662	0.04225922
DEF6	98.8204791	2.17788093	0.36396587	5.98375048	2.18E-09	2.03E-07
MAPK13	243.753934	1.04815424	0.36283417	2.88879694	0.00386719	0.0261812
ETV7	9.89436873	2.07040431	0.66761277	3.10120537	0.00192735	0.01565651
CDKN1A	19081.2399	2.63019926	0.30594162	8.59706272	8.18E-18	4.35E-15
POLH	4450.8074	1.40293164	0.15280341	9.18128489	4.26E-20	3.44E-17
TMEM63B	5801.78301	1.05096565	0.25787339	4.07551024	4.59E-05	0.0008468
TCTE1	7.85378243	3.05707668	0.79572829	3.84185996	0.00012211	0.00184639
SLC25A27	92.2410394	1.15117192	0.35729504	3.22190847	0.0012734	0.01140622
PTCHD4	1058.6013	1.69177215	0.58785958	2.87785078	0.00400394	0.02684883
AL353138.1	31.1717853	2.37124741	0.52420703	4.52349406	6.08E-06	0.00016467
KLHL31	28.3250229	1.00343594	0.32611038	3.07698248	0.00209108	0.01662196
AL445250.1	19.7979385	2.21012566	0.64995959	3.40040471	0.00067286	0.00706537
LGSN	251.482523	1.1276425	0.33822411	3.33400975	0.00085604	0.00845191
PTP4A1	30.9178247	1.79607974	0.51324797	3.4994386	0.00046624	0.00531522
AL135905.1	134.562434	1.9199557	0.22081891	8.69470673	3.48E-18	2.14E-15
MYO6	2093.26586	2.11038534	0.5086907	4.14866114	3.34E-05	0.00066454
IMPG1	6.12578179	2.22020084	0.80638487	2.75327691	0.0059002	0.03558383
SH3BGRL2	2730.20914	1.17804792	0.30184182	3.9028652	9.51E-05	0.00151755
NT5E	143.209261	1.14443701	0.37409957	3.05917754	0.00221946	0.01734546
AL450338.2	7.78212935	3.29471133	0.91904474	3.58493028	0.00033717	0.00413083

Z98200.2	15.2775701	1.41959197	0.52845971	2.68628231	0.0072252	0.04120625
SESN1	2754.39706	1.22460583	0.12457259	9.83045942	8.32E-23	8.26E-20
CCDC162P	188.701305	1.08098599	0.19882462	5.43688188	5.42E-08	3.32E-06
AL080317.1	37.2857678	1.03080333	0.31097161	3.31478274	0.00091714	0.00894195
TRAF3IP2	273.083067	1.34237935	0.29799172	4.50475383	6.64E-06	0.00017769
RFPL4B	238.945744	2.29013783	0.77732446	2.94618008	0.00321725	0.0227554
PTPRK	100.800813	1.04664525	0.3476879	3.01030108	0.00260989	0.01952941
SLC2A12	207.877989	1.32909841	0.31131672	4.26928053	1.96E-05	0.00043887
ALDH8A1	82.3193572	1.14734255	0.2409932	4.76089189	1.93E-06	6.55E-05
ULBP2	12.3548926	2.10646853	0.65330725	3.22431525	0.00126274	0.01134804
ULBP1	222.038458	1.50727073	0.44808506	3.36380496	0.00076876	0.00778752
PPP1R14C	316.545124	1.38916759	0.31573124	4.3998421	1.08E-05	0.00027295
FRMD1	7.37835528	3.08893384	0.90830312	3.40077423	0.00067195	0.00705923
AC073343.2	61.122009	1.02916178	0.30958474	3.3243298	0.00088631	0.00868794
LINC02587	83.1229147	1.33754865	0.43051536	3.10685466	0.00189089	0.01541827
CREB5	8.92810105	1.78442317	0.66991278	2.66366491	0.00772945	0.04313469
MTURN	626.42412	1.68452137	0.14341621	11.7456833	7.43E-32	1.25E-28
LINC01176	20.8606063	2.57128724	0.66235913	3.88201376	0.0001036	0.00162974
AC005154.1	10.1217412	1.54837683	0.58314436	2.65522044	0.00792566	0.04391516
ADCYAP1R1	38.3003018	2.75553473	0.64944367	4.24291569	2.21E-05	0.00048384
AC018641.1	18.9646942	2.18764289	0.461159	4.74379317	2.10E-06	6.99E-05
ELMO1	43.2669935	1.47389201	0.5527208	2.66661216	0.007662	0.04285694
SFRP4	2056.99238	1.15132728	0.33747546	3.41158814	0.00064586	0.00684106
INHBA	14.2987903	2.46554395	0.64970449	3.79486979	0.00014772	0.00214171
IGFBP3	44.7370673	1.87606428	0.68027561	2.75780029	0.00581917	0.03523151
TRIM74	6.1242018	1.83479316	0.64252748	2.85558706	0.00429573	0.02824132
VPS37D	258.766473	1.20173785	0.32534435	3.69374123	0.00022098	0.00297216
SPDYE12P	25.7059726	1.12413943	0.35128831	3.20004795	0.00137405	0.01206507
GTF2IRD2B	61.5853293	1.03507278	0.24632886	4.20199558	2.65E-05	0.00055191
TRIM73	12.2182246	2.12348501	0.79326236	2.67690124	0.00743065	0.04200437
CCDC146	107.518148	1.1437558	0.26384978	4.33487499	1.46E-05	0.0003477
GNGT1	44.2212566	1.26401487	0.46097495	2.74204676	0.00610576	0.0365709
TMEM130	57.6205924	1.23053773	0.3964821	3.10364004	0.00191156	0.0155519
CASTOR3	698.484082	1.11483439	0.13595379	8.20009822	2.40E-16	9.89E-14
C7orf61	12.9092378	2.27002077	0.61598922	3.68516313	0.00022856	0.00304091
ACHE	120.257968	2.0189782	0.2682715	7.52587666	5.24E-14	1.45E-11
SERPINE1	183.508375	1.82319185	0.47134851	3.86803355	0.00010972	0.00170566
SLC26A5	10.8314608	1.96571296	0.60703383	3.23822638	0.00120275	0.01095786
LSMEM1	147.117253	1.13603906	0.25587058	4.43989716	9.00E-06	0.00023268

TSPAN12	64.3325606	1.10005738	0.32772528	3.35664488	0.00078894	0.00795139
SSU72P8	28.2726674	2.28460644	0.64335231	3.55109698	0.00038363	0.00453701
LRRC4	310.09218	1.11776421	0.19831537	5.63629639	1.74E-08	1.21E-06
FAM71F2	159.894956	1.45717149	0.31144312	4.67877239	2.89E-06	8.97E-05
AC018638.6	40.2835279	2.1287065	0.52160802	4.08104631	4.48E-05	0.00083328
CPA4	194.765336	2.99705327	0.80560925	3.72023195	0.00019904	0.00273492
KLF14	18.7603869	1.88217069	0.4818538	3.90610322	9.38E-05	0.00150157
PODXL	236.201511	1.2082406	0.22851487	5.28736086	1.24E-07	6.85E-06
CALD1	2633.27941	1.29066699	0.30063627	4.29311802	1.76E-05	0.00040381
PTN	30.7228763	1.26180657	0.43302098	2.91396178	0.00356874	0.02467358
CLEC2L	111.072259	1.42945914	0.34670007	4.12304252	3.74E-05	0.0007252
KDM7A-DT	153.84447	1.18518724	0.26978953	4.39300679	1.12E-05	0.0002791
EPHB6	13.8037356	3.11469983	0.72419427	4.30091753	1.70E-05	0.00039355
ZNF425	421.5679	1.04954865	0.18193845	5.7687017	7.99E-09	6.25E-07
AC004877.2	16.4423735	1.38825675	0.48630399	2.8547098	0.00430762	0.02827684
AC006017.1	20.8454571	1.17034498	0.39363718	2.97315658	0.00294754	0.02141703
CSF2RA	67.9939592	1.48965146	0.43927967	3.39112314	0.00069607	0.00725334
GPR143	77.8282714	1.648581	0.37037036	4.45116886	8.54E-06	0.00022303
SHROOM2	233.423418	1.04640107	0.27928426	3.74672415	0.00017916	0.00250432
GPX1P1	138.639718	1.09289506	0.37097022	2.94604527	0.00321865	0.02275794
SAT1	4076.21752	1.25128229	0.16479259	7.59307398	3.12E-14	9.21E-12
PRRG1	521.546032	1.62626534	0.39668518	4.09963728	4.14E-05	0.00078377
SRPX	29.0410245	2.55027887	0.50004445	5.10010436	3.39E-07	1.55E-05
PINCR	5.81594958	3.66029434	1.18939265	3.07744827	0.00208781	0.01660794
TIMP1	409.981674	1.61912906	0.30036517	5.39053541	7.02E-08	4.12E-06
PIM2	1695.14623	1.04337721	0.10409821	10.0230085	1.21E-23	1.32E-20
KCND1	220.302844	1.16835768	0.38210915	3.05765431	0.00223077	0.01739026
TSPYL2	2764.03631	1.03963706	0.1805978	5.75664286	8.58E-09	6.66E-07
KLF8	714.782515	2.08398218	0.64007832	3.25582373	0.00113064	0.01046128
EFNB1	980.884098	1.1081747	0.21111667	5.24911041	1.53E-07	8.13E-06
NHSL2	172.866508	1.13960552	0.23431588	4.86354377	1.15E-06	4.26E-05
RTL5	1100.15601	1.4816769	0.21884995	6.7702866	1.29E-11	2.26E-09
MAGEE2	9.6167621	1.86810808	0.71489092	2.61313724	0.00897153	0.04815713
ITM2A	27.4379688	2.42730112	0.57727366	4.20476679	2.61E-05	0.00054919
HMGN5	285.594554	1.03495176	0.38651944	2.67761889	0.00741475	0.04195797
KLHL4	20.8432329	1.71670451	0.63396644	2.70787915	0.00677147	0.03933797
NAP1L3	215.711551	1.54949425	0.41100447	3.77001802	0.00016324	0.00231737
NANOGNBP3	8.99090108	1.6660609	0.61508164	2.70868252	0.00675509	0.03931575
AL035427.2	8.49626573	1.75274893	0.64667732	2.71039186	0.00672038	0.03916629

AL021308.1	10.5559869	2.09556727	0.6607025	3.17172594	0.00151536	0.01305375
MORC4	1104.02647	1.20261987	0.16961594	7.0902527	1.34E-12	2.78E-10
NUP62CL	48.1885946	2.148695	0.602793	3.56456529	0.00036446	0.00436611
TSC22D3	184.634148	1.04102306	0.2654845	3.9212197	8.81E-05	0.0014327
ZCCHC12	1102.47461	1.08899942	0.31851479	3.41899168	0.00062854	0.00670644
AC005052.2	32.994884	1.03399082	0.39537152	2.61523852	0.00891652	0.0479444
AL139234.1	12.3778389	1.40936148	0.53622829	2.6282863	0.00858163	0.04662118
SMIM10L2A	101.329141	1.3672861	0.28292814	4.83262683	1.35E-06	4.85E-05
INTS6L	906.431684	1.06302992	0.28333178	3.75189083	0.00017551	0.00246273
SLITRK4	29.4558938	1.99387742	0.75372491	2.64536489	0.00816029	0.04487335
ZFP92	120.562969	2.20630653	0.44415653	4.96740765	6.79E-07	2.76E-05
BGN	9.06089043	2.86509045	0.93464999	3.06541539	0.00217368	0.01707946
PNCK	33.3358507	1.10108975	0.38591071	2.85322412	0.00432781	0.02835819
U52111.1	13.2366294	2.14625242	0.57832484	3.71115381	0.00020632	0.00281384
PLXNB3	425.136265	3.05951763	0.5328947	5.74131746	9.39E-09	7.22E-07
GAB3	70.2242372	1.50390975	0.5124878	2.93452788	0.00334056	0.02341501
CLIC2	47.5544957	1.16164193	0.41720808	2.78432271	0.00536396	0.0331939
AF233439.1	41.5781569	1.51730355	0.36432294	4.16472139	3.12E-05	0.00062749
AC011008.2	28.9102948	1.00170661	0.36903616	2.71438603	0.00663987	0.03890496
AF131215.7	13.2351093	1.42601314	0.51173921	2.7866013	0.0053264	0.03301761
SLC35G5	9.95754497	1.90585339	0.71167158	2.67799564	0.00740642	0.04193014
DEFB109D	24.1802447	1.06409361	0.40880328	2.60294781	0.0092426	0.04940558
AC068587.4	115.200385	1.60916162	0.30428137	5.2884	1.23E-07	6.83E-06
GFRA2	15.4337052	2.38398309	0.87245136	2.73251117	0.00628535	0.0373321
PHYHIP	10.1788858	2.37757271	0.71012939	3.3480838	0.00081372	0.00814423
TNFRSF10B	11997.7951	1.03877932	0.13003711	7.98832954	1.37E-15	4.97E-13
TNFRSF10D	4086.21576	1.45065442	0.21767375	6.66435157	2.66E-11	4.43E-09
TNFRSF10A	1021.53662	1.14107399	0.27936651	4.08450531	4.42E-05	0.00082447
HTRA4	86.7547844	3.46293071	0.8459726	4.09343127	4.25E-05	0.00080089
ANK1	401.864845	2.06654572	0.31519632	6.55637633	5.51E-11	8.41E-09
ADHFE1	66.998388	1.10826619	0.35973916	3.08074937	0.0020648	0.01647313
LINC01607	19.5045101	1.52449258	0.52212297	2.91979604	0.00350261	0.02432778
FABP4	75.4394696	1.84676713	0.62311106	2.96378489	0.00303881	0.0218762
LINC00535	46.1445466	1.11956897	0.36450632	3.07146655	0.0021301	0.01681577
TP53INP1	5740.8088	1.81190401	0.22394628	8.09079737	5.93E-16	2.27E-13
MATN2	1297.47859	1.18378233	0.27950478	4.23528479	2.28E-05	0.0004951
KCNS2	12.6631721	2.25027295	0.86041642	2.61533009	0.00891413	0.0479444
ZNNT1	70.2822068	1.39321971	0.34279937	4.06424238	4.82E-05	0.00087696
UBR5-AS1	86.4762772	1.13648064	0.29428566	3.86182814	0.00011254	0.00173668

CTHRC1	100.91532	1.04395492	0.19602453	5.32563402	1.01E-07	5.72E-06
AC027031.2	104.381187	1.30199687	0.42192586	3.08584279	0.00202976	0.01628885
RSPO2	17.4704421	1.90359407	0.49667758	3.83265551	0.00012677	0.00189847
HMGB1P19	7.124311	2.14779071	0.80554477	2.66625867	0.00767007	0.04289104
LRATD2	53.8799598	2.26754776	0.47388275	4.78503969	1.71E-06	5.91E-05
TG	15.5311714	2.74802451	0.9251419	2.97038165	0.0029743	0.02155612
NDRG1	370.725233	1.25095122	0.36445767	3.4323635	0.00059834	0.00647221
ADGRB1	628.259368	1.03199089	0.28728821	3.59217975	0.00032792	0.00404062
ARC	357.781335	2.31100007	0.78252957	2.95324313	0.00314454	0.02240403
MAFA	88.3884692	2.67514956	0.43739286	6.11612527	9.59E-10	9.87E-08
IQANK1	284.367846	1.25066848	0.22804198	5.48437832	4.15E-08	2.59E-06
EPPK1	139.537535	1.74536318	0.37104973	4.70385242	2.55E-06	8.13E-05
ZNF252P-AS1	24.1889287	1.44560449	0.47261139	3.05875933	0.00222256	0.01735105
DOCK8-AS1	51.9219538	1.298672	0.41882678	3.10073777	0.00193039	0.01566185
DOCK8	668.383347	1.28214068	0.32924789	3.89415004	9.85E-05	0.00156153
VLDLR	81.9591092	1.31624452	0.31251794	4.21174074	2.53E-05	0.00053632
AL583785.1	157.404456	1.22031972	0.45569549	2.677928	0.00740791	0.04193014
ACER2	450.64532	1.60807224	0.22774887	7.06072532	1.66E-12	3.38E-10
AL158206.1	501.132144	1.98330576	0.22433654	8.84076117	9.51E-19	6.69E-16
AQP3	99.7938404	1.52518904	0.40986474	3.72120088	0.00019828	0.00272932
ENHO	459.518522	1.0910559	0.16161339	6.75102396	1.47E-11	2.56E-09
CNTFR	94.7592859	1.19527731	0.35288298	3.38717754	0.00070616	0.00733079
AL162231.2	203.210015	1.41982545	0.17249411	8.23115309	1.85E-16	8.26E-14
FAM205A	12.9008473	2.91522482	0.75982388	3.83671121	0.00012469	0.00187512
RMRP	29.1381591	1.12645788	0.35390409	3.18294675	0.00145784	0.01266832
GLIPR2	737.974384	1.04057528	0.21631707	4.81041678	1.51E-06	5.35E-05
IGFBPL1	243.335233	1.11798064	0.35200097	3.17607255	0.00149284	0.01288516
BMS1P10	42.003097	1.31849871	0.37016515	3.56192015	0.00036815	0.00438727
DUX4L50	304.295574	1.28704749	0.28569701	4.50493869	6.64E-06	0.00017769
MIR4477B	18.4775354	1.42616598	0.41343631	3.44954217	0.00056154	0.00617579
CBWD4P	23.4320657	1.30713433	0.38596387	3.38667537	0.00070745	0.00734026
CBWD3	115.485911	1.55317013	0.25439545	6.10533767	1.03E-09	1.03E-07
TRPM6	439.668353	1.10996996	0.36518927	3.03943751	0.0023702	0.0181785
NTRK2	20.2629865	2.84561838	0.71363638	3.98749062	6.68E-05	0.00113389
NXNL2	95.949525	1.00945712	0.36418768	2.77180468	0.00557465	0.03414451
S1PR3	1797.87017	1.26735358	0.18012297	7.03604625	1.98E-12	4.00E-10
ANKRD19P	25.6356365	1.39296191	0.3589177	3.88100649	0.00010403	0.00163414
AL589843.2	81.5247866	1.2710029	0.30320071	4.19195225	2.77E-05	0.00056877
GABBR2	141.68225	1.16441741	0.24425327	4.76725414	1.87E-06	6.37E-05

COL15A1	9.91536218	1.442092	0.55083619	2.6180052	0.00884455	0.04769269
STX17-AS1	34.6000745	1.32745139	0.37902217	3.50230537	0.00046125	0.00526435
NR4A3	1139.11649	2.62464913	0.63885738	4.10834909	3.98E-05	0.00076341
PLPPR1	12.165321	2.39065782	0.83197126	2.87348606	0.00405969	0.0271143
ABCA1	726.445967	1.6215123	0.32163792	5.04142143	4.62E-07	1.99E-05
FRRS1L	443.418107	1.0327316	0.21640947	4.77211831	1.82E-06	6.23E-05
PALM2AKAP2	584.133454	1.13482411	0.3234973	3.50798638	0.00045151	0.00517709
WHRN	387.021668	1.13121882	0.36759601	3.07734252	0.00208855	0.01660794
PAPPA	24.8215897	1.05103668	0.3833564	2.74166984	0.00611278	0.03660284
ASTN2	1455.07907	1.66995524	0.17084482	9.77469037	1.45E-22	1.37E-19
BRINP1	15.6203625	1.55170524	0.4748476	3.26779633	0.00108388	0.01015007
AL359636.2	12.6874604	2.49946771	0.94412807	2.64738206	0.00811177	0.04469664
NR5A1	93.7683875	1.20998434	0.31224664	3.87509168	0.00010658	0.00166715
ANGPTL2	169.463237	1.13949194	0.42669934	2.67047973	0.00757429	0.0425737
AL445222.2	42.1629615	1.12850739	0.31217452	3.61498883	0.00030036	0.0037818
ZNF79	769.955378	1.24067789	0.19175039	6.47027553	9.78E-11	1.38E-08
AK1	348.770832	1.40034316	0.24159878	5.79615162	6.79E-09	5.48E-07
AL590708.1	15.5775575	1.331481	0.48284101	2.75759717	0.00582279	0.03524363
PTGES	213.653983	1.05474675	0.2313117	4.5598503	5.12E-06	0.00014338
RALGDS	545.306444	1.31205846	0.20030661	6.5502503	5.74E-11	8.64E-09
ABO	130.285664	1.67677771	0.44008514	3.81012115	0.0001389	0.00204092
DBH	6.10748193	3.93486674	1.39463624	2.82142873	0.00478103	0.03049459
DBH-AS1	18.657759	2.60451171	0.85459531	3.04765503	0.00230635	0.01781397
SARDH	57.0625663	1.98678794	0.55081018	3.60702833	0.00030972	0.00386623
COL5A1	462.598344	1.1202229	0.26792191	4.18115446	2.90E-05	0.00059311
LHX3	16.8441277	5.06189701	1.05155023	4.81374722	1.48E-06	5.31E-05
MAMDC4	1310.11014	1.07349416	0.3033522	3.53877163	0.00040199	0.00470574
RNF208	112.244311	1.17098453	0.33802198	3.46422602	0.00053176	0.00591085
CYSRT1	97.1737187	1.25710764	0.39551644	3.17839539	0.00148093	0.0128077
EPS8L2	1609.4645	1.88072121	0.52878212	3.55670349	0.00037554	0.00445992
PIDD1	2086.32837	1.09378646	0.28873545	3.7881959	0.00015175	0.00218553
MUC6	24.192291	2.02010494	0.48596272	4.15691342	3.23E-05	0.00064515
IFITM10	8.66432487	3.41268864	0.87947052	3.88039002	0.00010429	0.00163711
TNNT3	9.99097199	3.69711961	0.81411056	4.54129915	5.59E-06	0.00015462
TRPM5	8.251234	2.19321198	0.71112054	3.08416346	0.00204125	0.01633303
SLC22A18	273.004362	1.01029958	0.37767311	2.67506359	0.00747151	0.04218067
UBQLNL	38.3108484	1.0119962	0.34340797	2.94692116	0.00320955	0.02272303
HPX	171.827516	2.29327137	0.4254353	5.39041156	7.03E-08	4.12E-06
RIC3	402.966268	1.05627973	0.33561481	3.1472977	0.00164787	0.0138232

ADM	1086.45985	1.30932077	0.27126926	4.8266463	1.39E-06	4.99E-05
AMPD3	110.844851	1.50067885	0.27668064	5.42386654	5.83E-08	3.53E-06
MTRNR2L8	29.2516104	1.17414718	0.36629717	3.2054498	0.00134852	0.01189835
GALNT18	23.6861454	1.99568917	0.58721588	3.39856129	0.00067741	0.00710291
USH1C	26.1322199	2.26905645	0.6402321	3.54411538	0.00039393	0.00463126
PTPN5	58.9665923	1.38286486	0.48874416	2.82942484	0.00466318	0.02991781
CSTF3-DT	44.8886471	1.08579867	0.30452907	3.56550091	0.00036316	0.00435635
ABTB2	564.583854	1.52025076	0.3009434	5.05161697	4.38E-07	1.91E-05
C11orf96	138.836831	2.50235168	0.40839838	6.12723219	8.94E-10	9.29E-08
CD82	115.004768	1.65945309	0.29948294	5.54106052	3.01E-08	1.98E-06
SYT13	39.2944459	1.26416264	0.43261983	2.92210983	0.00347669	0.02419446
LINC02696	38.2827565	1.10740262	0.42242459	2.62153921	0.00875337	0.04735892
CREB3L1	92.3214722	1.02390566	0.31519644	3.24846834	0.00116028	0.01066442
AGBL2	189.221194	1.31066322	0.26031463	5.03491954	4.78E-07	2.05E-05
TRIM51CP	20.6023775	1.63328409	0.59602722	2.74028441	0.0061386	0.03670714
TRIM51FP	26.8217012	2.29682751	0.72482255	3.16881356	0.00153063	0.0131541
TRIM48	175.613803	2.12322902	0.7612625	2.78908919	0.00528565	0.03286774
MS4A15	7.28934274	2.90626405	1.06370804	2.73220089	0.00629128	0.0373321
VWCE	594.803917	2.32417217	0.34834186	6.67210135	2.52E-11	4.23E-09
ROM1	143.889879	1.03845739	0.2394968	4.33599697	1.45E-05	0.00034684
AP003068.4	76.6818993	1.19407259	0.25615377	4.66154597	3.14E-06	9.66E-05
SLC22A20P	44.9467445	1.93991144	0.44282213	4.38079155	1.18E-05	0.00029221
SNX32	222.20503	1.05073511	0.26268316	4.00000941	6.33E-05	0.00108911
FOSL1	628.721422	1.98677735	0.29763888	6.675127	2.47E-11	4.21E-09
RHOD	799.47163	2.00490214	0.32535509	6.16219683	7.17E-10	7.53E-08
CARNS1	151.823107	1.78400461	0.48658025	3.66641395	0.00024598	0.00321389
CABP4	9.76885172	2.51320714	0.74057064	3.39360895	0.00068978	0.00720489
AP002807.1	18.0089165	2.09860419	0.74977245	2.79898812	0.0051263	0.03212404
AP002992.1	28.9335684	1.47064303	0.47772424	3.07843503	0.00208091	0.01657133
AP002813.1	11.2818126	1.96125351	0.62833027	3.12137361	0.00180009	0.01481632
SHANK2	49.3571552	2.41626829	0.52295629	4.62040201	3.83E-06	0.00011339
NADSYN1	2472.35709	1.17159991	0.22855271	5.12616953	2.96E-07	1.41E-05
OR7E128P	27.6283768	1.30988543	0.40824124	3.20860636	0.0013338	0.01180191
SYTL2	329.363844	1.1816472	0.19367928	6.10105121	1.05E-09	1.05E-07
PRSS23	281.918637	1.3250232	0.31788993	4.16818237	3.07E-05	0.00062033
AMOTL1	1023.24542	1.2501903	0.18408601	6.79133782	1.11E-11	2.07E-09
AP002383.3	375.809364	2.32052781	0.6012389	3.85957698	0.00011358	0.00174904
AP002383.4	18.5619076	2.12235267	0.73799331	2.87584271	0.0040295	0.02697049
ARHGAP42	347.292384	1.13882288	0.17969419	6.33756085	2.33E-10	2.81E-08

BIRC3	347.186724	1.7530208	0.57215825	3.06387403	0.00218491	0.01714301
MMP12	12.140826	3.47410625	1.04128847	3.33635334	0.00084885	0.00839998
C11orf87	25.7768725	1.53311158	0.57361029	2.67274073	0.00752344	0.04236607
ARHGAP20	37.2478975	1.53557825	0.3666873	4.18770503	2.82E-05	0.00057842
DSCAML1	68.9565284	3.89207257	1.35478441	2.87283536	0.00406806	0.02715359
IL10RA	22.8394536	2.29493867	0.71600054	3.2052192	0.0013496	0.01190307
SCN4B	538.042626	2.03121692	0.23478387	8.65143304	5.09E-18	2.85E-15
SCN3B	35.8067988	3.17751553	0.4681417	6.78750805	1.14E-11	2.08E-09
ESAM	54.4955096	1.25082248	0.30990689	4.03612351	5.43E-05	0.000958
FEZ1	1743.58704	1.39750838	0.21804556	6.40924936	1.46E-10	1.94E-08
TP53AIP1	20.201861	2.15954389	0.8255535	2.61587393	0.00889994	0.04789068
ITIH5	15.8736229	4.49909807	0.78767824	5.71184764	1.12E-08	8.44E-07
CCDC3	506.605316	1.51000346	0.32517256	4.64369887	3.42E-06	0.00010357
C1QL3	36.6350693	1.50072834	0.44628446	3.36271695	0.00077179	0.00781045
SPAG6	74.2003081	1.10713984	0.35473134	3.12106577	0.00180198	0.01481754
BAMBI	222.65426	1.76053191	0.37743539	4.66445904	3.09E-06	9.55E-05
JCAD	420.100216	1.88233299	0.35714246	5.27053827	1.36E-07	7.40E-06
AL450326.2	14.4849299	2.06162016	0.57803984	3.56657108	0.00036168	0.00434099
ZNF22-AS1	35.6984008	1.35932764	0.41723814	3.25791796	0.00112233	0.01041651
AC073174.1	760.318921	1.84979978	0.45443916	4.07051139	4.69E-05	0.00085943
GAPDHP21	6.26540523	3.66806201	1.13657991	3.22728035	0.00124973	0.01127754
FAM13C	6.73368213	3.09588851	0.91815788	3.37184767	0.00074666	0.00763452
EGR2	34.4007144	1.70889235	0.59583503	2.86806293	0.00412993	0.02744903
RPS3AP38	21.2316071	1.43680157	0.41618948	3.45227747	0.00055588	0.00613206
UNC5B-AS1	12.4103696	2.6797604	0.69303094	3.8667255	0.00011031	0.00171065
VSIR	56.463266	1.34052355	0.46989036	2.85284324	0.004333	0.02837516
AC073389.2	15.0885555	1.26191317	0.48083238	2.62443464	0.00867929	0.04705148
PLAU	415.931308	1.67500666	0.28920056	5.79185143	6.96E-09	5.61E-07
MBL1P	14.9929189	1.62761413	0.61049975	2.66603572	0.00767515	0.0429085
CDHR1	68.7544088	1.54910876	0.46412237	3.33771624	0.0008447	0.00837788
MMRN2	92.9277552	2.12125061	0.42741763	4.96294601	6.94E-07	2.81E-05
AC025268.1	28.073936	2.76945466	0.60723833	4.5607376	5.10E-06	0.00014316
ADIRF-AS1	7.38659705	2.60439761	0.92422328	2.8179312	0.00483342	0.03076266
CFL1P1	44.5252639	1.3761287	0.34084207	4.03743793	5.40E-05	0.00095552
KLLN	179.56551	1.24672271	0.22606601	5.5148613	3.49E-08	2.26E-06
ACTA2	299.770624	2.65343192	0.63639292	4.16948684	3.05E-05	0.00061787
AL157394.3	17.12933	3.06399937	0.62027288	4.93976032	7.82E-07	3.11E-05
FAS	585.628672	2.11504543	0.24259117	8.71855917	2.82E-18	1.81E-15
PPP1R3C	329.882511	1.59103547	0.25292893	6.2904449	3.17E-10	3.64E-08

CYP2C19	21.123651	2.92013147	0.92179438	3.16787729	0.00153556	0.01318096
CYP2C9	46.7286336	3.05528097	0.72338935	4.22356366	2.40E-05	0.00051291
MTND4P20	16.9196513	2.15281399	0.77148743	2.79047188	0.00526313	0.03276503
TLL2	111.414154	1.30914858	0.24940968	5.24898856	1.53E-07	8.13E-06
CRTAC1	156.572318	2.65697222	0.44609939	5.95600954	2.58E-09	2.32E-07
LOXL4	145.837137	1.65700538	0.27260414	6.07843067	1.21E-09	1.19E-07
ELOVL3	56.5074989	1.46992298	0.38079897	3.86010229	0.00011334	0.00174652
AL121929.3	83.4644181	1.86333892	0.4241071	4.39355743	1.12E-05	0.00027871
PDCD4-AS1	119.664434	1.1006209	0.2972725	3.70239734	0.00021357	0.00289449
HSPA12A	1137.2511	1.01499584	0.17540692	5.78652112	7.19E-09	5.76E-07
CPXM2	50.0670635	2.21658531	0.61233338	3.61989952	0.00029472	0.00373012
CHST15	79.69597	1.58197796	0.50906422	3.1076196	0.00188601	0.01538993
NKX1-2	45.5398944	3.77387118	0.45151448	8.35825057	6.37E-17	3.02E-14
FANK1	55.6306471	1.22643382	0.420708	2.9151664	0.00355499	0.02460973
ADAM12	76.7653629	1.07627718	0.36498636	2.94881477	0.00318995	0.02263569
INSYN2A	191.992171	2.72486211	0.21593576	12.6188554	1.66E-36	6.04E-33
TCERG1L	59.6975698	1.51255029	0.52298824	2.8921306	0.00382639	0.02595331
FRG2B	16.157271	1.93487268	0.7114173	2.71974364	0.00653325	0.03843961
CACNA1C	30.6951727	1.14608032	0.44070477	2.60056252	0.00930711	0.04961609
AC005865.1	18.4517016	2.80585933	0.57171013	4.90783557	9.21E-07	3.55E-05
CD27-AS1	172.852784	1.35881266	0.36112804	3.7626894	0.0001681	0.00237709
C1R	56.3778804	1.55968629	0.31524739	4.94749944	7.52E-07	3.02E-05
POU5F1P3	11.2323374	1.86562621	0.58654054	3.18072848	0.00146905	0.01272518
AC092111.1	32.3140704	1.59885058	0.49606706	3.22305331	0.00126832	0.01137006
LINC00937	13.0139173	2.24175207	0.62629026	3.57941393	0.00034437	0.00419545
MFAP5	71.1249733	2.02339529	0.4950706	4.08708434	4.37E-05	0.00081676
KLRD1	95.5750907	1.41859319	0.40210777	3.52789302	0.00041888	0.00487987
GPRC5A	197.128126	2.31836696	0.42191841	5.49482292	3.91E-08	2.47E-06
AC137561.1	25.1377824	1.77938342	0.49599384	3.58751112	0.00033385	0.00409707
PTHLH	2294.50922	1.73348347	0.66106859	2.62224449	0.00873527	0.04727274
AC010198.2	97.4473208	1.11997057	0.42596498	2.62925504	0.00855722	0.04650522
TSPAN11	463.531114	1.51842525	0.32808258	4.62817999	3.69E-06	0.00011011
MUC19	16.7028931	1.80600087	0.63237305	2.85591055	0.00429136	0.02822893
AC006197.2	16.5449189	1.56051213	0.44689306	3.49191404	0.00047957	0.00544193
AC004241.2	6.73765635	3.28377547	1.03668111	3.16758493	0.00153711	0.0131841
VDR	15.3010833	2.38524124	0.57618785	4.13969373	3.48E-05	0.0006824
RND1	63.0325122	1.02162155	0.29710543	3.43858254	0.00058477	0.00636074
ITGB7	12.1719088	1.70566453	0.64327089	2.65154939	0.00801234	0.04427126
HOXC13	516.330326	1.29982118	0.22829032	5.69372013	1.24E-08	9.23E-07

GPR84	7.20299977	4.59879867	1.03963968	4.42345436	9.71E-06	0.00024881
DGKA	1964.22498	1.04822005	0.20894406	5.01674976	5.26E-07	2.21E-05
PMEL	85.9303084	1.40748614	0.45134109	3.1184534	0.00181803	0.01492624
GLS2	40.3274451	1.9760279	0.45243435	4.36754617	1.26E-05	0.00030702
TAC3	63.5706937	1.81723323	0.58275056	3.11837235	0.00181853	0.01492624
MDM2	13577.7328	1.4226731	0.19222091	7.40123997	1.35E-13	3.46E-11
CPM	103.573759	2.70187594	0.54256438	4.97982546	6.36E-07	2.62E-05
CAPS2	145.844309	1.27313731	0.41455878	3.07106585	0.00213296	0.01683226
PTPRQ	13.6122897	1.62988775	0.56688156	2.87518215	0.00403794	0.02701869
BTG1	580.12599	1.03829855	0.25074843	4.14079776	3.46E-05	0.00067997
CCDC38	26.1583236	1.23184646	0.40990132	3.00522684	0.00265383	0.01975616
DRAM1	3039.89431	1.93268751	0.22308683	8.66338674	4.58E-18	2.70E-15
C12orf45	2416.53633	1.08472332	0.12871649	8.42722913	3.54E-17	1.72E-14
TCP11L2	94.4490148	1.30162253	0.26498364	4.91208647	9.01E-07	3.50E-05
ISCU	5113.84048	1.11268428	0.11895584	9.35375879	8.46E-21	7.10E-18
TRPV4	156.45031	1.46150609	0.32198792	4.53900905	5.65E-06	0.00015532
RASAL1	52.9621814	2.838018	0.48244631	5.88255717	4.04E-09	3.42E-07
AC089999.4	18.9179098	1.38321297	0.42000289	3.29334157	0.00099004	0.00947487
RNU4-2	8.56432264	2.31823202	0.86485926	2.68047315	0.00735182	0.04175342
SIRT4	96.403459	1.21851746	0.24051766	5.06622865	4.06E-07	1.80E-05
TRIAP1	4516.41291	1.15645757	0.13535497	8.5438867	1.30E-17	6.74E-15
MORN3	89.113224	1.55078478	0.2255691	6.8749877	6.20E-12	1.19E-09
TMEM132B	133.952241	1.14919703	0.38489438	2.98574644	0.00282887	0.02076218
RIMBP2	47.2167142	3.0048259	0.65713901	4.57258792	4.82E-06	0.00013723
AC131009.1	17.3306667	1.13933212	0.41276003	2.76027723	0.00577523	0.03506277
GJA3	397.743417	1.10695387	0.31973633	3.46208349	0.00053601	0.00593995
ATP8A2	21.4323341	3.7210882	0.65431744	5.68697692	1.29E-08	9.56E-07
RASL11A	145.051382	1.46045963	0.27566732	5.29790637	1.17E-07	6.52E-06
CDX2	52.9679569	1.26574326	0.44437667	2.84835668	0.00439456	0.0286237
N4BP2L1	176.073768	1.50166496	0.20858836	7.19917912	6.06E-13	1.39E-10
MAB21L1	11.5340515	2.17136753	0.79420568	2.73401157	0.00625678	0.03722002
DCLK1	48.7307312	2.60726733	0.98118025	2.65727663	0.00787748	0.04371479
RGCC	98.8836712	1.20998281	0.45879361	2.63731398	0.00835654	0.04572212
LACC1	405.015762	1.96166092	0.32052104	6.12022508	9.34E-10	9.66E-08
SMIM2-AS1	17.7033948	1.65190261	0.60890527	2.71290577	0.00666961	0.0389954
AL138960.1	14.6968756	1.42857977	0.52213453	2.73603773	0.00621839	0.03703833
OGFOD1P1	6.95642513	2.36837118	0.83990164	2.81981968	0.00480506	0.03063
THSD1	38.3665247	1.33434508	0.47884169	2.78661009	0.00532625	0.03301761
HS6ST3	16.7743845	2.01639869	0.49424123	4.0797865	4.51E-05	0.00083636

DOCK9-DT	16.0129174	1.14146041	0.42887444	2.66152585	0.00777874	0.04332109
FAM155A	64.4704315	1.83680103	0.40899524	4.49100838	7.09E-06	0.0001884
AL139384.2	55.9748362	1.60580124	0.33045807	4.85931914	1.18E-06	4.33E-05
F10	20.7301479	1.10352101	0.4060483	2.71770875	0.00657357	0.03864096
AL137002.2	35.2324525	1.17001707	0.38221975	3.06111097	0.00220517	0.01727096
GAS6	859.597615	1.46365573	0.30758054	4.75860965	1.95E-06	6.58E-05
GAS6-DT	8.03495906	4.19408501	1.01170399	4.14556533	3.39E-05	0.00066936
PNP	6696.65944	1.39099842	0.32082286	4.33572103	1.45E-05	0.00034684
TMEM253	17.0030937	1.51529	0.44970736	3.36950233	0.00075304	0.00768179
ABHD4	1368.3052	1.52170622	0.23241121	6.54747333	5.85E-11	8.75E-09
JPH4	39.1290217	1.939286	0.45330727	4.27808273	1.89E-05	0.00042669
TGM1	106.737654	1.80406476	0.29995896	6.01437195	1.81E-09	1.73E-07
AL121594.1	17.7086057	1.6398964	0.40567358	4.04240376	5.29E-05	0.00094238
AL162311.3	14.0473526	2.21981439	0.52397684	4.23647423	2.27E-05	0.00049331
DACT1	33.9402862	1.66929275	0.42250733	3.95092018	7.79E-05	0.00128853
AL157756.1	15.1150754	1.90667142	0.45386278	4.20098652	2.66E-05	0.00055385
TMEM229B	71.8987048	1.13302816	0.28012695	4.04469525	5.24E-05	0.00093703
SUSD6	1694.0471	1.12666872	0.15743241	7.15652314	8.27E-13	1.84E-10
SLC8A3	17.5703467	4.40840669	0.904305	4.87491133	1.09E-06	4.08E-05
AC005520.4	34.8355226	1.70852025	0.33323482	5.12707603	2.94E-07	1.41E-05
PROX2	24.1017221	1.20315013	0.42824279	2.80950472	0.00496178	0.03136747
PGF	378.337285	1.37370464	0.22540138	6.09448186	1.10E-09	1.09E-07
FOS	499.407252	1.80474723	0.39358236	4.58543726	4.53E-06	0.00013058
ESRRB	21.1241926	1.60322071	0.39502984	4.05847999	4.94E-05	0.00089629
DIO2	19.280923	1.69534821	0.63361926	2.67565765	0.00745828	0.04213517
KCNK10	5.4539071	5.58601144	1.34897083	4.14094308	3.46E-05	0.00067997
SPATA7	312.39808	1.08176756	0.16270444	6.64866643	2.96E-11	4.82E-09
FBLN5	93.9933946	1.46659441	0.47544862	3.08465381	0.00203789	0.01633225
SLC24A4	28.5738723	1.48627073	0.44841586	3.31449184	0.0009181	0.00894726
CHGA	104.502721	1.05973123	0.30211241	3.50773817	0.00045193	0.0051792
TUNAR	34.1290286	1.47357622	0.49593362	2.97131748	0.00296525	0.02151792
AL137786.1	75.3245031	1.02722136	0.25613094	4.01053212	6.06E-05	0.00104996
HHIPL1	122.223391	1.15426756	0.20004353	5.77008189	7.92E-09	6.24E-07
DEGS2	23.6433731	2.21929328	0.63122077	3.51587492	0.00043831	0.0050549
MEG3	2689.58597	2.36078883	0.36699405	6.43277141	1.25E-10	1.72E-08
MEG8	30.4548457	1.87581425	0.72092187	2.60196606	0.0092691	0.04948433
MEG9	34.7500505	2.1893219	0.71663215	3.0550149	0.00225049	0.01748781
AMN	26.8916124	1.52551848	0.42435343	3.59492438	0.00032449	0.00401149
ADSS1	580.340547	1.37747965	0.30204949	4.56044349	5.10E-06	0.00014316

AHNAK2	727.476739	1.38090561	0.27160726	5.08419984	3.69E-07	1.68E-05
LINC02298	129.303987	1.49231224	0.29988742	4.97624151	6.48E-07	2.65E-05
JAG2	1000.0722	1.27097498	0.32490013	3.91189438	9.16E-05	0.00147357
CHEK2P2	21.8308086	2.17524953	0.66831703	3.25481683	0.00113466	0.01048631
NBEAP1	15.8669891	2.11200707	0.77763391	2.71594003	0.00660879	0.03876446
FAM189A1	153.606662	1.06339318	0.31032697	3.42668629	0.00061099	0.00656097
GOLGA8R	18.6806604	1.72596149	0.56655981	3.04638886	0.00231608	0.01787019
CHRNA7	118.809537	1.37431762	0.20652081	6.65462051	2.84E-11	4.66E-09
SCG5	84.9902114	1.04073661	0.31451395	3.30903164	0.00093619	0.0090883
AC051619.4	41.3329419	1.41634148	0.38332889	3.69484674	0.00022002	0.0029653
AC022306.1	8.44960444	2.0276168	0.73390759	2.76276852	0.00573134	0.03484476
IGDCC4	1325.77306	1.14638622	0.19141334	5.98906122	2.11E-09	1.99E-07
AC087639.1	9.38897428	2.61167412	0.73415712	3.55737766	0.00037458	0.00445165
GRAMD2A	110.811729	1.40790634	0.28964227	4.8608455	1.17E-06	4.31E-05
INSYN1-AS1	18.6516554	2.38300528	0.64875627	3.67319038	0.00023954	0.00314677
AC104758.2	20.0642628	2.28389266	0.72712901	3.1409731	0.00168387	0.01405054
ADAMTS7	1325.58493	2.37651304	0.27198623	8.73762271	2.38E-18	1.57E-15
CTXND1	55.9584506	1.85692495	0.39346221	4.71944925	2.36E-06	7.69E-05
ADAMTS7P1	18.0423209	2.1450091	0.52004892	4.12462947	3.71E-05	0.0007215
AP3B2	123.240497	1.09279997	0.22463714	4.86473407	1.15E-06	4.25E-05
ACTG1P17	23.4300309	1.43355781	0.43411377	3.30226293	0.00095908	0.0092639
TM6SF1	20.0154444	1.87251298	0.45359172	4.12819036	3.66E-05	0.00071105
BNC1	14.6548735	2.29832024	0.8339132	2.7560665	0.00585011	0.03537955
NMB	383.784165	1.3160975	0.29983356	4.38942694	1.14E-05	0.00028309
ISG20	104.329777	1.19392498	0.34017444	3.50974334	0.00044854	0.00514841
RGMA	329.502562	2.29955155	0.32875675	6.99469003	2.66E-12	5.32E-10
LINC02253	25.4584879	1.68440668	0.59954783	2.80946169	0.00496244	0.03136747
ARRDC4	307.829356	1.10615664	0.31437508	3.51858885	0.00043385	0.00501939
AC090825.1	228.072851	1.3258744	0.18255826	7.26274675	3.79E-13	9.20E-11
AC027020.2	66.6448992	1.49471518	0.25261497	5.91696997	3.28E-09	2.86E-07
ALDH1A3	56.3800402	1.64207415	0.4248356	3.86519905	0.000111	0.00171763
Z92544.1	117.414581	1.34502082	0.24987793	5.38271154	7.34E-08	4.29E-06
Z97653.1	6.93669933	4.08706924	0.99614096	4.1029025	4.08E-05	0.00077616
AC106820.4	68.1850514	1.03511645	0.32796451	3.15618431	0.00159848	0.0135399
PRSS27	74.8825729	1.01604457	0.3758531	2.70330238	0.00686543	0.03979904
CLDN9	21.3778556	1.64492448	0.60478313	2.71985842	0.00653099	0.03843961
VASN	251.475125	1.19040531	0.32514081	3.66119929	0.00025104	0.00326828
SOCS1	39.9630459	1.73514648	0.46233445	3.75301146	0.00017472	0.00245648
AC099489.1	40.8031852	1.42846906	0.48471243	2.94704442	0.00320827	0.02272135

NPIPB2	38.5664228	1.85843285	0.36378489	5.10860375	3.25E-07	1.51E-05
MIR193BHG	171.884803	1.21668019	0.26648081	4.56573284	4.98E-06	0.00014089
PLA2G10	8.91623499	2.48915996	0.78953143	3.15270532	0.00161765	0.01363447
AC138932.6	8.15738075	2.04394505	0.5947574	3.43660298	0.00058906	0.00638512
AC005632.3	26.7780671	1.27948296	0.44518761	2.87403094	0.00405269	0.02707586
AC109449.1	14.478331	1.54785293	0.43064583	3.59425968	0.00032532	0.00401719
IL4R	711.013673	1.06856365	0.16519548	6.46847983	9.90E-11	1.38E-08
YPEL3	187.640449	1.76961422	0.27416341	6.45459665	1.09E-10	1.50E-08
ITGAL	24.0848484	2.41623411	0.76030792	3.17796784	0.00148311	0.01281644
ORAI3	464.500595	1.18188961	0.30097115	3.92691991	8.60E-05	0.0014021
AC026471.4	57.0194661	1.00303471	0.32615701	3.07531243	0.00210282	0.01669708
SLC6A10P	341.564752	1.28548305	0.19407129	6.62376705	3.50E-11	5.54E-09
AC133561.1	68.3851482	1.03051198	0.27194083	3.78947134	0.00015097	0.00217578
ABCC11	23.0217629	1.00412508	0.37079043	2.70806634	0.00676765	0.03933228
CDH8	144.082387	1.52662274	0.42086216	3.62736994	0.00028632	0.00364502
CA7	30.1176379	1.80184011	0.4955352	3.63614955	0.00027674	0.00353752
RRAD	220.316712	2.84088437	0.69161085	4.1076342	4.00E-05	0.00076473
DPEP3	34.3963193	1.83493405	0.66761042	2.74851021	0.00598668	0.03596621
CDH3	124.250723	2.68338032	0.27198634	9.86586444	5.85E-23	6.08E-20
HAS3	443.654534	1.19381827	0.23555032	5.06820904	4.02E-07	1.79E-05
TMEM231P1	10.5314802	1.70928762	0.55118677	3.10110423	0.001928	0.01565651
DNAAF1	28.5585492	1.88783831	0.53408473	3.5347169	0.00040821	0.00477086
CRISPLD2	236.027649	3.33775007	0.46883089	7.11930489	1.08E-12	2.34E-10
FAM92B	32.9895172	1.47907443	0.45029711	3.28466341	0.00102104	0.00970769
ZNF469	162.339514	2.70228711	0.32427333	8.33336223	7.86E-17	3.65E-14
LINC00304	12.3953375	2.22188062	0.66936857	3.31936802	0.00090221	0.00881609
SCARF1	31.4711488	2.05886581	0.61878106	3.32729286	0.00087694	0.00861931
TRPV3	62.7708977	1.22682419	0.25772679	4.76017322	1.93E-06	6.56E-05
AC132942.1	25.7909802	1.32204698	0.36989454	3.57411868	0.00035141	0.00425514
ATP2A3	274.583593	1.05890634	0.39094272	2.70859719	0.00675683	0.03931575
SMTNL2	30.0839918	1.28785088	0.39420759	3.26693581	0.00108718	0.01016381
SLC52A1	1061.8736	3.71761852	0.56514551	6.57816165	4.76E-11	7.32E-09
AC055839.2	453.147107	1.05123911	0.26724195	3.9336606	8.37E-05	0.00137256
ASGR1	191.444851	1.42421732	0.31417744	4.53316229	5.81E-06	0.00015908
SHBG	32.7127101	1.73846893	0.42747731	4.06680988	4.77E-05	0.00086954
SAT2	2423.22822	1.07739798	0.16583123	6.49695449	8.20E-11	1.18E-08
ATP1B2	62.3161863	1.26191829	0.37701199	3.3471569	0.00081645	0.00815329
LINC00324	120.316159	2.39719364	0.3189885	7.51498446	5.69E-14	1.55E-11
NTN1	12354.6318	2.22699082	0.62485487	3.56401291	0.00036523	0.00436912

AC005291.2	25.1091347	1.73214585	0.53975193	3.20915175	0.00133127	0.01178911
AC005291.1	22.7481896	1.79312511	0.59803395	2.99836676	0.00271431	0.02009712
HS3ST3B1	153.373494	1.34874872	0.47910155	2.81516248	0.00487526	0.03091489
TVP23C	145.478058	1.13510156	0.29054785	3.90676284	9.35E-05	0.00149921
RASD1	211.194769	1.58514256	0.33199213	4.77463893	1.80E-06	6.17E-05
MYO15A	53.6122907	1.33932236	0.31524487	4.24851433	2.15E-05	0.00047382
NOS2P2	14.0464438	1.96661374	0.74017306	2.65696478	0.00788477	0.043733
AC107982.3	12.687135	1.35173344	0.4853298	2.78518532	0.00534971	0.03314332
SNORD3A	44.1340063	1.95272291	0.53043503	3.68136114	0.00023199	0.0030753
ALDH3A1	21.7729664	1.3520895	0.48517404	2.78681337	0.00532291	0.03301761
NATD1	881.461502	1.09051758	0.22735327	4.7965774	1.61E-06	5.65E-05
MTRNR2L1	148.023234	1.51362236	0.42260907	3.58161354	0.00034148	0.00416493
LYRM9	189.327146	1.092777	0.27483364	3.97613991	7.00E-05	0.00118201
SLC6A4	39.5741752	1.49606947	0.41858156	3.57414091	0.00035138	0.00425514
AC138207.5	20.6366859	2.07784186	0.49128746	4.22938102	2.34E-05	0.00050426
AC138207.2	29.9596787	1.05817832	0.36811809	2.87456217	0.00404588	0.02704691
OMG	13.7335373	1.22119153	0.44576446	2.73954438	0.00615244	0.03676972
AC004253.1	43.178599	1.2758054	0.35944571	3.54936878	0.00038616	0.00455455
AC116407.2	40.6008502	1.43801005	0.44813074	3.20890738	0.0013324	0.01179434
SLFN5	282.341855	2.09274495	0.62870453	3.32866212	0.00087264	0.00859184
CCL3L1	6.31781251	2.49619076	0.80935278	3.08418137	0.00204113	0.01633303
LRRC37A11P	15.8255821	1.57545302	0.48696947	3.23521928	0.00121549	0.01102789
PLXDC1	391.896912	1.75297937	0.35689355	4.91177093	9.03E-07	3.50E-05
GJD3	40.6434669	1.10216278	0.37088175	2.97173635	0.00296121	0.02150202
KRT19	21.1332351	1.87214947	0.52935112	3.53668748	0.00040518	0.00473794
HAP1	408.773024	1.30312281	0.37082643	3.51410444	0.00044124	0.00508065
AOC2	78.6789092	1.01342666	0.34692428	2.92117534	0.00348714	0.02423998
LINC00910	327.821361	1.10751216	0.31185807	3.55133404	0.00038328	0.00453538
MAP3K14-AS1	497.79093	1.11816516	0.25749345	4.34249947	1.41E-05	0.0003389
TBX21	133.796338	1.97752704	0.42254768	4.68000917	2.87E-06	8.94E-05
LINC02086	128.0956	2.3471666	0.41179242	5.69987809	1.20E-08	8.98E-07
HOXB13	201.16479	1.11654029	0.29042821	3.84446226	0.00012082	0.00183071
TTLL6	51.4306301	2.33621593	0.49197862	4.74861274	2.05E-06	6.86E-05
PHOSPHO1	90.3411539	1.13180132	0.38225799	2.960831	0.0030681	0.02202896
MYCBPAP	172.041271	1.38811056	0.25211397	5.50588525	3.67E-08	2.34E-06
AC021491.2	64.6575573	1.67204029	0.34150476	4.89609653	9.78E-07	3.76E-05
EPN3	112.507053	1.37405114	0.34434088	3.99038055	6.60E-05	0.00112103
ABCC3	12.9010672	1.8925543	0.70898672	2.66937905	0.00759916	0.0426366
WFIKKN2	17.3469649	2.40043795	0.86413375	2.77785464	0.00547191	0.03367091

NOG	91.7578875	1.9513097	0.42949902	4.5432227	5.54E-06	0.00015353
AC007114.1	40.0236516	1.04187874	0.28166686	3.69897517	0.00021647	0.0029211
TEX14	102.101195	1.46843006	0.47143862	3.11478523	0.00184079	0.01507733
PPM1D	3137.3039	1.01910695	0.12244073	8.32326738	8.56E-17	3.89E-14
LINC02875	81.6212775	1.00131432	0.35902402	2.78898977	0.00528727	0.03286846
AC132812.1	45.7275807	1.25619985	0.31660727	3.96769116	7.26E-05	0.00121742
ABCA9	1042.80926	1.21813082	0.34776484	3.50274289	0.00046049	0.00526347
ABCA6	380.020016	1.83278926	0.61871042	2.96227315	0.00305377	0.02194047
GRIN2C	496.558334	2.8179887	0.38527125	7.31429787	2.59E-13	6.56E-11
FDXR	5706.38259	1.76192074	0.27755496	6.34800674	2.18E-10	2.64E-08
MYO15B	1235.90719	1.48875733	0.29107163	5.11474549	3.14E-07	1.47E-05
ST6GALNAC2	20.5634716	1.57256637	0.58970893	2.66668229	0.00766041	0.04285694
AC022966.2	12.1080498	1.66289466	0.47419622	3.50676488	0.00045359	0.00519272
AC087741.1	201.434911	1.05632617	0.2757862	3.83023578	0.00012802	0.00190546
CD7	142.488706	1.52611129	0.42360095	3.60270979	0.00031492	0.00391538
SECTM1	62.6928602	2.24030323	0.6172026	3.62976959	0.00028367	0.00361764
METRNL	177.313114	1.33054698	0.26186158	5.08110803	3.75E-07	1.70E-05
TUBB8B	113.952411	1.00648197	0.27057092	3.7198453	0.00019934	0.00273642
AP001005.3	48.931228	1.13869609	0.31905877	3.56892275	0.00035845	0.00431385
AP001178.4	102.912466	1.79510385	0.21059345	8.52402521	1.54E-17	7.82E-15
AP001178.3	73.5630583	1.16973681	0.36540519	3.20120472	0.00136854	0.01203611
AP000919.1	14.34221	2.10853623	0.6630349	3.18012856	0.0014721	0.01274649
MYOM1	671.096759	1.29190646	0.30894227	4.18170828	2.89E-05	0.00059278
PTPRM	58.2889523	1.0342313	0.39007103	2.65139223	0.00801607	0.04427126
ANKRD29	100.214021	1.69014203	0.51596886	3.27566676	0.00105413	0.00993997
CELF4	75.3500606	1.06509991	0.26834739	3.96910844	7.21E-05	0.00121274
AC021504.1	96.7925091	1.26479976	0.43338385	2.91842844	0.00351801	0.024396
PSTPIP2	711.974065	1.33458822	0.18510731	7.20980815	5.60E-13	1.30E-10
SMAD7	256.232322	1.47436666	0.23176447	6.36148708	2.00E-10	2.49E-08
LINC01630	248.934378	1.12287132	0.30508833	3.6804794	0.0002328	0.00308193
CCDC68	9.17338814	1.81891245	0.57573978	3.1592614	0.0015817	0.01343425
PMAIP1	62.4152413	3.14106266	0.38218823	8.21862731	2.06E-16	8.98E-14
CD226	17.8210909	1.84633528	0.6472587	2.8525461	0.00433705	0.02839318
FBXO15	377.713659	1.09650647	0.32250726	3.39994348	0.000674	0.0070739
SALL3	44.6716838	1.58918094	0.58951284	2.69575288	0.00702297	0.04046509
AC139100.2	48.2805043	1.24086319	0.26952151	4.60394863	4.15E-06	0.00012125
AC139100.1	74.4373481	2.00171266	0.36704962	5.45352064	4.94E-08	3.04E-06
AL034548.2	51.9884425	1.43288317	0.28483022	5.03065704	4.89E-07	2.09E-05
EBF4	266.390596	1.06992634	0.32924487	3.24963714	0.00115552	0.01062965

AL109976.1	417.25225	1.25653743	0.24584773	5.11103946	3.20E-07	1.50E-05
PRNP	1806.91287	1.24399049	0.15256169	8.1540162	3.52E-16	1.40E-13
CHGB	72.8584665	1.1651035	0.40795032	2.85599355	0.00429024	0.02822893
PLCB4	383.73203	1.00731443	0.33156968	3.03801726	0.0023814	0.01823236
AL133325.3	70.6548096	2.26900321	0.3646276	6.22279612	4.88E-10	5.28E-08
PPIAP2	5.36902146	2.81831339	0.98088161	2.87324522	0.00406279	0.02712668
DUX4L34	7.36157349	2.92318414	1.01782079	2.87200279	0.00407879	0.02720858
FRG2EP	16.824058	2.49543383	0.89663613	2.78310648	0.00538411	0.03328084
ANKRD20A21P	46.3054794	1.80284718	0.39561346	4.55709262	5.19E-06	0.00014491
FOXS1	25.0258978	2.69702464	0.68521706	3.93601499	8.28E-05	0.00136224
DUSP15	134.096895	1.07037793	0.26538043	4.03337169	5.50E-05	0.00096673
HCK	68.0380804	1.88591111	0.44796494	4.20995243	2.55E-05	0.00053901
MAP1LC3A	583.524486	1.04875731	0.28235572	3.71431228	0.00020376	0.00278396
PROCR	707.506523	2.02115498	0.25922167	7.79701403	6.34E-15	2.20E-12
AL121753.2	75.3948307	1.6580041	0.41588695	3.98667021	6.70E-05	0.00113693
MAFB	17.5175129	3.21568784	0.63602549	5.05591036	4.28E-07	1.89E-05
LPIN3	809.80406	1.79058971	0.53450421	3.35000111	0.00080811	0.00810153
SGK2	24.3577479	1.58117286	0.46290391	3.41576905	0.00063602	0.00675148
WFDC2	14.9174913	2.63743606	0.62328743	4.23149246	2.32E-05	0.00050153
ZSWIM3	129.273928	1.0720303	0.18578236	5.77035574	7.91E-09	6.24E-07
NEURL2	37.5935025	1.54995474	0.46949853	3.30129839	0.00096238	0.00927528
AL008726.1	11.3910743	3.02138105	0.93532883	3.23028751	0.00123666	0.01118734
PLTP	3304.14274	1.03168171	0.32246295	3.19938066	0.00137723	0.01208817
SULF2	4514.0881	1.96220533	0.20305928	9.6632143	4.32E-22	3.93E-19
AL354813.1	7.3121121	1.94461659	0.71417519	2.72288456	0.00647147	0.03815385
CEBPB-AS1	127.903364	1.6595918	0.36217378	4.58230798	4.60E-06	0.00013203
DOK5	17.0700898	1.97076429	0.62887328	3.1338019	0.00172557	0.01431091
AL109840.2	18.4633442	1.37712718	0.49238045	2.79687624	0.00515993	0.03224217
NKAIN4	13.8372354	1.93835212	0.74538165	2.60048275	0.00930927	0.04961609
COL20A1	17.5542349	1.55029206	0.57247854	2.70803523	0.00676828	0.03933228
PRSS57	6.71488113	3.45475692	1.02456774	3.37191653	0.00074647	0.00763452
AZU1	74.7124281	1.20244758	0.44408734	2.70768262	0.00677548	0.03935079
GRIN3B	97.2161648	1.72119481	0.54422127	3.16267463	0.00156327	0.01331403
PLK5	47.1888108	2.06094762	0.59147694	3.48440909	0.00049322	0.00556472
AC006130.3	15.2789955	1.3546635	0.48165664	2.81250869	0.00491567	0.03111689
EBI3	144.333688	1.67081064	0.41606948	4.01570107	5.93E-05	0.00103143
PLIN5	79.6419813	1.45302396	0.36686613	3.96063806	7.47E-05	0.00124602
TINCR	774.403541	1.52069911	0.25799517	5.89429282	3.76E-09	3.19E-07
FUT3	10.8375668	2.62473072	1.00259136	2.61794668	0.00884606	0.04769269

CAPS	100.908392	1.16013553	0.44109528	2.63012457	0.00853536	0.04642112
TNFSF9	139.909551	1.81779557	0.42842607	4.24296205	2.21E-05	0.00048384
KANK3	383.140099	1.36533537	0.41718958	3.27269769	0.00106526	0.01001899
C19orf38	21.2060739	1.58077506	0.58170853	2.71746928	0.00657833	0.03865852
AC010422.4	8.5136735	2.39478873	0.62427166	3.8361324	0.00012499	0.00187696
PODNL1	76.522062	1.35344008	0.47020485	2.87840516	0.00399691	0.02681783
AC008397.2	199.59228	5.46689709	0.40521715	13.4912778	1.76E-41	7.68E-38
PDE4C	122.003436	3.76462182	0.46023024	8.17986623	2.84E-16	1.15E-13
IQCN	264.930717	1.02284576	0.38171473	2.67960778	0.00737085	0.04182884
GDF15	2686.15563	3.65295342	0.31854588	11.4675895	1.92E-30	2.79E-27
NCAN	139.879507	1.22606104	0.38513993	3.18341713	0.00145548	0.0126528
TM6SF2	62.8753829	1.15677841	0.29929283	3.86503881	0.00011107	0.00171763
LINC00663	194.904333	1.67043232	0.26098893	6.40039538	1.55E-10	2.01E-08
ZNF56	118.607197	1.18362197	0.19916805	5.94283067	2.80E-09	2.49E-07
AC022432.1	24.645801	1.43098491	0.3775986	3.7896986	0.00015083	0.00217522
AC010300.1	70.1499217	1.24073775	0.34446934	3.6018815	0.00031592	0.00392118
SBSN	10.334031	4.37376354	0.87474675	5.00003405	5.73E-07	2.38E-05
TYROBP	33.4271649	2.19416497	0.52942936	4.14439609	3.41E-05	0.00067218
ZNF383	631.254281	1.02997919	0.16222615	6.34903295	2.17E-10	2.64E-08
ZNF540	229.510634	1.54201184	0.14888562	10.3570236	3.89E-25	4.47E-22
WDR87	58.3877347	1.2466223	0.40431036	3.0833301	0.00204698	0.01636084
KCNK6	628.982329	1.01153766	0.24585325	4.11439611	3.88E-05	0.00074761
CATSPERG	359.165016	1.36080644	0.32215741	4.22404199	2.40E-05	0.00051239
FAM98C	1125.57005	1.2090832	0.24143309	5.00794315	5.50E-07	2.30E-05
CAPN12	63.3875136	1.16496993	0.41090964	2.83510003	0.00458113	0.02950677
RINL	259.606993	1.10261224	0.19665804	5.60674885	2.06E-08	1.42E-06
AC007842.1	185.434172	1.32042857	0.21682817	6.08974634	1.13E-09	1.12E-07
TMEM91	172.912891	1.44160781	0.24105971	5.98029346	2.23E-09	2.06E-07
CEACAM1	41.8452722	1.54464315	0.46438062	3.32624377	0.00088025	0.00864403
LYPD5	30.4413526	2.46046361	0.38552184	6.38216394	1.75E-10	2.23E-08
ZNF404	594.755206	1.22918975	0.14978552	8.2063322	2.28E-16	9.61E-14
ZNF223	62.3674725	1.11778726	0.25041623	4.46371725	8.05E-06	0.00021227
APOE	4388.13082	1.19934363	0.32225198	3.72175718	0.00019784	0.00272531
RELB	566.371308	1.03858709	0.31603034	3.28635249	0.00101494	0.00966229
FOSB	186.340929	2.20338686	0.34319016	6.42030902	1.36E-10	1.82E-08
HIF3A	37.4120066	1.61492889	0.53686913	3.00804948	0.0026293	0.01962114
BBC3	1203.61915	1.47837365	0.26875447	5.50083373	3.78E-08	2.39E-06
PLA2G4C	110.369643	1.35497541	0.28528567	4.74953893	2.04E-06	6.84E-05
NTN5	21.1547674	2.31474856	0.66765533	3.46698134	0.00052634	0.00585954

FUT2	65.8100715	1.07599344	0.35030261	3.07161122	0.00212907	0.01681577
AC026803.3	31.8334219	1.18631341	0.32814565	3.6152038	0.00030011	0.00378085
DHDH	22.4250112	1.07001322	0.36114933	2.96279995	0.00304855	0.02191579
CGB7	54.7383618	1.48255623	0.40589625	3.65254969	0.00025965	0.00336634
KCNA7	102.434313	1.57655166	0.3946013	3.99530279	6.46E-05	0.00110376
AC010624.1	111.720814	2.63860605	0.41258334	6.39532858	1.60E-10	2.07E-08
AC010624.4	14.5994018	2.09947174	0.62057325	3.38311673	0.00071668	0.00740786
AC010624.2	12.7589219	2.01426417	0.7230591	2.78575315	0.00534035	0.03309471
AC074141.1	156.006395	1.28924445	0.20518197	6.28341977	3.31E-10	3.76E-08
ZNF528-AS1	330.357687	1.12859049	0.19274929	5.85522501	4.76E-09	3.97E-07
FAM90A27P	68.5540603	2.02658474	0.5880571	3.44623802	0.00056845	0.00622982
FAM90A28P	25.905195	3.06809414	0.72375998	4.23910445	2.24E-05	0.00048967
TMC4	61.8603291	1.23557404	0.41191889	2.99955665	0.00270373	0.02003918
RFPL4A	79.9511936	1.90245752	0.61782236	3.07929533	0.00207491	0.01653561
RFPL4AL1	78.6937668	1.77161641	0.54898509	3.22707566	0.00125062	0.01128095
NLRP4	30.0161222	1.73956208	0.59702876	2.91369898	0.00357174	0.02468654
ZSCAN4	714.246437	3.10866482	0.72702133	4.27589218	1.90E-05	0.00043046
C19orf18	36.9178145	1.10659056	0.30681325	3.6067235	0.00031009	0.003867
RPS15AP36	13.4581683	1.44462969	0.44406323	3.25320716	0.0011411	0.01052498
AP000547.3	5.64062378	2.77082932	0.98453629	2.81434962	0.0048876	0.03096878
ZNF280A	216.947563	1.23303692	0.27506698	4.48267884	7.37E-06	0.00019567
POM121L9P	10.8020191	2.30030339	0.68157106	3.37500158	0.00073815	0.00757154
GGT1	187.36774	1.64331188	0.32462137	5.06224178	4.14E-07	1.84E-05
AL008721.2	220.618533	1.19191735	0.22403542	5.32021827	1.04E-07	5.84E-06
MIAT	74.190758	1.002294	0.29950781	3.34647034	0.00081847	0.0081623
EMID1	187.905578	1.03057783	0.36095213	2.8551648	0.00430145	0.02826186
RFPL1S	75.501311	1.02408594	0.24748553	4.13796284	3.50E-05	0.00068572
LIF	387.069707	1.82982503	0.30021701	6.09500782	1.09E-09	1.09E-07
AC004264.1	17.2537135	1.77546728	0.67516475	2.62968007	0.00854653	0.04647027
GAL3ST1	33.0674765	1.41852191	0.50782125	2.79334887	0.00521654	0.03253069
SMTN	2113.06567	1.50645074	0.2326892	6.47408978	9.54E-11	1.35E-08
APOL2	1081.39445	1.27366838	0.4888576	2.60539751	0.00917677	0.04907774
ELFN2	90.9946267	2.40701452	0.5917397	4.06769143	4.75E-05	0.00086774
AL031587.5	88.7660949	1.60085746	0.25667143	6.23699133	4.46E-10	4.87E-08
C22orf23	96.5788822	1.50229422	0.34210587	4.39131379	1.13E-05	0.00028096
AL031587.3	17.8578917	3.96871997	0.75413212	5.26263219	1.42E-07	7.65E-06
APOBEC3C	209.344507	1.06090078	0.2090591	5.0746454	3.88E-07	1.74E-05
APOBEC3G	372.87494	1.23418764	0.38812317	3.17988655	0.00147333	0.01275208
FAM83F	58.9477525	2.1981247	0.57140528	3.84687501	0.00011963	0.00181784

MCHR1	24.2496264	1.23052066	0.42368107	2.90435598	0.00368009	0.02524353
L3MBTL2-AS1	20.1631646	1.34876536	0.39186499	3.44191335	0.00057762	0.00630493
CHADL	28.9483139	1.63589551	0.4777087	3.42446247	0.00061602	0.00660641
CSDC2	165.010565	1.15388073	0.30119222	3.83104422	0.0001276	0.00190441
MEI1	27.6482013	1.09083597	0.37591033	2.9018515	0.00370964	0.02541426
BIK	64.6801322	1.93875833	0.37289754	5.19917162	2.00E-07	1.02E-05
WNT7B	19.9633267	3.08393432	0.99419805	3.10193157	0.00192262	0.01562445
AL023802.1	16.4043088	1.75949378	0.62881367	2.7981163	0.00514016	0.03217441
SIK1B	50.9142342	1.29892866	0.32117698	4.04427691	5.25E-05	0.00093794
RPL39P40	74.9080075	1.79132892	0.49582978	3.61279007	0.00030292	0.00380087
RPL23P2	28.1778691	1.54437196	0.38282447	4.03415159	5.48E-05	0.0009643
DSCR4	21.928038	2.47639126	0.83686949	2.95911285	0.00308526	0.02209399
RSPH1	32.1567803	1.50534171	0.44936407	3.34993784	0.0008083	0.00810153
ICOSLG	18.9959825	2.36019365	0.52675716	4.48061046	7.44E-06	0.00019709
AP001065.3	18.3201137	2.39008361	0.74396394	3.2126337	0.00131524	0.01168265
AP001066.1	25.4152207	2.18816724	0.68249287	3.20613936	0.00134529	0.01187948
MT-TF	207.616751	1.82926312	0.20053339	9.1219877	7.38E-20	5.55E-17
MT-RNR1	34609.1767	1.20353943	0.14911094	8.07143622	6.95E-16	2.61E-13
MT-TV	130.746632	1.59272196	0.3316745	4.80206333	1.57E-06	5.53E-05
MT-RNR2	351657.812	1.58721696	0.13359787	11.8805559	1.49E-32	3.26E-29
MT-TN	2726.94647	1.13807189	0.13386017	8.50194572	1.86E-17	9.25E-15
MT-TC	2559.14194	1.39436425	0.14527741	9.59794279	8.16E-22	7.12E-19
MT-TY	2463.79775	1.30446366	0.10987487	11.8722659	1.65E-32	3.27E-29
MT-TP	2923.89277	1.75074177	0.14855318	11.7852864	4.65E-32	8.45E-29
HNRNPR	8604.25721	-1.0457045	0.15058981	-6.9440587	3.81E-12	7.56E-10
E2F2	956.436724	-1.112823	0.19240583	-5.7837284	7.31E-09	5.84E-07
CRYBG2	43.0596747	-1.3012327	0.38897863	-3.3452551	0.00082207	0.00818319
LIN28A	735.050499	-2.0790144	0.66019912	-3.1490717	0.0016379	0.01376694
CDC20	4527.08512	-1.298659	0.19179029	-6.7712446	1.28E-11	2.26E-09
SLC6A9	708.597048	-1.2957194	0.25169432	-5.1479881	2.63E-07	1.28E-05
ORC1	1325.81265	-1.0478908	0.26756429	-3.9164076	8.99E-05	0.00145557
HNRNPA3P12	17.3979189	-1.3487775	0.5022529	-2.6854549	0.00724311	0.04124341
PSRC1	1022.55088	-1.0294583	0.18578981	-5.5409837	3.01E-08	1.98E-06
PHGDH	2456.64812	-1.1619056	0.16923509	-6.8656306	6.62E-12	1.26E-09
FAM72C	42.3424133	-1.2005947	0.4426546	-2.7122607	0.0066826	0.03902541
FAM72D	79.9249013	-1.0886273	0.38906152	-2.7980851	0.00514066	0.03217441
H2AC20	21.9608814	-1.9272112	0.61483073	-3.1345395	0.00172124	0.01428041
FCRLB	80.2583062	-1.0973873	0.38223825	-2.870951	0.00409239	0.02726593
DDR2	44.1690316	-1.2614305	0.3610636	-3.4936518	0.00047646	0.00541761

SMG7-AS1	21.5367378	-1.1737564	0.3807395	-3.0828332	0.0020504	0.01637619
ASPM	4708.19579	-1.0515889	0.26597753	-3.9536757	7.70E-05	0.00127737
KIF14	2559.39633	-1.0666441	0.21755815	-4.9027999	9.45E-07	3.64E-05
VASH2	163.449294	-1.1202184	0.31388944	-3.5688311	0.00035858	0.00431385
CENPF	10116.0885	-1.0679908	0.25821083	-4.1361192	3.53E-05	0.00069063
CENPA	820.382855	-1.0673674	0.26110322	-4.0879135	4.35E-05	0.00081455
DPYSL5	807.974377	-1.3995282	0.24684449	-5.6696756	1.43E-08	1.03E-06
BCYRN1	124.748767	-1.1810678	0.30886327	-3.823918	0.00013135	0.00194174
LDHAP7	155.917016	-1.1168128	0.23748062	-4.7027534	2.57E-06	8.16E-05
BUB1	4331.56407	-1.188503	0.30482175	-3.8990099	9.66E-05	0.00153386
POTEJ	5.91002212	-2.4258557	0.73449102	-3.3027711	0.00095734	0.00925123
POTEE	9.80495215	-1.9212938	0.62415497	-3.078232	0.00208233	0.01657657
CCNT2-AS1	101.90349	-1.212295	0.23140798	-5.2387776	1.62E-07	8.54E-06
MCM6	6541.95726	-1.3808498	0.26850331	-5.1427663	2.71E-07	1.31E-05
HNRNPKP2	16.3693779	-1.2522095	0.43360684	-2.8878915	0.00387834	0.0262404
AC078882.1	56.4067384	-1.5155025	0.53996808	-2.806652	0.00500593	0.03155252
NUP35	1119.40837	-1.0051729	0.25638167	-3.9206116	8.83E-05	0.00143396
SGO2	1748.26973	-1.3640525	0.31691746	-4.3041254	1.68E-05	0.00038861
AC012513.3	232.934133	-1.1086761	0.31409321	-3.5297679	0.00041592	0.0048506
PRRT3-AS1	47.5806966	-1.0761164	0.36662466	-2.9351991	0.00333334	0.0233905
AC090948.2	8.06662339	-1.8852498	0.65483838	-2.8789543	0.00398996	0.02677974
AC099332.1	17.7110481	-1.5060639	0.57933787	-2.5996296	0.00933244	0.04966607
AC006059.1	55.7111522	-1.0544058	0.3885195	-2.7139071	0.00664948	0.03892989
CAMKV	87.6111483	-1.654728	0.31525906	-5.2487882	1.53E-07	8.13E-06
RBM5-AS1	19.0793387	-1.4053455	0.5092146	-2.7598296	0.00578315	0.03509131
AC099667.1	8.25091122	-1.6793999	0.6394761	-2.6262121	0.0086341	0.04685304
DPPA2	1140.43866	-1.1584217	0.3104587	-3.731323	0.00019048	0.00264054
ALG1L	8.51597871	-2.4561622	0.91168578	-2.6940886	0.00705814	0.04064625
MCM2	3879.22298	-1.442207	0.22509667	-6.4070562	1.48E-10	1.95E-08
AC137695.3	14.4596959	-1.8875537	0.53170501	-3.5500018	0.00038523	0.00455099
STAG1	1873.7038	-1.1471153	0.20581265	-5.57359	2.50E-08	1.69E-06
UBQLN4P1	9.93062936	-1.8843858	0.61093816	-3.0844133	0.00203954	0.01633303
SIAH2-AS1	19.2295751	-2.1304776	0.6186793	-3.4435896	0.00057405	0.0062754
PLCH1	616.146124	-1.1070049	0.27035473	-4.0946387	4.23E-05	0.00079741
SMC4	7588.95883	-1.018032	0.26735496	-3.8077916	0.00014021	0.00205885
OTOL1	190.56622	-2.4016621	0.47872426	-5.0167963	5.25E-07	2.21E-05
LINC01192	48.1621254	-1.4847263	0.39888317	-3.7222085	0.00019749	0.00272388
AC068308.1	37.7024052	-1.1817014	0.45134704	-2.6181659	0.00884038	0.0476878
CAMK2N2	35.7298163	-1.1707235	0.35854354	-3.2652199	0.00109379	0.01019501

ACAP2-IT1	8.39339179	-1.9143336	0.60120127	-3.1841809	0.00145164	0.01262952
BDH1	186.224012	-1.3365537	0.37596696	-3.5549764	0.00037801	0.00448518
AC078852.2	5.76393828	-2.9483131	0.95086235	-3.1006728	0.00193081	0.01566185
MIR573	7.90278825	-3.0908395	0.88739558	-3.4830458	0.00049574	0.00558159
KLB	116.629488	-1.5405975	0.38831992	-3.9673409	7.27E-05	0.00121801
AC124016.3	37.0855431	-1.4528	0.33391411	-4.3508194	1.36E-05	0.00032773
CENPE	3668.2657	-1.0485661	0.2842507	-3.6888777	0.00022525	0.00300971
PLK4	2431.79418	-1.1143671	0.28858087	-3.8615416	0.00011267	0.00173749
HMGCS1	12631.9793	-1.2122914	0.22712122	-5.3376406	9.42E-08	5.38E-06
AC026704.1	14.9993145	-1.5055055	0.54397084	-2.7676218	0.00564669	0.03444846
AC008517.1	29.7693236	-1.3469266	0.34987064	-3.8497845	0.00011822	0.00180014
LINC02058	62.9183273	-1.5878514	0.58975744	-2.6923805	0.0070944	0.04073676
PTMAP2	43.410264	-1.0718632	0.30394743	-3.5264754	0.00042113	0.00489822
MIR1244-2	22.5672664	-1.6527911	0.53996278	-3.0609352	0.00220647	0.0172749
LMNB1	8672.54309	-1.2097201	0.22732902	-5.3214503	1.03E-07	5.82E-06
SOWAHA	46.4406043	-1.7267079	0.31779311	-5.4334341	5.53E-08	3.37E-06
FAT2	33.4670182	-1.0277532	0.32113244	-3.200403	0.00137236	0.01205736
HMMR	2767.22174	-1.2691414	0.33364142	-3.8039085	0.00014243	0.00208151
H2AC14	6.417181	-4.7905384	1.04232269	-4.5960224	4.31E-06	0.00012495
MDC1	3073.36598	-1.0438041	0.13113266	-7.9599094	1.72E-15	6.16E-13
ZBTB12	364.65042	-1.3507476	0.23836715	-5.6666683	1.46E-08	1.05E-06
MCM3	7743.99998	-1.2282787	0.23591005	-5.2065554	1.92E-07	9.95E-06
TINAG	97.1055754	-1.1752652	0.40747421	-2.8842689	0.00392324	0.02645396
TTK	2154.61243	-1.002339	0.31997724	-3.1325323	0.00173305	0.01435113
AL358852.1	9.63740778	-1.7641073	0.64797877	-2.722477	0.00647945	0.03819062
SOD2-OT1	33.9832568	-1.0257964	0.38283501	-2.6794737	0.0073738	0.04183471
BX322234.1	26.6251144	-1.0416551	0.32312775	-3.2236635	0.00126562	0.01135335
CYP51A1	294.792456	-1.1277038	0.3027742	-3.7245703	0.00019565	0.00270022
MCM7	12144.2334	-1.3689454	0.18902772	-7.2420352	4.42E-13	1.05E-10
VGF	2541.99885	-1.3165056	0.24378072	-5.4003682	6.65E-08	3.94E-06
LHFPL3-AS2	8.41342577	-1.8597219	0.60189115	-3.0897977	0.00200293	0.01610907
NOS3	155.588774	-1.102942	0.27220132	-4.0519349	5.08E-05	0.00091449
AC144652.1	118.764775	-1.1759773	0.21731847	-5.4113083	6.26E-08	3.76E-06
INSIG1	4434.13924	-1.1296765	0.24426789	-4.6247444	3.75E-06	0.00011165
POLA1	2028.18815	-1.04382	0.265513	-3.9313328	8.45E-05	0.00138176
PFKFB1	18.5511222	-1.3621536	0.49374358	-2.7588281	0.0058009	0.03515993
SERBP1P1	23.6038076	-1.3886961	0.45393271	-3.0592555	0.00221888	0.01734546
DUSP9	101.377633	-1.5413886	0.32766213	-4.7042013	2.55E-06	8.13E-05
AC245140.2	50.6827068	-1.0337362	0.34494793	-2.9967892	0.00272839	0.02017402

TUBBP1	50.1142038	-1.2355773	0.39394178	-3.1364465	0.00171009	0.01423115
CASC11	6.58061516	-1.7674718	0.67361152	-2.6238741	0.00869359	0.04711728
RBMXP2	14.884735	-1.8033058	0.54167394	-3.3291352	0.00087116	0.0085857
SNX18P7	36.4813955	-1.3993667	0.41474626	-3.374031	0.00074076	0.00759095
AL591438.1	102.098296	-1.2740725	0.42071917	-3.0283206	0.00245917	0.01874226
CDC20P1	85.8108007	-1.0229986	0.27038744	-3.7834545	0.00015467	0.00222174
OGN	124.167724	-1.5585185	0.45865529	-3.3980172	0.00067876	0.00711022
PHF19	1648.20405	-1.0706162	0.16163624	-6.6236146	3.51E-11	5.54E-09
SAPCD2	216.359071	-1.803344	0.23494941	-7.6754567	1.65E-14	5.37E-12
E2F8	604.272143	-1.3554177	0.23957074	-5.6576928	1.53E-08	1.09E-06
LARGE2	232.773363	-2.0533654	0.32834353	-6.2537105	4.01E-10	4.46E-08
FADS2	10251.9842	-1.1410824	0.17949974	-6.3570141	2.06E-10	2.52E-08
FOLR1	6.26225318	-2.3770752	0.73242198	-3.2454995	0.00117245	0.01075812
BX322639.1	1075.67166	-1.0399513	0.18825868	-5.5240551	3.31E-08	2.16E-06
AC016397.2	7.62561271	-2.0435955	0.75785608	-2.6965483	0.00700622	0.04041126
TET1	871.271129	-1.1139074	0.20979006	-5.3096291	1.10E-07	6.16E-06
AL353751.1	283.58641	-1.2097863	0.45945409	-2.6330951	0.00846107	0.0461782
KIF11	5717.9067	-1.1402885	0.27806544	-4.1007919	4.12E-05	0.00078217
DCLRE1A	1174.88918	-1.0326731	0.25954356	-3.9788044	6.93E-05	0.00117065
MKI67	12417.9317	-1.3414362	0.22712336	-5.9062009	3.50E-09	3.02E-07
AC125494.1	7.64932079	-2.6860487	0.98399662	-2.7297336	0.00633855	0.03754267
SCARNA12	12.690438	-1.2000251	0.44914029	-2.6718269	0.00754396	0.04243597
KLRC1	657.140707	-1.2642676	0.32083732	-3.9405255	8.13E-05	0.00133991
PIK3C2G	13.5372195	-2.2385323	0.70942768	-3.1554059	0.00160275	0.01356029
AC112777.1	205.458269	-1.6055853	0.26294247	-6.1062227	1.02E-09	1.03E-07
BCAT1	5671.87232	-1.254058	0.30765559	-4.076175	4.58E-05	0.00084533
AC023158.2	75.7167048	-1.8930095	0.40754611	-4.6448965	3.40E-06	0.00010311
DDN	112.272539	-1.3128719	0.2820475	-4.65479	3.24E-06	9.91E-05
TUBA1B	9938.33615	-1.177658	0.21766672	-5.4103723	6.29E-08	3.77E-06
AC125611.4	18.6864584	-1.3122493	0.4386946	-2.9912592	0.0027783	0.02048206
HOXC-AS2	226.666709	-1.106411	0.17797541	-6.2166508	5.08E-10	5.46E-08
TMPO	10309.9432	-1.0533391	0.21585195	-4.8799146	1.06E-06	4.00E-05
NR1H4	139.673495	-1.8124382	0.33801118	-5.3620658	8.23E-08	4.77E-06
AC079907.1	9.48335337	-2.2044545	0.78551505	-2.806381	0.00501014	0.03156787
AC004263.1	8.29138844	-2.6743774	0.84828577	-3.1526845	0.00161777	0.01363447
DLEU2	211.795461	-1.0513344	0.2097512	-5.0122926	5.38E-07	2.26E-05
LINC00348	586.730954	-1.6928364	0.37345565	-4.532898	5.82E-06	0.00015908
MIR17HG	214.744151	-1.0493319	0.33078093	-3.1722865	0.00151244	0.01303887
LINC01551	730.982519	-1.2247198	0.21866372	-5.6009282	2.13E-08	1.46E-06

FRMD6-AS1	20.0602411	-1.1621523	0.40305308	-2.8833728	0.00393441	0.02650792
DLGAP5	4529.38695	-1.0573605	0.29549431	-3.5782771	0.00034587	0.00420435
ATP10A	24.3275159	-1.1688916	0.39299386	-2.9743253	0.00293634	0.02134983
ARHGAP11A	3758.17053	-1.0691283	0.27558974	-3.8794199	0.00010471	0.00164247
FSIP1	127.248498	-1.3942628	0.46429997	-3.0029353	0.00267389	0.01985177
BUB1B	3881.76905	-1.2948962	0.25259337	-5.126406	2.95E-07	1.41E-05
WDR76	2117.12985	-1.2069215	0.25299285	-4.7705754	1.84E-06	6.27E-05
AC023355.2	56.0927661	-1.1031123	0.28057295	-3.9316416	8.44E-05	0.00138102
PIF1	498.485988	-1.1315075	0.2300203	-4.9191635	8.69E-07	3.39E-05
PRC1	229.458531	-1.0434136	0.23745826	-4.3940925	1.11E-05	0.00027867
LINC00235	49.69322	-1.1925359	0.2760889	-4.3193908	1.56E-05	0.00036828
MIR3176	6.0090626	-2.1678554	0.81405606	-2.6630296	0.00774406	0.04316102
AL031710.2	10.8019626	-1.7294063	0.560318	-3.0864728	0.00202546	0.01627107
MEIOB	466.337074	-1.4134784	0.27475292	-5.1445437	2.68E-07	1.30E-05
PKMYT1	805.018272	-1.2410673	0.20575437	-6.0317903	1.62E-09	1.57E-07
PAQR4	594.968072	-1.2797986	0.22254453	-5.7507531	8.88E-09	6.87E-07
AC009121.3	21.4027023	-1.1128989	0.39544112	-2.8143226	0.00488802	0.03096878
PLK1	3271.35787	-1.730156	0.25486467	-6.7885281	1.13E-11	2.08E-09
AC023824.5	91.5962733	-1.1689316	0.36851013	-3.1720476	0.00151368	0.01304445
MT1E	44.2670872	-1.9878814	0.5193942	-3.8273076	0.00012955	0.00192171
MT1F	20.7152245	-1.5518215	0.56031886	-2.7695329	0.00561367	0.03430142
AC009053.4	17.0874354	-1.2805969	0.46271158	-2.767592	0.00564721	0.03444846
AC040169.3	5.59029118	-1.9959503	0.74823927	-2.6675295	0.00764112	0.04281695
GINS2	1527.06422	-1.282537	0.26339352	-4.8692806	1.12E-06	4.17E-05
AC032044.1	12.700075	-1.3742556	0.51982489	-2.6436894	0.00820079	0.04506199
SLC2A4	76.5248109	-1.3575249	0.274152	-4.9517235	7.36E-07	2.97E-05
AC020558.6	6.54986118	-2.5174466	0.81957035	-3.0716663	0.00212868	0.01681577
STAC2	124.249922	-1.1339992	0.23631064	-4.798765	1.60E-06	5.61E-05
MEOX1	481.855702	-2.2290646	0.81706367	-2.7281407	0.00636924	0.03766312
AC005180.1	6.39676711	-3.1675645	0.77631985	-4.0802312	4.50E-05	0.00083549
SRSF1	4798.60642	-1.1539689	0.18500391	-6.2375381	4.45E-10	4.87E-08
TRIM47	540.323033	-1.246286	0.22437933	-5.5543711	2.79E-08	1.85E-06
AC110285.6	79.7733122	-1.8820959	0.40189735	-4.6830263	2.83E-06	8.84E-05
PCYT2	1799.24502	-1.1161245	0.21320028	-5.2350985	1.65E-07	8.69E-06
PYCR1	1590.54171	-1.1766029	0.18316754	-6.423643	1.33E-10	1.80E-08
FASN	19838.4423	-1.3499377	0.23102103	-5.8433542	5.12E-09	4.20E-07
HNRNPA1P7	23.8063498	-1.0510954	0.39028994	-2.6931141	0.0070788	0.0407218
RBL1	1067.5303	-1.3174054	0.21255353	-6.1979932	5.72E-10	6.09E-08
TUBB1	68.209301	-1.2555646	0.30680457	-4.092392	4.27E-05	0.0008031

SLCO4A1	314.403677	-1.5906385	0.33249308	-4.7839748	1.72E-06	5.93E-05
RTEL1-TNFRSF6B	115.928411	-1.0480047	0.31256985	-3.3528655	0.0007998	0.00803478
C20orf204	17.1107851	-1.5650955	0.55015363	-2.8448335	0.00444346	0.02883033
AC009005.1	120.696871	-1.003326	0.28760634	-3.4885393	0.00048567	0.00549936
KISS1R	7.82701808	-3.3610316	1.00993745	-3.3279602	0.00087484	0.00860644
AC027307.3	32.5264055	-1.5789146	0.3953799	-3.9934114	6.51E-05	0.00110939
AC012615.6	66.8517566	-1.0468206	0.36226558	-2.8896497	0.00385671	0.02612651
MATK	43.9725005	-1.4510801	0.49069191	-2.9572123	0.00310434	0.02219422
UHRF1	3366.33015	-1.6523217	0.21335959	-7.744305	9.61E-15	3.18E-12
DNMT1	7647.76309	-1.0156682	0.16196727	-6.2708239	3.59E-10	4.06E-08
RAVER1	1182.11878	-1.074592	0.27854681	-3.8578505	0.00011439	0.00175956
RNASEH2A	38.1744759	-1.0288855	0.31492875	-3.2670421	0.00108678	0.01016381
PALM3	1484.15389	-1.1480808	0.19921927	-5.7629006	8.27E-09	6.44E-07
AC024075.1	32.7024393	-1.1585784	0.34245186	-3.3831861	0.0007165	0.00740786
AC074135.1	234.307548	-1.0788852	0.23404998	-4.6096358	4.03E-06	0.00011862
LINC01529	43.3613624	-1.4015879	0.32677894	-4.2891012	1.79E-05	0.00041032
MAMSTR	878.662512	-1.0395518	0.20408887	-5.093623	3.51E-07	1.60E-05
GFY	60.4675122	-1.8453826	0.57312585	-3.2198558	0.00128255	0.01145529
PRMT1	7683.09744	-1.0162001	0.16785556	-6.054015	1.41E-09	1.37E-07
ADM5	41.6637025	-1.4505606	0.45553093	-3.1843296	0.0014509	0.01262807
SIGLEC11	74.2855313	-1.1516621	0.40187051	-2.8657541	0.00416017	0.02759118
POLD1	1876.60074	-1.0498709	0.20869567	-5.0306307	4.89E-07	2.09E-05
LILRB3	13.3067567	-2.0974567	0.73109912	-2.8689088	0.00411891	0.02741748
MTFP1	98.3554264	-1.0008645	0.24717709	-4.0491797	5.14E-05	0.00092304
AC005005.3	32.6845455	-1.2195577	0.42265429	-2.8854734	0.00390825	0.02636108
MCM5	5028.47456	-1.4462315	0.22833355	-6.3338547	2.39E-10	2.87E-08
H1-0	16775.4683	-1.4470308	0.19165438	-7.55021	4.35E-14	1.25E-11
OLA1P1	21.0332784	-1.050761	0.38336477	-2.7408909	0.00612728	0.03665954
AL079301.1	9.75659638	-2.3359553	0.63472211	-3.6802803	0.00023298	0.00308193
CBSL	246.891405	-1.4173573	0.28044764	-5.0539104	4.33E-07	1.91E-05
RIMKLBP1	10.1739284	-1.7051617	0.63178843	-2.6989441	0.00695599	0.04020652
WDR4	839.393405	-1.0065812	0.17013668	-5.9163092	3.29E-09	2.86E-07
CBS	101.495187	-1.2590813	0.32943991	-3.8218844	0.00013244	0.00195518
AP001469.1	14.4620232	-1.5009357	0.49970701	-3.0036314	0.00266778	0.01982665

Table S4. Gene set enrichment analysis using hallmark gene sets on MTX- vs MTX+ SCCOHT tumoroids.

pathway	pval	padj	log2err	ES	NES
HALLMARK_P53_PATHWAY	3.07E-37	3.83E-36	1.58945578	0.71018112	2.96881981
HALLMARK_TNFA_SIGNALING_VIA_NFKB	1.01E-18	6.30E-18	1.11466448	0.60418654	2.50888318
HALLMARK_COAGULATION	4.63E-09	2.10E-08	0.7614608	0.55239246	2.16208059
HALLMARK_HYPOXIA	2.49E-09	1.25E-08	0.77493903	0.4917526	2.0468529
HALLMARK_APOPTOSIS	8.47E-08	3.53E-07	0.70497572	0.49170954	1.98704144
HALLMARK_IL6_JAK_STAT3_SIGNALING	2.76E-05	7.93E-05	0.57561026	0.53618699	1.94718838
HALLMARK_EPITHELIAL_MESENCHYMAL_TRANSITION	1.76E-07	6.30E-07	0.69013246	0.45879168	1.91791896
HALLMARK_INFLAMMATORY_RESPONSE	6.66E-05	0.00017039	0.5384341	0.42697313	1.75064333
HALLMARK_MYOGENESIS	1.24E-05	4.13E-05	0.59332548	0.4207249	1.74595079
HALLMARK_COMPLEMENT	6.82E-05	0.00017039	0.5384341	0.41683012	1.72708485
HALLMARK_INTERFERON_GAMMA_RESPONSE	2.86E-05	7.93E-05	0.57561026	0.40979418	1.703161
HALLMARK_ALLOGRAFT_REJECTION	0.00081858	0.00177951	0.47727082	0.39629101	1.60414558
HALLMARK_IL2_STAT5_SIGNALING	0.00071918	0.00171233	0.47727082	0.37640948	1.56591426
HALLMARK_HEME_METABOLISM	0.00116073	0.00232146	0.45505987	0.37182998	1.54230007
HALLMARK_KRAS_SIGNALING_UP	0.00104794	0.00218321	0.45505987	0.37016519	1.5347257
HALLMARK_KRAS_SIGNALING_DN	0.00332492	0.00615726	0.4317077	0.36940663	1.5066901
HALLMARK_APICAL_SURFACE	0.02915121	0.04701808	0.35248786	0.44807183	1.46459434
HALLMARK_ANGIOGENESIS	0.0622084	0.08886914	0.22496609	0.46550831	1.41399921
HALLMARK_ESTROGEN_RESPONSE_EARLY	0.01427115	0.02460542	0.3807304	0.3325809	1.38762902
HALLMARK_PANCREAS_BETA_CELLS	0.07378336	0.09970724	0.20658792	0.44275002	1.36209352
HALLMARK_INTERFERON_ALPHA_RESPONSE	0.04815864	0.07082153	0.24504179	0.35769496	1.34884585
HALLMARK_XENOBIOTIC_METABOLISM	0.03337612	0.05215019	0.28201335	0.32211146	1.33462964
HALLMARK_TGF_BETA_SIGNALING	0.09848485	0.12626263	0.17374784	0.38138262	1.30839545
HALLMARK_UV_RESPONSE_UP	0.068	0.09444444	0.19782202	0.31213892	1.2620198
HALLMARK_HEDGEHOG_SIGNALING	0.19339623	0.23584906	0.12210792	0.38377701	1.19675263
HALLMARK_APICAL_JUNCTION	0.1994852	0.23748238	0.10672988	0.27279848	1.13548633
HALLMARK_BILE_ACID_METABOLISM	0.33518776	0.35658272	0.08108021	0.28136867	1.07335244
HALLMARK_DNA_REPAIR	0.32712766	0.35557354	0.07998588	0.26571867	1.06812404
HALLMARK_ESTROGEN_RESPONSE_LATE	0.3162612	0.35140134	0.07977059	0.25534016	1.06029943
HALLMARK_NOTCH_SIGNALING	0.42386185	0.44152276	0.07511816	0.33678871	1.03611
HALLMARK_UV_RESPONSE_DN	0.45285525	0.46209719	0.0635008	0.25056868	1.00446715

HALLMARK_WNT_BETA_CATENIN_SIGNALING	0.6259542	0.6259542	0.05479395	0.27195517	0.88892891
HALLMARK_PEROXISOME	0.28469751	0.3235199	0.15524197	-0.2526515	-1.0890814
HALLMARK_REACTIVE_OXYGEN_SPECIES_PATHWAY	0.25588235	0.29753762	0.1482615	-0.2965992	-1.1198784
HALLMARK_PROTEIN_SECRETION	0.14685315	0.18356643	0.21925035	-0.2707595	-1.167243
HALLMARK_SPERMATOGENESIS	0.0866125	0.11396381	0.28780513	-0.2790881	-1.223482
HALLMARK_GLYCOLYSIS	0.03534336	0.05355055	0.32177592	-0.2603031	-1.2299576
HALLMARK_ADIPOGENESIS	0.02090339	0.03483899	0.35248786	-0.2777502	-1.3137927
HALLMARK_FATTY_ACID_METABOLISM	0.00652301	0.01164823	0.40701792	-0.3071984	-1.4136857
HALLMARK_ANDROGEN_RESPONSE	0.00142647	0.00274321	0.45505987	-0.3852304	-1.6736226
HALLMARK_PI3K_AKT_MTOR_SIGNALING	0.00076587	0.00174061	0.47727082	-0.3966565	-1.7232627
HALLMARK_OXIDATIVE_PHOSPHORYLATION	1.28E-07	4.91E-07	0.69013246	-0.4037524	-1.9123404
HALLMARK_CHOLESTEROL_HOMEOSTASIS	1.43E-05	4.45E-05	0.59332548	-0.4978809	-2.0327412
HALLMARK_UNFOLDED_PROTEIN_RESPONSE	1.58E-10	8.78E-10	0.8266573	-0.544991	-2.3937983
HALLMARK_MITOTIC_SPINDLE	6.45E-20	4.61E-19	1.15122052	-0.5592492	-2.6386787
HALLMARK_MTORC1_SIGNALING	1.61E-23	1.34E-22	1.25451348	-0.5904314	-2.785804
HALLMARK_MYC_TARGETS_V2	8.95E-24	8.95E-23	1.26273989	-0.8380286	-3.2796513
HALLMARK_E2F_TARGETS	1.00E-50	1.67E-49	NA	-0.7615299	-3.6069246
HALLMARK_MYC_TARGETS_V1	1.00E-50	1.67E-49	NA	-0.7738994	-3.6655115
HALLMARK_G2M_CHECKPOINT	1.00E-50	1.67E-49	NA	-0.7867834	-3.7265356

Table S5. A list of 19 pediatric tumor tissues used in RNA sequencing analysis.

**Patient identifier**	**Tumor Name**
PMABM000BEO	Osteosarcoma, NOS
PMABM000BEW	Craniopharyngioma, adamantinomatous
PMABM000BHJ	Glioma, malignant
PMABM000BJS	Hepatocellular carcinoma, fibrolamellar
PMABM000BQK	Ependymoma, NOS
PMABM000BST	Embryonal rhabdomyosarcoma, NOS
PMABM000BWW	Hepatocellular carcinoma, fibrolamellar
PMABM000CAW	Osteosarcoma, NOS
PMABM000CCI	Ependymoma, NOS
PMABM000CDK	Primitive neuroectodermal tumor, NOS
PMABM000COX	Ewing sarcoma
PMABM000CRP	Rhabdomyosarcoma, NOS
PMABM000CUV	Alveolar rhabdomyosarcoma
PMABM000CZA	Hepatoblastoma
PMABM000DAQ	Craniopharyngioma, adamantinomatous
PMABM000DAU	Alveolar rhabdomyosarcoma
PMABM000DBC	Ependymoma, anaplastic
PMABM000DEQ	Embryonal rhabdomyosarcoma, NOS
PMABM000DGO	Alveolar rhabdomyosarcoma

Table S6. A list of primers used in this study.

Target gene	Direction	Sequence (5' to 3')
SMARCA4	Forward	CGCTACAACCAGATGAAAGGA
SMARCA4	Reverse	TGGCTGGAACTGGACTAGA
SMARCA2	Forward	AGAGCGGATTGAAAAGGAGAG
SMARCA2	Reverse	ATTGGCTACATACTCATCGGTC
GAPDH	Forward	CTCCTGTTCGACAGTCAGCC
GAPDH	Reverse	ACCAAATCCGTTGACTCCGAC
HPRT	Forward	GGCGAACCTCTCGGCTTT
HPRT	Reverse	ATCACTAATCACGACGCCAG
